# Next Generation Sequencing to Define Prokaryotic and Fungal Diversity in the Bovine Rumen

**DOI:** 10.1371/journal.pone.0048289

**Published:** 2012-11-07

**Authors:** Derrick E. Fouts, Sebastian Szpakowski, Janaki Purushe, Manolito Torralba, Richard C. Waterman, Michael D. MacNeil, Leeson J. Alexander, Karen E. Nelson

**Affiliations:** 1 The J. Craig Venter Institute (JCVI), Rockville, Maryland, United States of America; 2 Fort Keogh Livestock and Range Research Laboratory, USDA Agricultural Research Service, Miles City, Montana, United States of America; Fordham University, United States of America

## Abstract

A combination of Sanger and 454 sequences of small subunit rRNA loci were used to interrogate microbial diversity in the bovine rumen of 12 cows consuming a forage diet. Observed bacterial species richness, based on the V1–V3 region of the 16S rRNA gene, was between 1,903 to 2,432 species-level operational taxonomic units (OTUs) when 5,520 reads were sampled per animal. Eighty percent of species-level OTUs were dominated by members of the order *Clostridiales*, *Bacteroidales*, *Erysipelotrichales* and unclassified TM7. Abundance of *Prevotella* species varied widely among the 12 animals. Archaeal species richness, also based on 16S rRNA, was between 8 and 13 OTUs, representing 5 genera. The majority of archaeal OTUs (84%) found in this study were previously observed in public databases with only two new OTUs discovered. Observed rumen fungal species richness, based on the 18S rRNA gene, was between 21 and 40 OTUs with 98.4–99.9% of OTUs represented by more than one read, using Good’s coverage. Examination of the fungal community identified numerous novel groups. *Prevotella* and *Tannerella* were overrepresented in the liquid fraction of the rumen while *Butyrivibrio* and *Blautia* were significantly overrepresented in the solid fraction of the rumen. No statistical difference was observed between the liquid and solid fractions in biodiversity of archaea and fungi. The survey of microbial communities and analysis of cross-domain correlations suggested there is a far greater extent of microbial diversity in the bovine rumen than previously appreciated, and that next generation sequencing technologies promise to reveal novel species, interactions and pathways that can be studied further in order to better understand how rumen microbial community structure and function affects ruminant feed efficiency, biofuel production, and environmental impact.

## Introduction

The bovine rumen harbors a diverse population of microorganisms that converts ingested plant biomass to protein, short chain volatile fatty acids, and gases (e.g., CO_2_, NH_3_, and CH_4_) via fermentation. End-products of rumen microbial fermentation provide the host with essential nutrients for metabolism, but are also released into the environment. Studying the microbial populations associated with the bovine gastrointestinal tract (GIT) holds vast potential for answering questions associated with improving animal production [1] and increasing the efficiency of animal feed [2], [3]. Additionally, it stands to foster an understanding of the impact of the host on GIT bacterial populations [4]. The ultimate implications of these studies include improving renewable fuel production, including conversion of cellulosic waste to biogas [5], and reduction of greenhouse gas production and emissions.

The bovine rumen microbiome is estimated to contain more than 10^10^ bacteria, 10^9^ phage, 10^8^ protozoa, 10^7^ archaea, and 10^3^ fungal spores per ml [6], [7]. Both 18S and 16S small subunit (SSU) rRNA surveys of the bovine rumen suggest extensive microbial diversity of both eukaryotic and prokaryotic fractions, far greater than has been suggested using traditional culturing methods [8], [9], [10], [11], [12]. Estimates of the number of rumen microbial species based on 16S rRNA gene sequences vary from 300–400 with Sanger [10] to 500–1000 [12], [13], [14], and 12000 [11] with 454 pyrosequencing. By pooling rumen bacterial 16S rRNA data from the RDP database, Kim *et al.* calculated 5271 species-level operational taxonomic units (OTUs) [15]. Low G + C Gram-positive bacteria (54%) and the *Cytophaga-Flexibacter-Bacteroides* genera (40%) appear as the most abundant *Bacteria* [16]; *Archaea*, particularly methanogens, are estimated to comprise approximately 0.3–3% of the biomass [9], [17]. Brulc *et al.*, [13] identified a limited number of eukaryotic phylotypes (≈1.3%), most of which were similar to *Viridiplantae* (i.e., plant feed), *Metazoa* (i.e., bovine), and *Fungi*.

With the advent of next generation sequencing technologies, it is now feasible to conduct in-depth sequencing and data analysis on samples derived from any environment of choice, including the rumen microbiota, at a deeper level than previously performed. Available rumen SSU-based microbiome studies have provided an incomplete picture of the microbial community structure, only focusing on one microbial domain at a time [11], [12], [13], [14], [18], [19]. In the present study, SSU rRNA sequencing was used to provide a comprehensive assessment of rumen microbial diversity, including *Bacteria*, *Archaea*, and *Fungi*. We compared data from 12 cows to previously identified rumen taxa from public repositories and found novel taxa from each microbial domain. To determine how microbial domains partitioned between solid and liquid fractions of bolus, microbial taxonomic profiles were compared per animal and between solid and liquid fractions. Integration of prokaryotic and fungal data sets highlighted the cross-domain correlations among the abundances of rumen inhabitants. A phylogenetic analysis of potentially novel fungal taxa was also presented.

## Results and Discussion

DNA from rumen solid or liquid material was extracted for PCR amplification with primers specific for prokaryotic 16S rRNA and fungal 18S rRNA genes. Sequences were trimmed for quality using LUCY [20], which has been shown to help reduce overestimation of OTUs [21] that commonly occur from 454 pyrosequencing errors [21], [22], [23]. All primer-trimmed sequences that passed the length cut-off were analyzed with MOTHUR [24], with a species-level OTU definition of 97% sequence identity (i.e., 3% divergence).

### Assessment of Current Publicly Available Ruminal SSU rRNA Sequences

NCBI, SILVA and RDP repositories of bacterial 16S sequences were queried to retrieve nucleotide sequences annotated as ruminal (see Materials and Methods for search terms) in order to compare new data to existing data. The query resulted in 22485, 12153 and 15637 sequences respectively (**Table S1**). The sequences from all three public repositories were combined to form a reference dataset (indicated as “REF” in **Table 1** and **Table S1).** These reference sequences from all three repositories were then aligned against a reference 16S sequence alignment. Based on this alignment (**Figure S1**), the V1–V3 region of the 16S sequence seemed to be slightly overrepresented in the public repositories. In general, the sequences deposited in the three repositories spanned the length of the entire 16S sequence. Overall, the public repositories contained 14332 unique sequences aligning to the V1–V3 region of the 16S gene (**Figure S1**). These previously discovered sequences were compared to the sequences generated in this study. Utilizing an OTU approach to cluster the publicly available sequences based on their sequence similarity, the public repositories contained approximately 4670 distinct, ruminal, species-level bacterial OTUs, a number slightly less than previously reported from the public domain [15]. This difference may be due to the way the reads were processed. In this study, only those reads mapping to the V1–V3 region were clustered, while Kim *et al*. did not single out a specific region, leaving the potential for reads originating from the same species, but mapping to different regions of the SSU rRNA gene, being clustered into different OTUs due to a lack of sequence overlap. In addition, the Kim *et al*. dataset was based on a multiple sequence alignment against the Greengenes database [25], while this study used CD-HIT [26] to determine sequence identity.

**Table 1:** Diversity among 12 cows and public repositories.

	Label	# of sequences	Distance	# of OTUs	Inv Simpson	Chao	Shannon	Shannon Evenness	Coverage
***Bacteria***	**COWS**	23,493	0.03	4,367	376 (339, 423)	11,176 (10,486, 11,943)	7.65 (7.62, 7.69)	0.91	67.12%
	**REF**	14,332	0.03	4,670	837 (793, 887)	9,479 (8,984, 10,030)	7.68 (7.66, 7.70)	0.91	81.43%
***Archaea***	**COWS**	138	0.03	20	6 (5, 7)	29 (22, 60)	2.10 (1.91, 2.30)	0.70	93.48%
	**REF**	2,484	0.03	486	17 (16, 19)	2,281 (1,717, 3,105)	4.22 (4.13, 4.30)	0.68	85.31%
***Eukarya***	**COWS**	2,089	0.03	52	4 (4, 5)	76 (60, 123)	2.16 (2.07, 2.24)	0.55	98.37%
	**REF**	859	0.03	168	10 (9, 12)	793 (505, 1,327)	3.38 (3.25, 3.50)	0.66	85.10%

Ruminal archaeal and eukaryotic SSU rRNA sequences were retrieved from public repositories using keyword searches similar to those used for bacteria (above). A total of 4198 ruminal archaeal SSU rRNA sequences in NCBI, 1120 in SILVA and 3703 in RDP were retrieved. A reference dataset, combining the sequences from the three repositories (“REF” in **Table 1**), contained 2484 unique sequences that aligned to the V1–V3 regions (**Figure S2**). Subsequent analysis of the 2484 sequences clustered at 97% identity detected 486 ruminal OTUs in the three repositories. A search for eukaryotic 18S sequences of the rumen in SILVA and NCBI databases yielded 1027 and 1803 sequences, respectively. Only approximately 10% of the eukaryotic sequences retrieved were annotated as fungal. RDP was not queried for eukaryotic sequences, as it does not house 18S rRNA sequences. Based on the alignment to the 18S reference sequence, a region matching the region sequenced in this study (**Figure S3**) yielded 168 OTUs.

Detailed results of the overlap of OTUs per repository for SSU rRNAs are presented in **Table S1** and **Figure S4.** For each microbial domain, NCBI consistently had the greatest number of unique OTUs, but not all-inclusive, justifying combining all three databases. Representative bacterial, archaeal, and eukaryotic OTU sequences from each repository were pooled to generate three reference datasets, which were subsequently used as a benchmark for diversity found in the current study.

### Current Study Versus Public Repositories

To determine if maximal microbial species-level OTU richness within the rumen has already been obtained, SSU rRNA was sequenced from rumen contents of 12 individual animals and compared to the same region (V1–V3) of the public repository OTU representative reads. Because there was variation in sequencing depths among the samples in this study, a random set of 5520 bacterial, 82 archaeal and 1046 fungal sequences were chosen based on the sample with the fewest sequence reads before entering the QC pipeline. Random subsampling of libraries to equalize the number of reads per sample has been suggested as one solution to obtaining unbiased non-parametric richness estimates of microbial community diversity from NextGen sequencing data [27]. Upon completion of the QC pipeline (see methods), a total of 23493, 138, and 2089 unique bacterial, archaeal, and fungal sequences were identified, respectively. The reads were subsequently clustered into 4367, 20, and 52 species-level OTUs, respectively **(** **Table 1** **)**. Good’s coverage [28], a measure of coverage of dominant taxa (i.e., those OTUs with more than one sequence), was 67% for *Bacteria*, and higher in *Archaea* (98%) and *Fungi* (100%), compare to 81–85% from the public repositories (**Table 1**). This disparity in coverage may be explained in part because the public repository sequences originated from a diverse group of ruminant animals (yellow cattle, reindeer, cattle, *etc*.) from different geographical locations. Except for *Bacteria*, species richness and evenness of the public domain dataset is greater than that observed from the animals in this study as noted by greater values of all four diversity estimators used.

**Figure 1:**
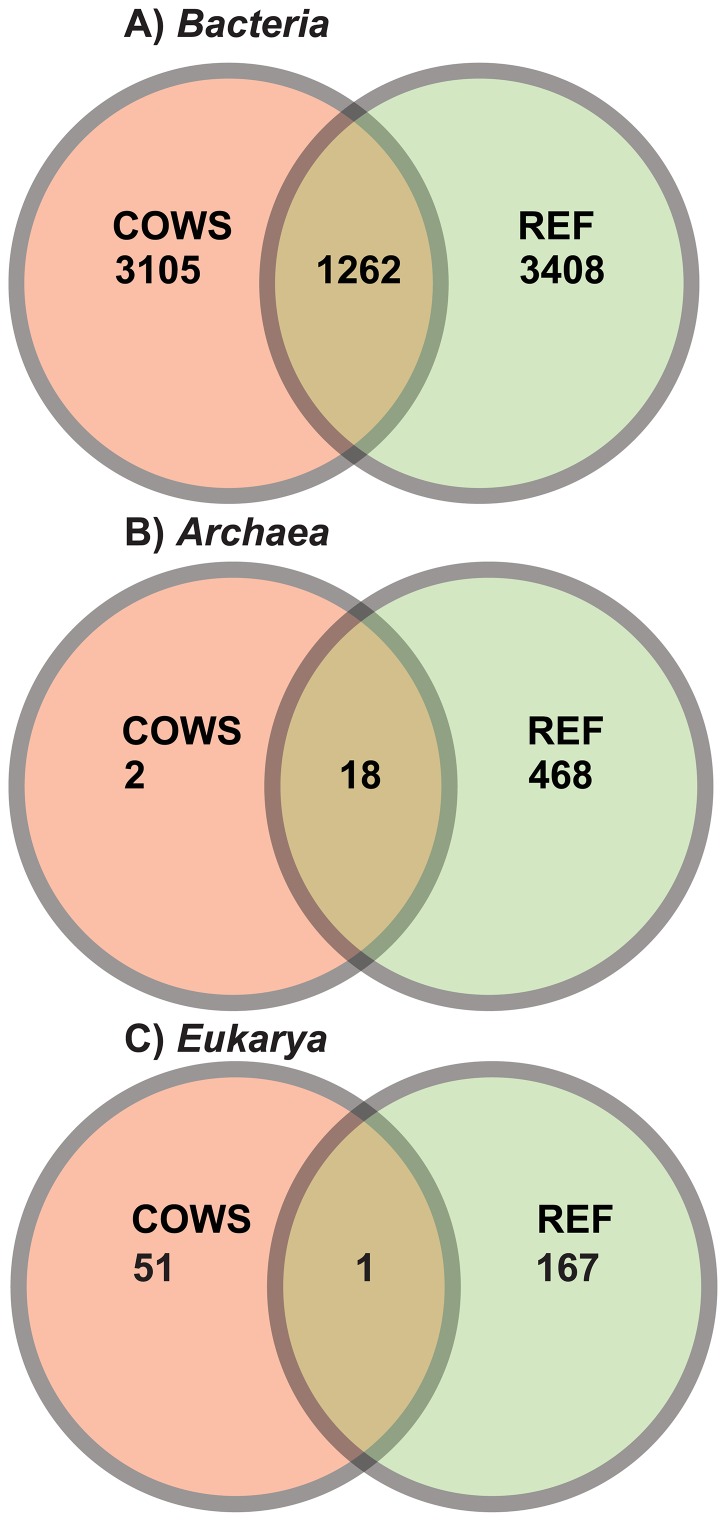
Comparison of rumen SSU microbial sequences to data in public repositories. The Venn diagram depicts OTUs that were unique to the 12 cows used in this study (COWS), unique to the public repositories (REF) or shared.

Out of 4367 bacterial species-level OTUs found in this study, only 1262 (29%) were shared with the three public repositories **(** **Figure 1A** **)**. Moreover, for every OTU found in the public repositories, a novel one was discovered from the new data, while still far from maximal Good’s coverage. Conversely, the majority of the archaeal OTUs (90%) found in this study were previously observed in the public databases with only 2 new OTUs added; 1 *Methanobrevibacter* and 1 *Thermogymnomonas*, (**Figure 1B**). For *Fungi*, only 1 OTU was shared between the public repositories and the animals investigated in this study, suggesting the scientific community is only beginning to realize the extent of fungal diversity of ruminants (**Figure 1C**). It should be noted that, of the 168 ruminal eukaryotic OTUs in public repositories, roughly 10% were fungal, which further highlights the paucity of rumen fungal rRNA sequences in these databases.

### Per-animal Bacterial Species Richness

**Figure 2:**
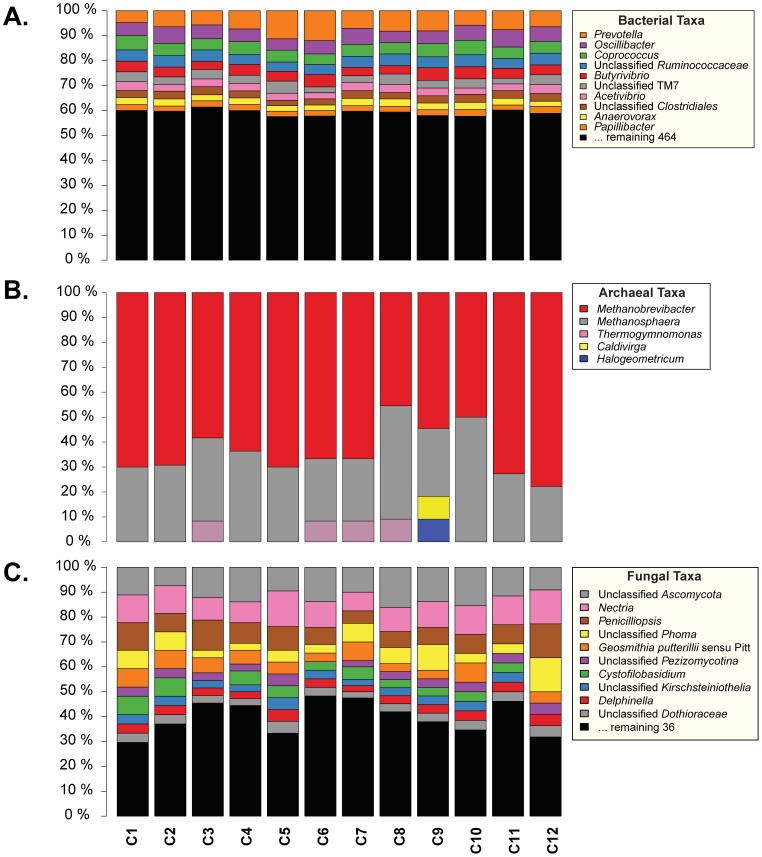
Bovine rumen microbial diversity among 12 cows. Bar-charts display the taxonomic profiles for *Bacteria* (A), *Archaea* (B), and *Fungi* (C) of OTUs counted at the genus level.

Bacterial 16S rRNA gene sequences from the solid and liquid fractions from each animal were pooled and sampled to generate OTU-based diversity calculations (**Table 2** **)**. As indicated in the “# of usable sequences” column of **Table 2**, almost all animals had at least 5199 LUCY-trimmed, unique, chimera-checked bacterial 16S sequences that were used in the final microbial species richness estimators. The observed number of OTUs ranged from 1903 to 2432 (**Table 2**). The Chao1 non-parametric richness estimator predicted as many as 3116 to 5439 species-level OTUs (**Table 2**). Good’s coverage was between 69–82%, indicating that by using ∼5000 bacterial sequences, the dominant bacterial community within the rumen of an individual animal was insufficiently sampled. Taxonomic profiling indicated that *Prevotella*, *Oscillibacter*, *Coprococcus*, unclassified *Ruminococcaceae*, and *Butyrivibrio* were the top five most abundant bacterial OTUs present in the rumen, comprising close to 40% of all bacterial taxa observed (**Figure 2A**).

**Table 2:** Sampling microbial diversity across 12 cows.

	Label	# Samples Reads	# of Usable Sequences	# of OTUs	Inv Simpson	Chao	Shannon	Shannon Evenness	Coverage
***Bacteria***	**C1**	**5520**	5374	1929	428 (388, 476)	3,551 (3,302, 3,845)	6.92 (6.89, 6.96)	0.92	80.22%
	**C2**	**5520**	5320	2295	690 (624, 772)	4,455 (4,158, 4,799)	7.22 (7.19, 7.26)	0.93	74.34%
	**C3**	**5520**	5359	1999	511 (472, 559)	3,762 (3,501, 4,068)	6.97 (6.93, 7.00)	0.92	78.50%
	**C4**	**5520**	5383	2159	597 (542, 664)	4,003 (3,739, 4,310)	7.12 (7.09, 7.16)	0.93	77.09%
	**C5**	**5520**	5454	1903	347 (312, 391)	3,388 (3,158, 3,660)	6.87 (6.83, 6.90)	0.91	81.19%
	**C6**	**5520**	5331	2407	874 (800, 963)	4,850 (4,522, 5,228)	7.31 (7.28, 7.35)	0.94	72.52%
	**C7**	**5520**	5199	2432	598 (535, 678)	5,439 (5,044, 5,893)	7.24 (7.20, 7.27)	0.93	69.21%
	**C8**	**5520**	5403	2046	670 (619, 730)	3,871 (3,598, 4,192)	7.08 (7.05, 7.12)	0.93	78.86%
	**C9**	**5520**	5371	2098	578 (529, 636)	3,976 (3,703, 4,295)	7.07 (7.03, 7.10)	0.92	77.55%
	**C10**	**5520**	5406	1919	476 (431, 532)	3,116 (2,931, 3,336)	6.97 (6.93, 7.00)	0.92	81.91%
	**C11**	**5520**	5262	2349	595 (533, 673)	5,312 (4,910, 5,778)	7.20 (7.16, 7.23)	0.93	71.25%
	**C12**	**5520**	5412	1937	457 (409, 517)	3,688 (3,416, 4,009)	6.96 (6.93, 7.00)	0.92	80.34%
***Archaea***	**C1**	**82**	78	10	5 (4, 6)	12 (10, 26)	1.72 (1.53, 1.91)	0.75	94.87%
	**C2**	**82**	76	13	6 (4, 7)	16 (13, 30)	1.94 (1.72, 2.16)	0.76	93.42%
	**C3**	**82**	77	12	4 (3, 6)	14 (12, 24)	1.74 (1.48, 1.99)	0.70	94.81%
	**C4**	**82**	72	11	6 (5, 8)	14 (11, 34)	1.92 (1.73, 2.12)	0.80	94.44%
	**C5**	**82**	82	10	4 (3, 5)	15 (11, 42)	1.57 (1.36, 1.78)	0.68	93.90%
	**C6**	**82**	79	12	5 (4, 7)	15 (13, 34)	1.86 (1.65, 2.07)	0.75	93.67%
	**C7**	**82**	81	12	5 (4, 6)	23 (14, 66)	1.73 (1.51, 1.95)	0.70	91.36%
	**C8**	**82**	78	11	4 (4, 6)	12 (11, 19)	1.74 (1.52, 1.97)	0.73	96.15%
	**C9**	**82**	76	12	4 (3, 5)	40 (19, 117)	1.58 (1.32, 1.83)	0.64	89.47%
	**C10**	**82**	79	8	5 (4, 5)	10 (8, 23)	1.61 (1.46, 1.76)	0.77	96.20%
	**C11**	**82**	74	11	5 (4, 6)	13 (11, 27)	1.78 (1.55, 2.00)	0.74	94.59%
	**C12**	**82**	81	9	5 (4, 6)	11 (9, 24)	1.68 (1.50, 1.86)	0.76	96.30%
***Fungi***	**C1**	**1046**	1015	27	4 (4, 4)	32 (28, 50)	1.76 (1.68, 1.84)	0.54	99.21%
	**C2**	**1046**	950	27	3 (2, 3)	33 (28, 54)	1.41 (1.32, 1.50)	0.43	99.16%
	**C3**	**1046**	1034	33	5 (5, 6)	39 (34, 60)	2.06 (1.98, 2.14)	0.59	99.13%
	**C4**	**1046**	1029	36	4 (4, 5)	56 (41, 109)	1.95 (1.86, 2.03)	0.54	98.74%
	**C5**	**1046**	1043	21	2 (2, 2)	21 (21, 0)	1.31 (1.22, 1.40)	0.43	99.90%
	**C6**	**1046**	1034	29	5 (5, 5)	29 (29, 32)	2.08 (2.01, 2.16)	0.62	99.81%
	**C7**	**1046**	1011	40	3 (3, 4)	80 (51, 181)	1.81 (1.71, 1.91)	0.49	98.42%
	**C8**	**1046**	1019	31	3 (3, 3)	37 (32, 58)	1.54 (1.46, 1.63)	0.45	99.12%
	**C9**	**1046**	1024	29	2 (2, 2)	34 (30, 52)	1.26 (1.17, 1.34)	0.37	99.22%
	**C10**	**1046**	1034	26	2 (2, 2)	30 (27, 48)	1.31 (1.22, 1.40)	0.40	99.32%
	**C11**	**1046**	1033	26	2 (2, 3)	31 (27, 54)	1.41 (1.32, 1.49)	0.43	99.32%
	**C12**	**1046**	1042	22	2 (2, 3)	25 (23, 44)	1.18 (1.10, 1.26)	0.38	99.52%

### Per-animal Archaeal Species Richness

An analysis of archaeal diversity was performed and summarized in **Table 2**, by clustering the sequences generated from PCR of the 16S rRNA gene using archaeal-specific primers A109F and A934R [29]. Anywhere between 8 and 13 species-level OTUs were found per animal at an indicated coverage of 89–96%. When counting the genus-level taxonomy of these OTUs, a total of 5 archaeal genera were observed with the majority of OTUs being composed of *Methanobrevibacter* and *Methanosphaera* species. *Thermogymnomonas* species were observed in 4 of the 12 animals (**Figure 2B**).

#### Per-animal fungal species richness

An OTU analysis analogous to those performed on bacterial and archaeal 16S rRNA gene sequences was performed on fungal 18S rRNA gene sequence reads and summarized in **Table 2**. Briefly, only between 21 and 40 OTUs were identified despite using about a 1,000 sequences per animal. The Good’s coverage [28] estimates of 98.4–99.9% indicated that nearly the full extent of fungal diversity in the rumen of 12 animals using primers EF4a and fung5a was captured. In the top 10 genera (**Figure 2C**), 5 were potentially novel, marked as unclassified at some taxonomic level. Among the known fungal genera, *Nectria*, *Penicilliopsis*, *Cystofilobasidium* and *Delphinella* were the most abundant, comprising over 25% of the 46 fungal genera detected (**Figure 2C**).

### Rumen Solid Versus Liquid Phase Species Richness

**Figure 3:**
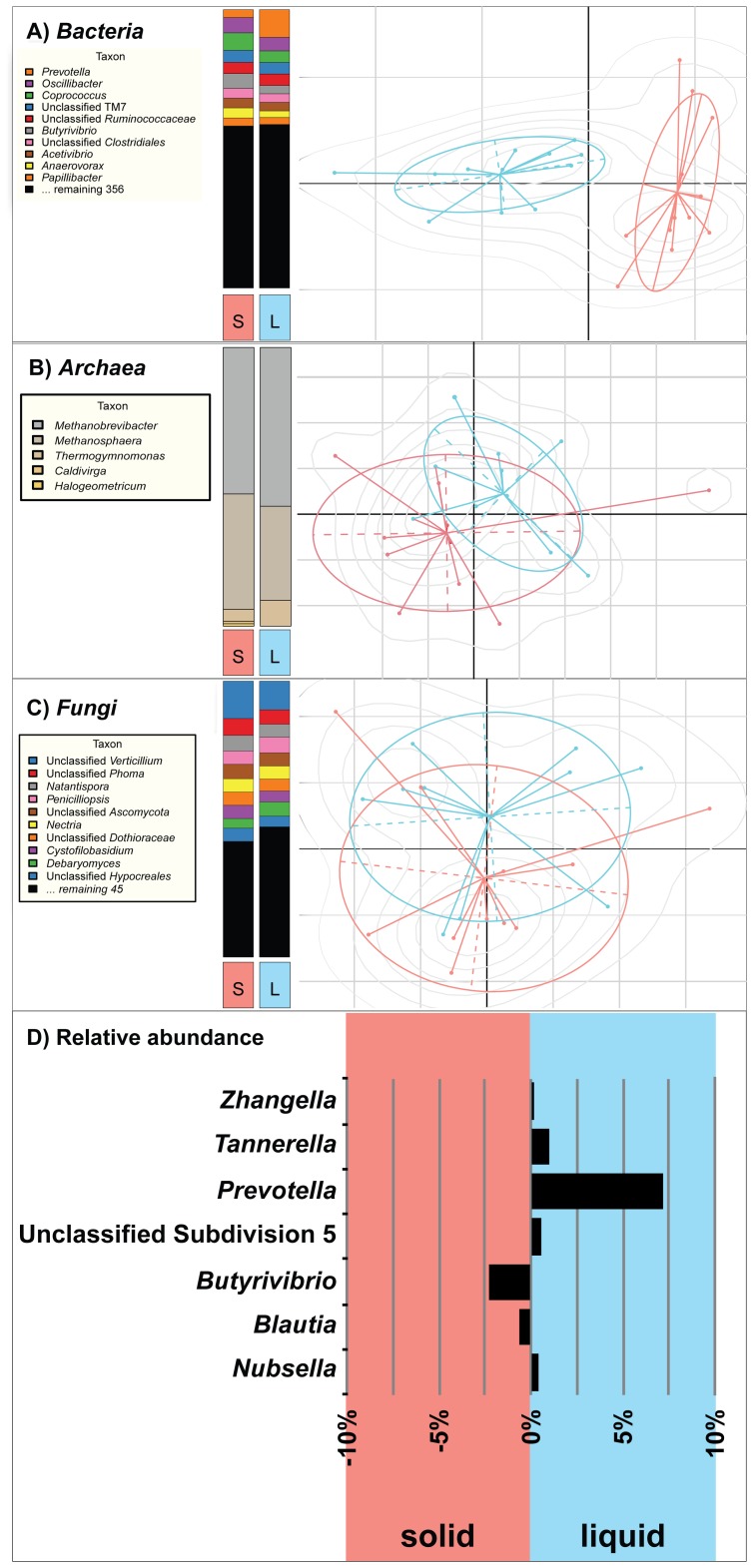
Comparison of microbial diversity in bovine rumen solid (S) and liquid (L) fractions in 12 cows. Bar-charts (left panels) and PCA scatter plots (right panels) display the OTU-based taxonomic profiles and similarity among profiles, respectively for *Bacteria* (A), *Archaea* (B), and *Fungi* (C). The relative abundance of seven bacteria with significant differences in liquid and solid fractions of the rumen was plotted (D).

Samples of ruminal content from all 12 animals were separated into solid and liquid fractions (Text S1). To determine if observed differences in bacterial OTUs between liquid and solid fractions as measured by taxonomic profiling and PCA were statistically significant, the Wilcoxon non-parametric t-test corrected for multiple hypothesis testing [29] was implemented. Bacterial biodiversity of seven genera differed significantly (P<0.05) between liquid and solid fractions of the rumen contents (**Figure 3A** **, Table S2**), while there was no statistical difference observed between the liquid and solid fractions in biodiversity of *Archaea* (**Figure 3B**), and *Fungi* (**Figure 3C** **)**. *Prevotella* and *Tannerella* (both members of the order *Bacteroidales*) were overrepresented in the liquid fraction of the rumen (**Figure 3D** **)**. Conversely, *Butyrivibrio* and *Blautia* (both members of the order *Clostridiales*) were significantly overrepresented in the solid fraction of the rumen. These results are consistent with previous observations that *Prevotella* are more prevalent in the liquid fraction of pasture-fed cows [11] and bermudagrass hay- or wheat-fed steers [12]. Likewise, *Butyrivibrio*, a member of the family *Lachnospiraceae*, was also shown to be more abundant in the solid fraction of pasture-fed cows [11] and bermudagrass hay- or wheat-fed steers [12]. However, the *Tannerella* and *Blautia* (a.k.a. *Ruminococcus*) results vary more across these studies. This may be due to differences in geographical location, diet, time of sampling post feeding, and the genetic background or sex of the animals.

### Cross-domain Analysis of the Microbiome

**Figure 4:**
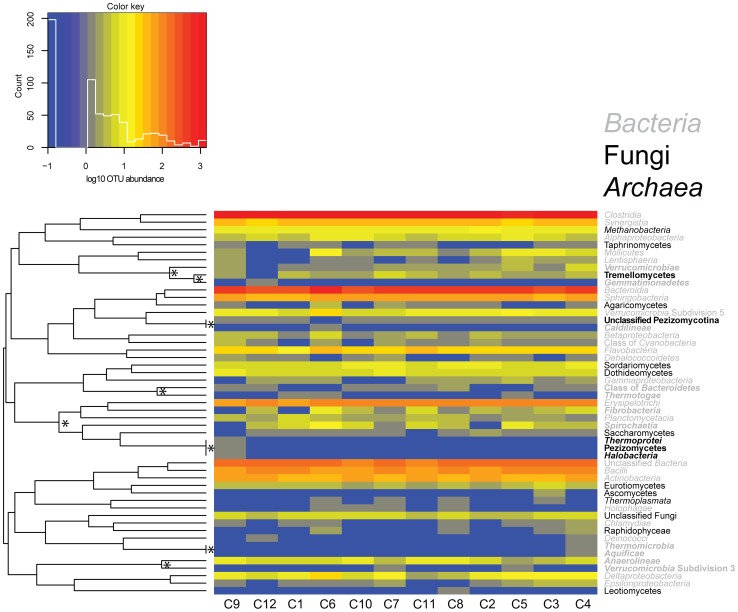
Cross domain OTU comparison based on abundance pattern correlations. Taxonomic classes of *Bacteria* (gray italics), Fungi (black) and *Archaea* (black italics) are listed on the right side of the plot. The key denotes log10 transformed abundance patterns. The taxa are clustered based on abundance pattern correlation using a distance metric defined as 1-|r|. The dendrogram on the left side of the plot summarizes the clustering of taxa. Asterisks on nodes denote statistically significant correlations (P<0.05). The names of significantly significant correlated classes are indicated by bold face font.

Little is known regarding cross-domain interactions among the inhabitants of the bovine rumen. To address this void, a comprehensive analysis of the patterns of abundance of *Bacteria*, *Archaea* and *Fungi* were determined across the 12 animals. Based on comparison of the relative abundance of OTUs in different domains, especially in the case of *Bacteria* and *Archaea* whose abundances differed by several orders of magnitude, a log transformation was applied to the raw OTU counts. These log-transformed abundances observed across 12 cows were then hierarchically clustered based on distance calculated as 1– |r| (where r is the linear correlation coefficient) of any combination of two microbial taxa and represented as a heatmap (**Figure 4** **).** To determine statistical significance of observed correlations, fdrtool [30], [31] was used to calculate false discovery rate-corrected (FDR) q-values for each correlation coefficient. In all, 74691 pairwise combinations of genera were analyzed to produce 1424 significant (qval <0.05) correlations (**Table S3**), too many to interpret manually. However, on a taxonomic level of class, 1275 possible correlations were calculated and 10 were significant (qval <0.05) (**Table S4,** and noted with asterisks and boldface in **Figure 4** **)**. Notably, abundance of an unclassified fungal class of subphylum *Pezizomycotina* was inversely correlated with *Caldilineae* and *Verrucomicrobia* Subdivision 5 Bacteria (RDP taxonomy from MOTHUR). The Subdivision 5 is a class of uncultured *Verrucomicrobia* that was first identified from a hydrocarbon-contaminated aquifer [32]. Abundance of the fungal class *Tremellomycetes* was positively correlated with abundance of bacterial class *Verrucomicrobiae* and negatively correlated with abundance of bacterial class of *Gemmatimonadetes*. On the other hand, abundance of one member of *Pezizomycetes* (fungal class) was positively correlated with abundance of *Halobacteria* and *Thermoprotei* (two members of the *Archaea*). Both *Halobacteria* and *Thermoprotei* were observed in only one animal (C9, Figure 2B) as *Halogeometricum* and *Caldivirga*, respectively. Future studies are needed to verify these cross-domain correlations and to provide a biological explanation for them (e.g., which community members are potentially metabolically interchangeable).

### Novel Fungal Taxa

**Figure 5:**
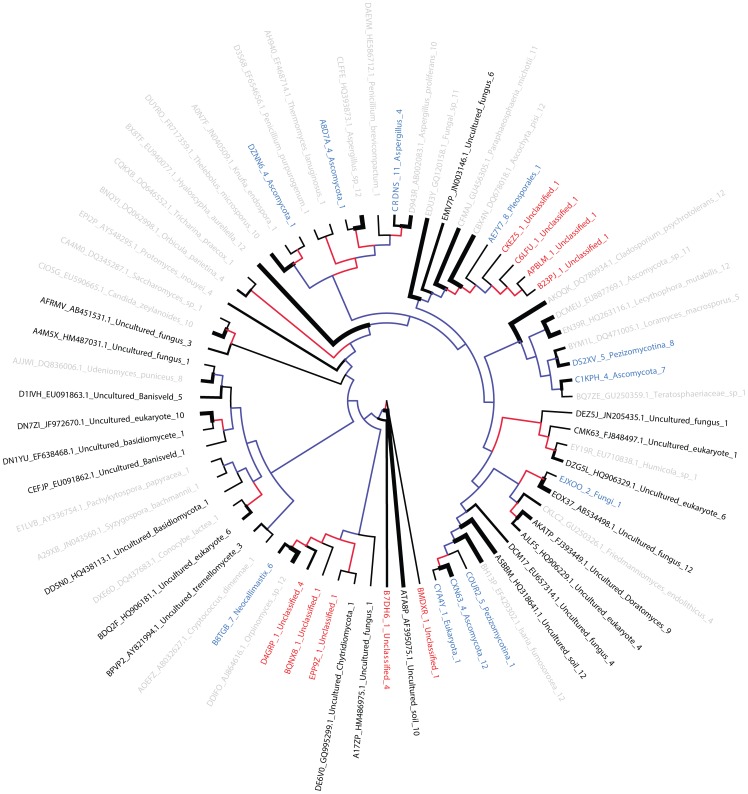
Phylogenetic diversity of fungal 18S rRNA sequences in bovine rumen. NJ tree clustering of OTU representatives labeled based on similarity to known sequences. The color of the branch indicates bootstrapping value: blue when bootstrapping was <50%, red when bootstrapping was >50%. The width of the branch is proportional to the number of animals (1–12) that exhibited a given OTU. This number of animals is also indicated in the last part of the OTU label. Gray- and black-labeled OTUs were classified using BLAST and at least 97% identity to a target sequence. Black labels highlight a subset of these OTUs with vague annotation. OTUs labeled in blue did not match a target using BLAST, but were subsequently classified using LCA based on 90% identity to a SILVA database match. OTUs in red failed both BLAST and LCA and are presumably novel taxa.

To investigate the striking disparity between the fungal sequences identified in the current study and the sequences currently available in the public repositories (**Figure 1C**), a phylogenetic tree was inferred, illustrating taxonomic relationships among the representative OTU sequences (**Figure 5**). Of the 71 total fungal OTUs identified in this study, only 53 grouped near a previously deposited sequence (gray and black leaves, **Figure 5**). The most abundant OTU identified in this study represented by over 4620 sequences and present in all 12 animals with sequence similarity to *Aschochyta pisi*, a known fungal pathogen responsible for blight of common crops such as peas. The second most abundant fungal OTU identified in this study resembled a recently characterized species of *Aspergillus* (PSBORB-4, Genbank accession HQ393873.1, unpublished). This OTU was represented by 4612 sequences and was also identified in all 12 animals. *Aspergillus* isolate PSBORB-4 clustered with *Aspergillus proliferans*; however, based on read counts, PSBORB-4 was over 220 times more abundant. Furthermore, two additional OTUs that were classified as “Ascomycota” and “*Aspergillus*” clustered with PSBORB-4 and *A. proliferans*, alluding to potentially novel *Aspergillus* species that are yet to be discovered.

Close to 40% (black leaves in **Figure 5**) of the 53 BLAST-matched OTUs were rudimentarily annotated in the public repository (e.g., “uncultured soil fungus”, and without any definite taxonomic classification). The remaining 20 OTU-representative sequences did not match any known targets in the recent release of the nt database at NCBI (red and blue leaves in **Figure 5**). For these 20 sequences, least common ancestor (LCA) analysis revealed a possible taxonomic placement for 11 sequences (indicated as blue leaves in **Figure 5**) leaving 9 sequences as unclassifiable, (red leaves in **Figure 5**), representing presumably novel taxa of the bovine rumen. Our analysis showed that the current landscape of the fungal diversity in the rumen is largely incomplete. Specifically, that there is a greater than previously appreciated diversity of *Pleosporales*, *Neocallimastix*, *Sordariomyceteideae*, *Udeniomyces* and others.

### Conclusion

Sequencing of the SSU rRNA gene of *Bacteria* and *Fungi* from bovine rumen suggests that the compositional characterization of the rumen microbiome is incomplete with several novel fungal taxa being discovered despite targeting the less specific 18S rRNA gene. In contrast, a comparison of archaeal SSU rRNA sequences with sequences from three public repositories resulted in only 2 new species. Bacterial community profiles differed between liquid and solid (fiber) fractions while the archaeal and fungal communities appeared indifferent. Integration of prokaryotic and fungal data sets highlighted the cross-domain correlations among the abundances of rumen inhabitants. Future studies should focus on exploring these dependences further via metagenomic and functional analysis of the bovine rumen.

## Materials and Methods

### Animals

Animals used in the study were cared for according to the guidelines of the USDA-ARS Fort Keogh Livestock and Range Research Laboratory (LARRL) Institutional Animal Care and Use Committee (IACUC) under approval number 21308-1. For this series of experiments, rumen samples were obtained from 27 month old crossbred *Bos taurus* (>75% Black Angus with the remainder being Hereford, Red Angus, Charolais, and Tarentaise) ruminally-cannulated multiparous beef females designated Cows 1–12. Cows were adapted to their diet and environment for at least 14 days before sample collection.

### Sample Preparation

Rumen samples from 12 different animals were obtained from ruminally-cannulated animals consuming harvested forage (Text S1). Rumen contents were mixed by hand before an aliquot was removed from the rumen. The liquid fraction of each animal was separated from the solid contents by filtration through sterile 90-grade cheesecloth. All samples were transported on dry ice to the J. Craig Venter Institute (JCVI) in Rockville, MD for DNA extraction, amplification and analysis (Text S1). SSU rRNA genes representing the diversity of *Bacteria*, *Archaea*, and *Fungi* were amplified for sequencing. Samples from the solid and liquid phases were handled separately through sequence completion.

### PCR Primers

Bacterial diversity was established by sequencing PCR amplicons generated using primers 27F [AGAGTTTGATYMTGGCTCAG] [33] and 534R [ATTACCGCGGCTGCTGG] [34], which target the highly variable V1–V3 region of the 16S gene [34] (Text S1). Fungal-specific primers for 454 pyrosequencing were used to PCR amplify an approximately 500 bp region of the 18S rRNA gene using primers EF4a [GGAAGGGRTGTATTTATTAG] and fung5a [GTAAAAGTCCTGGTTCCCC] [35]. Amplicons were then column purified (Qiaquick, Qiagen), quantified (Tecan Group Ltd.), and normalized in preparation for emPCR and 454 pyrosequencing. The archaeal 16S rRNA gene was amplified for Sanger sequencing using the following primer pairs: A109F [ACKGCTCAGTAACACGT] and A934R [GTGCTCCCCCGCCAATTCCT] [29] and column purified as above.

### DNA Sequence Processing

A rigorous sequence-processing pipeline was adapted that utilized LUCY [20], [21] and sequence base quality information to trim each read, remove low quality and short (<100 bp) reads. The subsequent quality control (QC) steps collapse sequencer-induced PCR duplicates using MOTHUR v1.22.2 [24] and further filter out sequence fragments using CD-HIT-454 [36]. While MOTHUR is capable of removing sequence fragments, the CD-HIT suite of tools [26] was found to be orders of magnitude faster with more modest hardware requirements, facilitating rapid, high-throughput analysis with comparable results to those of MOTHUR (data not shown). Subsequently, the remaining filtered reads were aligned against a SILVA database of 16S or 18S sequences [24], [37] to verify that the reads were indeed 16S or 18S and to determine that they map to the correct region of the respective rRNA gene. Subsequently, the pipeline utilized MOTHUR’s implementation of chimera slayer [24], [38] to filter out potentially chimeric reads. The entire rRNA sequence-processing pipeline is freely available as a part of the YAP package [39]. The processed 16S rDNA data from this study can be obtained at NCBI under BioProject ID PRJNA173217.

### OTU-based Sequence Analysis

A module of the CD-HIT suite [26], called CD-HIT-EST, was used to perform the read-clustering. The workflow engine that manages the succession of steps and their dispatch to grid nodes was implemented in python via YAP. An identity threshold of 97% was used to identify OTUs at approximately the species level [40].

### Taxonomic Classification of OTU Representative Reads

Taxonomic classification of the final set of representative reads was performed using MOTHUR’s version of the RDP Bayesian classifier [24], using a RDP training dataset number 6 [41] normalized to contain 6 taxonomic levels for each sequence. A similar approach was used for the 18S sequences, except they were classified using ARB [42] and BLAST [43] against the current NCBI nt database.

### Sequences Obtained from Public Repositories

Three repositories (SILVA [37], [44], RDP [45] and NCBI [46]) were queried to identify the bacterial and archaeal ruminal 16S sequences, and two repositories (SILVA and NCBI) were queried to identify all eukaryotic ruminal 18S sequences. Search terms included “organism domain” and “rRNA” where appropriate, “rumen,” “rumenal,” or “ruminal.” The sequences were then aligned to their respective set of 16S or 18S SILVA references using the MOTHUR aligner. The alignment was then trimmed based on the coordinates of the alignment so that only the sequences that could potentially be amplified using the primer sets used in this study were kept for subsequent analyses. (**Figures S1, S2, S3, and-S4, Table S1**).

### Phylogenetic Tree Building and Annotation

SSU rRNA gene sequences were aligned using SINA [47], a sequence alignment tool that considers primary and secondary sequence attributes incorporated into the SILVA rRNA reference compiled using ARB. Based on the alignment, a bootstrapped Neighbor-Joining (NJ) tree was subsequently inferred using paupFasta, an in-house wrapper script around the PAUP* program as described [48], [49], and edited using FigTree [50]. The annotation of tree leaves was performed using TimeLogic™ TERA-BLAST of OTU representative sequences against the November 2011 release of the NCBI nt database. For each of the sequences, one representative database hit was kept if fewer than 3 mismatches per 100 nucleotides were observed between it and the query. For all of the sequences that did not have a database match with fewer than 3 mismatches per 100 bases, a least common ancestor (LCA) analysis with a threshold parameter 0.9 was performed using SILVA taxonomy at the SILVA web site [44].

### Cross-domain Correlations

The analysis of cross-domain correlations was performed using a script written in the R statistical computing language [51]. All OTU abundances were converted to log base 10, using a standard R function, *log10*. All non-redundant combinations of any two genera were listed, and the correlations of OTU abundances across the 12 cows were calculated. A function, *cor*, part of the default installation of R, was used to calculate the Pearson correlation coefficients (r) of every listed combination. The R package fdrtool [30], [31] was used to correct for multiple hypothesis testing and to generate q values for each correlation. In addition to the pair-wise analysis, a hierarchical clustering of genera, based on their abundance patterns across 12 cows, was performed. To group multiple genera together based on the OTU abundance patterns, the *heatmap.2* function, a part of the gplots R package, available via CRAN, was used. The distance function supplied to the *heatmap.2* function as an argument was defined as “1– |r|” of the OTU abundances between any two genera across 12 cows. The standard *hclust* R function with an argument “average”, for average linkage clustering, was supplied to the *heatmap.2* function to compare the distances between any two genera and to produce the dendrogram visible in the heatmap figure.

## Supporting Information

Figure S1 Bacterial sequence alignments with SILVA and *E. coli* coordinates. Rumen bacterial sequences in public repositories (A) or from this study (B) were aligned to the SILVA bacterial 16S rRNA reference alignment. Coordinates to the SILVA alignment are above the plot, while *E. coli* coordinates are below the plot.
(PDF)

Figure S2 Archaeal sequence alignments with SILVA and *E. coli* coordinates. Rumen archaeal sequences in public repositories (A) or from this study (B) were aligned to the SILVA archaeal 16S rRNA reference alignment. Coordinates to the SILVA alignment are above the plot, while *E. coli* coordinates are below the plot.
(PDF)

Figure S3 Eukaryotic sequence alignments with SILVA and *S. cerevisiae* coordinates. Rumen eukaryotic sequences in public repositories (A) or from this study (B) were aligned to the SILVA 18S rRNA reference alignment. Coordinates to the SILVA alignment are above the plot, while *S. cerevisiae* coordinates are below the plot.
(PDF)

Figure S4 Comparison of rumen sequences obtained from public repositories. The Venn diagram shows the number of bacterial (A), archaeal (B) and eukaryotic (C) OTUs shared within a particular relationship for all three public repositories compared.
(PDF)

Table S1 **Summary of diversity stored in public repositories.**
(PDF)

Table S2 **Bacterial genera significantly differentiating liquid and solid fractions among 12 cows.**
(PDF)

Table S3 **Correlations among bacterial, archaeal, and fungal genera.**
(PDF)

Table S4 **Statistically significant cross-domain pairwise correlations between bacterial, archaeal and fungal classes.**
(PDF)

Text S1 Supplemental methods describing animal breed, animal care, sample harvesting and processing, DNA purification, PCR conditions, and sequencing.
(DOCX)
