# Supplementary material for: Next Generation Sequencing to Define Prokaryotic and Fungal Diversity in the Bovine Rumen
Source: PLoS One. 2012 Nov 7;7(11):e48289. doi: 10.1371/journal.pone.0048289 (PMC3492333; doi:10.1371/journal.pone.0048289)

Figure S2. Archaeal sequence alignments with SILVA and *E. coli* coordinates.

A) Rumen archaeal sequences in public repositories aligned to SILVA.

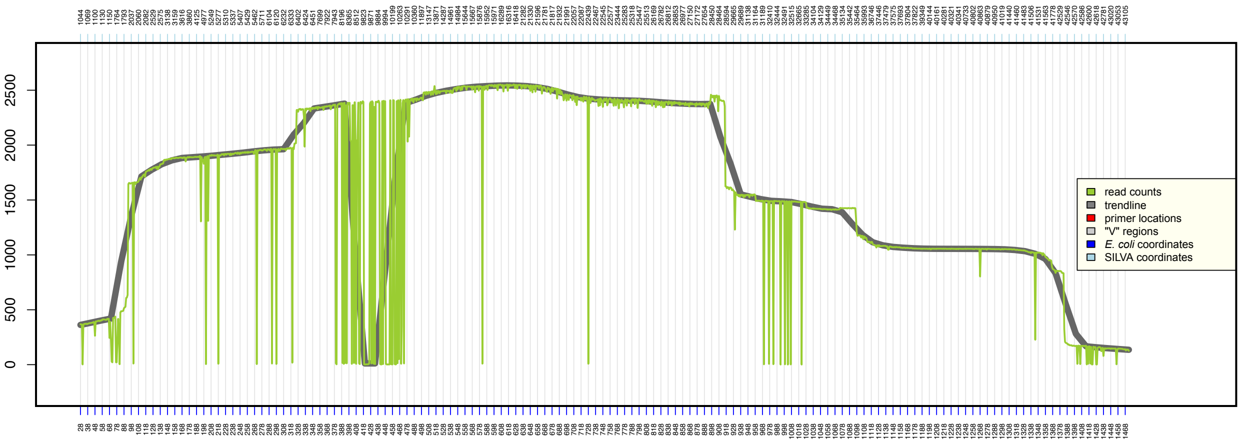

B) Archaeal reads from this study aligned to SILVA

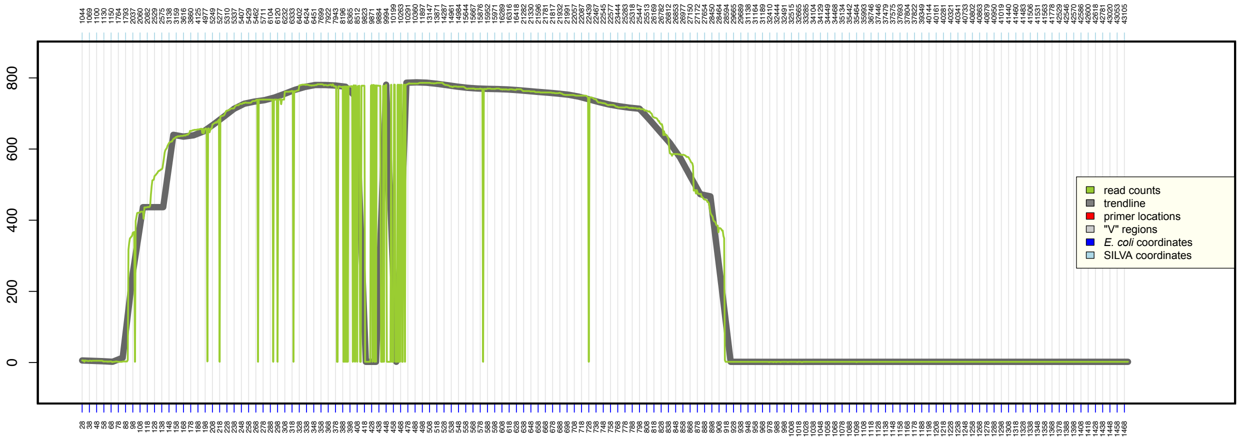

Supplement: Figure S2 — Archaeal sequence alignments with SILVA and E. coli coordinates. Rumen archaeal sequences in public repositories (A) or from this study (B) were aligned to the SILVA archaeal 16S rRNA reference alignment. Coordinates to the SILVA alignment are above the plot, while E. coli coordinates are below the plot. (PDF) [file pone.0048289.s002.pdf]
