# Supplementary material for: Next Generation Sequencing to Define Prokaryotic and Fungal Diversity in the Bovine Rumen
Source: PLoS One. 2012 Nov 7;7(11):e48289. doi: 10.1371/journal.pone.0048289 (PMC3492333; doi:10.1371/journal.pone.0048289)

Figure S3. Eukaryotic sequence alignments with SILVA and *S. cerevisiae* coordinates.

A) Rumen eukaryotic sequences in public repositories aligned to SILVA.

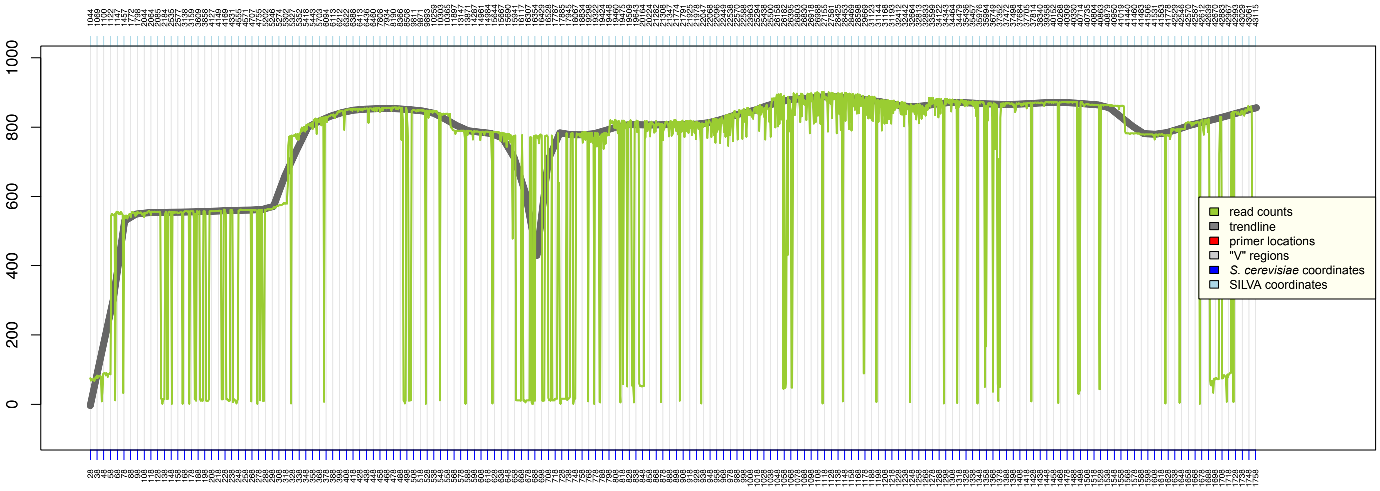

B) Fungal reads from this study aligned to SILVA

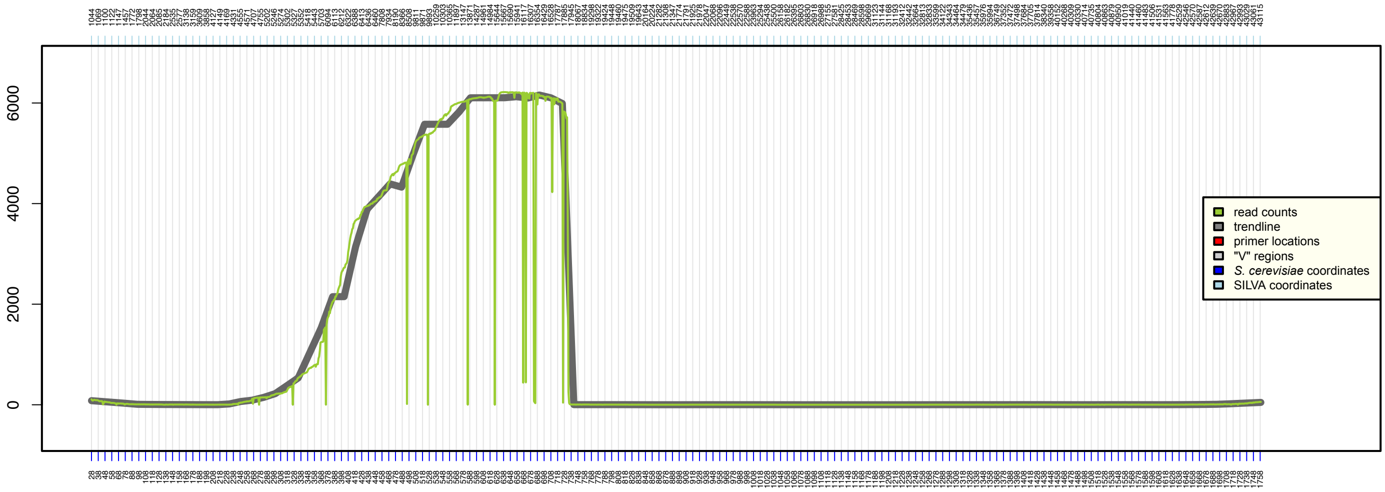

Supplement: Figure S3 — Eukaryotic sequence alignments with SILVA and S. cerevisiae coordinates. Rumen eukaryotic sequences in public repositories (A) or from this study (B) were aligned to the SILVA 18S rRNA reference alignment. Coordinates to the SILVA alignment are above the plot, while S. cerevisiae coordinates are below the plot. (PDF) [file pone.0048289.s003.pdf]
