# Supplementary figures and images for: Next Generation Sequencing to Define Prokaryotic and Fungal Diversity in the Bovine Rumen
Source: PLoS One. 2012 Nov 7;7(11):e48289. doi: 10.1371/journal.pone.0048289 (PMC3492333; doi:10.1371/journal.pone.0048289)

Figure S4. Comparison of rumen sequences obtained from public repositories.

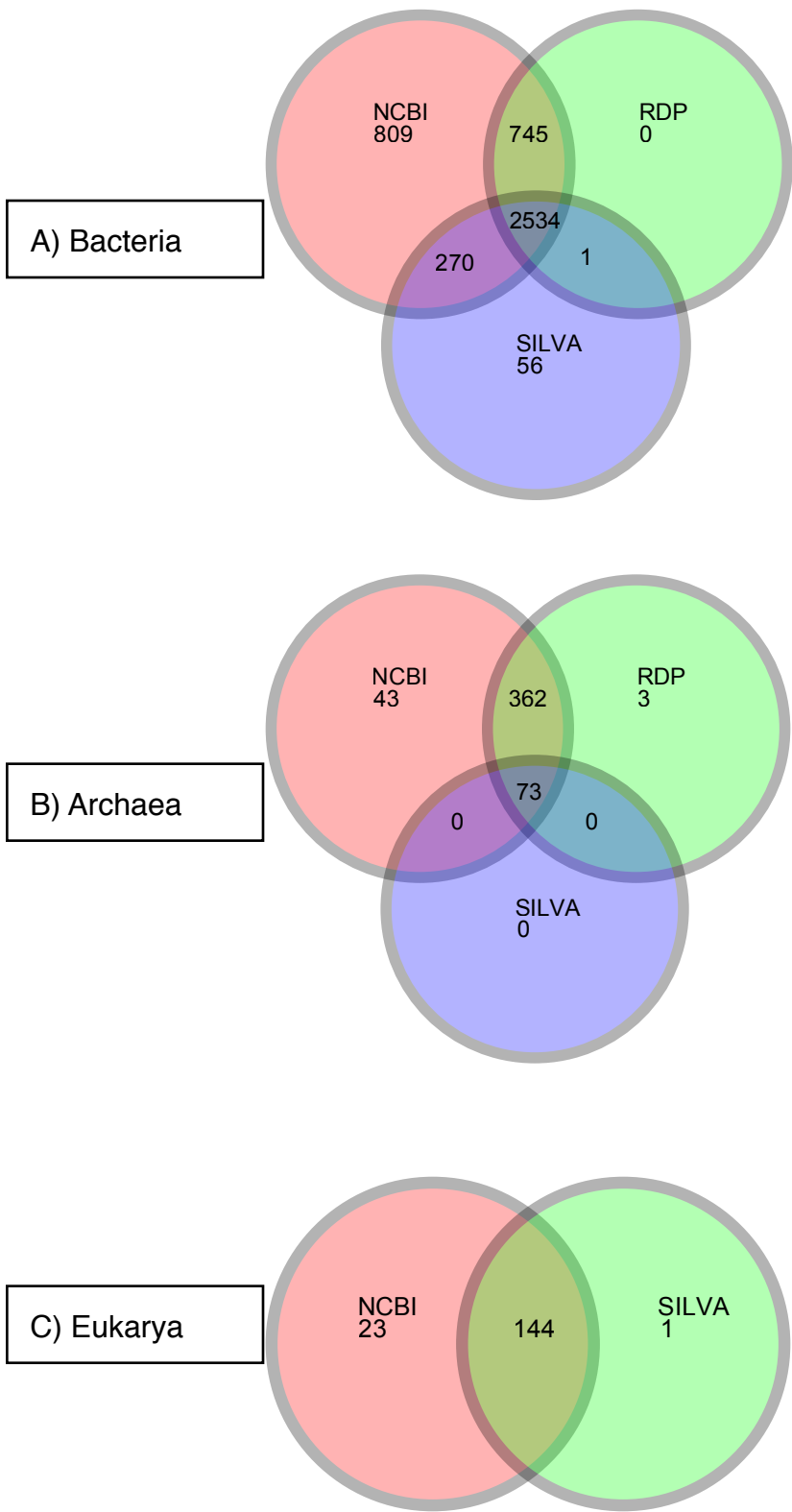

Supplement: Figure S4 — Comparison of rumen sequences obtained from public repositories. The Venn diagram shows the number of bacterial (A), archaeal (B) and eukaryotic (C) OTUs shared within a particular relationship for all three public repositories compared. (PDF) [file pone.0048289.s004.pdf]
