# Supplementary material for: Next Generation Sequencing to Define Prokaryotic and Fungal Diversity in the Bovine Rumen
Source: PLoS One. 2012 Nov 7;7(11):e48289. doi: 10.1371/journal.pone.0048289 (PMC3492333; doi:10.1371/journal.pone.0048289)
Supplement: Table S1 — Summary of diversity stored in public repositories. (PDF) [file pone.0048289.s005.pdf]

TableS1

	Label	# public sequences retrieved	# unique & trimmed sequences	# of OTUs	Inv Simpson	Chao
Bacteria	REF_NCBI	22485	16046	4358	763 (727, 802)	8,176 (7,763, 8,641)
	REF_RDP	15637	10579	3280	573 (539, 611)	6,518 (6,123, 6,966)
	REF_SILVA	12153	9044	2861	390 (365, 418)	6,063 (5,651, 6,536)
Archaea	REF_NCBI	4198	3078	478	10 (9, 11)	1,985 (1,521, 2,655)
	REF_RDP	3703	2783	438	9 (8, 10)	1,722 (1,322, 2,303)
	REF_SILVA	1120	938	73	8 (7, 9)	151 (106, 260)
Eukarya	REF_NCBI	1803	984	167	10 (9, 11)	782 (499, 1,308)
	REF_SILVA	1027	764	145	9 (8, 10)	801 (478, 1,435)

Shannon	Shannon Evenness	Coverage
7.54 (7.51, 7.56)	0.90	85.63%
7.29 (7.27, 7.32)	0.90	82.62%
7.06 (7.03, 7.10)	0.89	81.39%
3.80 (3.72, 3.88)	0.62	88.56%
3.67 (3.58, 3.75)	0.60	88.32%
2.76 (2.66, 2.85)	0.64	95.95%
3.27 (3.16, 3.39)	0.64	87.09%
3.13 (3.00, 3.27)	0.63	84.95%
