# Supplementary material for: Next Generation Sequencing to Define Prokaryotic and Fungal Diversity in the Bovine Rumen
Source: PLoS One. 2012 Nov 7;7(11):e48289. doi: 10.1371/journal.pone.0048289 (PMC3492333; doi:10.1371/journal.pone.0048289)
Supplement: Table S2 — Bacterial genera significantly differentiating liquid and solid fractions among 12 cows. (PDF) [file pone.0048289.s006.pdf]

Table S2

		Bolus		Liquid		
label	Wilcoxon	Average	SD	Average	SD	liquid - bolus
Zhangella	2.45E-05	0.01%	0.03%	0.18%	0.04%	0.17%
Tannerella	3.66E-05	0.74%	0.26%	1.73%	0.27%	1.00%
Prevotella	3.66E-05	2.73%	1.18%	9.92%	3.39%	7.18%
Unclassified Subdivision 5	5.57E-05	0.12%	0.12%	0.69%	0.36%	0.57%
Butyrivibrio	6.01E-05	5.26%	1.02%	2.98%	0.53%	-2.27%
Blautia	8.61E-05	1.07%	0.26%	0.45%	0.24%	-0.62%
Nubsella	0.0001	0.09%	0.11%	0.51%	0.28%	0.42%
