# Supplementary material for: Next Generation Sequencing to Define Prokaryotic and Fungal Diversity in the Bovine Rumen
Source: PLoS One. 2012 Nov 7;7(11):e48289. doi: 10.1371/journal.pone.0048289 (PMC3492333; doi:10.1371/journal.pone.0048289)
Supplement: Table S3 — Correlations among bacterial, archaeal, and fungal genera. (PDF) [file pone.0048289.s007.pdf]

Table S3

a	b	r	pval	qval	lfdr
Sediminicola_B	Abiotrophia_B	9.91E-01	1.88E-10	1.85E-08	5.47E-06
Syntrophothermus_B	Abiotrophia_B	1.00E+00	2.22E-16	2.21E-14	1.90E-06
Carnobacterium_B	Acetanaerobacterium_B	8.52E-01	3.27E-04	1.87E-02	2.17E-01
Malonomonas_B	Acetanaerobacterium_B	-8.29E-01	6.66E-04	3.50E-02	2.17E-01
Proteocatella_B	Acetanaerobacterium_B	8.96E-01	5.69E-05	3.85E-03	6.01E-02
Atopobium_B	Acetitomaculum_B	8.55E-01	2.97E-04	1.72E-02	2.17E-01
Brevibacterium_B	Acetitomaculum_B	-9.66E-01	1.81E-07	1.69E-05	2.21E-03
Desulfonatronospira_B	Acetitomaculum_B	9.59E-01	4.70E-07	4.32E-05	2.21E-03
Faecalibacterium_B	Acetivibrio_B	8.84E-01	9.51E-05	6.16E-03	6.01E-02
Hydrogenoanaerobacterium_B	Acetivibrio_B	9.03E-01	3.98E-05	2.76E-03	4.17E-02
Pseudozobellia_B	Acetivibrio_B	8.18E-01	8.87E-04	4.47E-02	2.68E-01
Alkalibacterium_B	Acetohalobium_B	-9.18E-01	1.66E-05	1.24E-03	7.84E-03
Bimuria_F	Acetohalobium_B	-9.18E-01	1.66E-05	1.24E-03	7.84E-03
Brachymonas_B	Acetohalobium_B	-9.18E-01	1.66E-05	1.24E-03	7.84E-03
Bulleidia_B	Acetohalobium_B	-8.74E-01	1.45E-04	8.92E-03	7.38E-02
Desemzia_B	Acetohalobium_B	-9.18E-01	1.66E-05	1.24E-03	7.84E-03
Desulfatiferula_B	Acetohalobium_B	-9.18E-01	1.66E-05	1.24E-03	7.84E-03
Holtermannia_F	Acetohalobium_B	-9.18E-01	1.66E-05	1.24E-03	7.84E-03
Leuconostoc_B	Acetohalobium_B	-9.18E-01	1.66E-05	1.24E-03	7.84E-03
Levilinea_B	Acetohalobium_B	8.71E-01	1.63E-04	9.92E-03	1.04E-01
Parasporobacterium_B	Acetohalobium_B	-8.85E-01	9.33E-05	6.06E-03	6.01E-02
Phascolarctobacterium_B	Acetohalobium_B	-9.18E-01	1.66E-05	1.24E-03	7.84E-03
Pirellula_B	Acetohalobium_B	-9.18E-01	1.66E-05	1.24E-03	7.84E-03
Rudanella_B	Acetohalobium_B	-9.18E-01	1.66E-05	1.24E-03	7.84E-03
Thermonema_B	Acetohalobium_B	-9.18E-01	1.66E-05	1.24E-03	7.84E-03
Tropheryma_B	Acetohalobium_B	-9.18E-01	1.66E-05	1.24E-03	7.84E-03
Unc_Cryptococcus_F	Acetohalobium_B	-9.18E-01	1.66E-05	1.24E-03	7.84E-03
Verminephrobacter_B	Acetohalobium_B	-9.18E-01	1.66E-05	1.24E-03	7.84E-03

Coenonia_B	Acholeplasma_B	1.00E+00	2.22E-16	2.21E-14	1.90E-06
Fulvimonas_B	Acholeplasma_B	1.00E+00	2.22E-16	2.21E-14	1.90E-06
Fulvivirga_B	Acholeplasma_B	1.00E+00	2.22E-16	2.21E-14	1.90E-06
Jonesia_B	Acholeplasma_B	1.00E+00	2.22E-16	2.21E-14	1.90E-06
Lishizhenia_B	Acholeplasma_B	1.00E+00	2.22E-16	2.21E-14	1.90E-06
Ornithobacterium_B	Acholeplasma_B	-9.11E-01	2.50E-05	1.78E-03	3.13E-02
Parachlamydia_B	Acholeplasma_B	1.00E+00	2.22E-16	2.21E-14	1.90E-06
Sphaerobacter_B	Acholeplasma_B	1.00E+00	2.22E-16	2.21E-14	1.90E-06
Sporanaerobacter_B	Acholeplasma_B	1.00E+00	2.22E-16	2.21E-14	1.90E-06
Unc_Kockovaella_F	Acholeplasma_B	1.00E+00	2.22E-16	2.21E-14	1.90E-06
Venenivibrio_B	Acholeplasma_B	1.00E+00	2.22E-16	2.21E-14	1.90E-06
Zunongwangia_B	Acholeplasma_B	1.00E+00	2.22E-16	2.21E-14	1.90E-06
Longilinea_B	Acidaminobacter_B	-8.51E-01	3.41E-04	1.95E-02	2.17E-01
Isobaculum_B	Actinobaculum_B	-9.54E-01	8.47E-07	7.66E-05	2.21E-03
Schlesneria_B	Actinobaculum_B	1.00E+00	2.22E-16	2.21E-14	1.90E-06
Bdellovibrio_B	Adlercreutzia_B	-9.54E-01	8.47E-07	7.66E-05	2.21E-03
Kosmotoga_B	Adlercreutzia_B	-9.54E-01	8.47E-07	7.66E-05	2.21E-03
Thermovirga_B	Adlercreutzia_B	8.45E-01	4.04E-04	2.27E-02	2.17E-01
Alkaliphilus_B	Aeriscardovia_B	1.00E+00	2.22E-16	2.21E-14	1.90E-06
Coriobacterium_B	Aeriscardovia_B	1.00E+00	2.22E-16	2.21E-14	1.90E-06
Desulfotomaculum_B	Aeriscardovia_B	1.00E+00	2.22E-16	2.21E-14	1.90E-06
Ganoderma_F	Aeriscardovia_B	1.00E+00	2.22E-16	2.21E-14	1.90E-06
Geosmithia_putterillii_sensu_Pitt_F	Aeriscardovia_B	-9.16E-01	1.90E-05	1.39E-03	1.77E-02
Johnsonella_B	Aeriscardovia_B	1.00E+00	2.22E-16	2.21E-14	1.90E-06
Leptonema_B	Aeriscardovia_B	1.00E+00	2.22E-16	2.21E-14	1.90E-06
Lutimonas_B	Aeriscardovia_B	1.00E+00	2.22E-16	2.21E-14	1.90E-06
Massilia_B	Aeriscardovia_B	1.00E+00	2.22E-16	2.21E-14	1.90E-06
Parasegetibacter_B	Aeriscardovia_B	-9.42E-01	2.86E-06	2.46E-04	7.84E-03
Schwartzia_B	Aeriscardovia_B	-9.26E-01	9.83E-06	7.85E-04	7.84E-03
Tepidanaerobacter_B	Aeriscardovia_B	1.00E+00	2.22E-16	2.21E-14	1.90E-06

Thermoflavimicrobium_B	Aeriscardovia_B	1.00E+00	2.22E-16	2.21E-14	1.90E-06
Unc_Verticillium_F	Aeriscardovia_B	1.00E+00	2.22E-16	2.21E-14	1.90E-06
Guggenheimella_B	Aeromicrobium_B	-9.72E-01	6.40E-08	6.11E-06	2.87E-04
Lentisphaera_B	Aeromicrobium_B	1.00E+00	2.22E-16	2.21E-14	1.90E-06
Sedimentibacter_B	Aeromicrobium_B	-9.54E-01	8.47E-07	7.66E-05	2.21E-03
Xylanibacter_B	Aeromicrobium_B	-8.20E-01	8.40E-04	4.26E-02	2.68E-01
Alloscardovia_B	Akkermansia_B	1.00E+00	2.22E-16	2.21E-14	1.90E-06
Anaerofilum_B	Akkermansia_B	-9.21E-01	1.40E-05	1.07E-03	7.84E-03
Atopobacter_B	Akkermansia_B	1.00E+00	2.22E-16	2.21E-14	1.90E-06
Bellilinea_B	Akkermansia_B	-8.66E-01	1.97E-04	1.17E-02	1.04E-01
Blautia_B	Akkermansia_B	-8.83E-01	9.96E-05	6.43E-03	6.06E-02
Catonella_B	Akkermansia_B	-8.73E-01	1.53E-04	9.35E-03	1.04E-01
Clostridiisalibacter_B	Akkermansia_B	1.00E+00	2.22E-16	2.21E-14	1.90E-06
Desulforegula_B	Akkermansia_B	1.00E+00	2.22E-16	2.21E-14	1.90E-06
Enterorhabdus_B	Akkermansia_B	-9.17E-01	1.75E-05	1.30E-03	7.84E-03
Haloglycomyces_B	Akkermansia_B	1.00E+00	2.22E-16	2.21E-14	1.90E-06
Hespellia_B	Akkermansia_B	-9.70E-01	1.04E-07	9.77E-06	3.19E-04
Microscilla_B	Akkermansia_B	1.00E+00	2.22E-16	2.21E-14	1.90E-06
Petrimonas_B	Akkermansia_B	1.00E+00	2.22E-16	2.21E-14	1.90E-06
Porphyromonas_B	Akkermansia_B	1.00E+00	2.22E-16	2.21E-14	1.90E-06
Propionibacterium_B	Akkermansia_B	1.00E+00	2.22E-16	2.21E-14	1.90E-06
Pseudomonas_B	Akkermansia_B	1.00E+00	2.22E-16	2.21E-14	1.90E-06
Sanguibacter_B	Akkermansia_B	1.00E+00	2.22E-16	2.21E-14	1.90E-06
Shuttleworthia_B	Akkermansia_B	-8.88E-01	8.23E-05	5.41E-03	6.01E-02
Spirochaeta_B	Akkermansia_B	1.00E+00	2.22E-16	2.21E-14	1.90E-06
unclassified_F	Akkermansia_B	-8.44E-01	4.25E-04	2.37E-02	2.17E-01
Bavariicoccus_B	Alkalibacterium_B	-8.99E-01	4.85E-05	3.31E-03	4.24E-02
Bimuria_F	Alkalibacterium_B	1.00E+00	2.22E-16	2.21E-14	1.90E-06
Brachymonas_B	Alkalibacterium_B	1.00E+00	2.22E-16	2.21E-14	1.90E-06
Bulleidia_B	Alkalibacterium_B	8.79E-01	1.18E-04	7.48E-03	6.06E-02

Desemzia_B	Alkalibacterium_B	1.00E+00	2.22E-16	2.21E-14	1.90E-06
Desulfatiferula_B	Alkalibacterium_B	1.00E+00	2.22E-16	2.21E-14	1.90E-06
Holtermannia_F	Alkalibacterium_B	1.00E+00	2.22E-16	2.21E-14	1.90E-06
Leuconostoc_B	Alkalibacterium_B	1.00E+00	2.22E-16	2.21E-14	1.90E-06
Levilinea_B	Alkalibacterium_B	-9.17E-01	1.75E-05	1.30E-03	7.84E-03
Parasporobacterium_B	Alkalibacterium_B	8.63E-01	2.19E-04	1.29E-02	2.17E-01
Phascolarctobacterium_B	Alkalibacterium_B	1.00E+00	2.22E-16	2.21E-14	1.90E-06
Pirellula_B	Alkalibacterium_B	1.00E+00	2.22E-16	2.21E-14	1.90E-06
Rhodopirellula_B	Alkalibacterium_B	-8.76E-01	1.33E-04	8.28E-03	7.38E-02
Rudanella_B	Alkalibacterium_B	1.00E+00	2.22E-16	2.21E-14	1.90E-06
Thermonema_B	Alkalibacterium_B	1.00E+00	2.22E-16	2.21E-14	1.90E-06
Tropheryma_B	Alkalibacterium_B	1.00E+00	2.22E-16	2.21E-14	1.90E-06
Unc_Cryptococcus_F	Alkalibacterium_B	1.00E+00	2.22E-16	2.21E-14	1.90E-06
Verminephrobacter_B	Alkalibacterium_B	1.00E+00	2.22E-16	2.21E-14	1.90E-06
Desulfonatronospira_B	Alkaliflexus_B	8.54E-01	3.01E-04	1.74E-02	2.17E-01
Coriobacterium_B	Alkaliphilus_B	1.00E+00	2.22E-16	2.21E-14	1.90E-06
Desulfotomaculum_B	Alkaliphilus_B	1.00E+00	2.22E-16	2.21E-14	1.90E-06
Ganoderma_F	Alkaliphilus_B	1.00E+00	2.22E-16	2.21E-14	1.90E-06
Geosmithia_putterillii_sensu_Pitt_F	Alkaliphilus_B	-9.16E-01	1.90E-05	1.39E-03	1.77E-02
Johnsonella_B	Alkaliphilus_B	1.00E+00	2.22E-16	2.21E-14	1.90E-06
Leptonema_B	Alkaliphilus_B	1.00E+00	2.22E-16	2.21E-14	1.90E-06
Lutimonas_B	Alkaliphilus_B	1.00E+00	2.22E-16	2.21E-14	1.90E-06
Massilia_B	Alkaliphilus_B	1.00E+00	2.22E-16	2.21E-14	1.90E-06
Parasegetibacter_B	Alkaliphilus_B	-9.42E-01	2.86E-06	2.46E-04	7.84E-03
Schwartzia_B	Alkaliphilus_B	-9.26E-01	9.83E-06	7.85E-04	7.84E-03
Tepidanaerobacter_B	Alkaliphilus_B	1.00E+00	2.22E-16	2.21E-14	1.90E-06
Thermoflavimicrobium_B	Alkaliphilus_B	1.00E+00	2.22E-16	2.21E-14	1.90E-06
Unc_Verticillium_F	Alkaliphilus_B	1.00E+00	2.22E-16	2.21E-14	1.90E-06
Chattonella_F	Allisonella_B	8.35E-01	5.52E-04	2.99E-02	2.17E-01
Succinivibrio_B	Allisonella_B	1.00E+00	2.22E-16	2.21E-14	1.90E-06

Taphrina_F	Allisonella_B	1.00E+00	2.22E-16	2.21E-14	1.90E-06
Coprococcus_B	Allobaculum_B	-8.37E-01	5.21E-04	2.84E-02	2.17E-01
Flexithrix_B	Allobaculum_B	-9.08E-01	2.96E-05	2.09E-03	3.13E-02
Gracilibacter_B	Allobaculum_B	8.66E-01	1.97E-04	1.18E-02	1.04E-01
Moryella_B	Allobaculum_B	-8.62E-01	2.28E-04	1.34E-02	2.17E-01
Protomyces_F	Allobaculum_B	-9.02E-01	4.09E-05	2.83E-03	4.17E-02
Robinsoniella_B	Allobaculum_B	-8.22E-01	8.06E-04	4.12E-02	2.68E-01
Papillibacter_B	Allofustis_B	-8.50E-01	3.41E-04	1.95E-02	2.17E-01
Anaerofilum_B	Alloscardovia_B	-9.21E-01	1.40E-05	1.07E-03	7.84E-03
Atopobacter_B	Alloscardovia_B	1.00E+00	2.22E-16	2.21E-14	1.90E-06
Bellilinea_B	Alloscardovia_B	-8.66E-01	1.97E-04	1.17E-02	1.04E-01
Blautia_B	Alloscardovia_B	-8.83E-01	9.96E-05	6.43E-03	6.06E-02
Catonella_B	Alloscardovia_B	-8.73E-01	1.53E-04	9.35E-03	1.04E-01
Clostridiisalibacter_B	Alloscardovia_B	1.00E+00	2.22E-16	2.21E-14	1.90E-06
Desulforegula_B	Alloscardovia_B	1.00E+00	2.22E-16	2.21E-14	1.90E-06
Enterorhabdus_B	Alloscardovia_B	-9.17E-01	1.75E-05	1.30E-03	7.84E-03
Haloglycomyces_B	Alloscardovia_B	1.00E+00	2.22E-16	2.21E-14	1.90E-06
Hespellia_B	Alloscardovia_B	-9.70E-01	1.04E-07	9.77E-06	3.19E-04
Microscilla_B	Alloscardovia_B	1.00E+00	2.22E-16	2.21E-14	1.90E-06
Petrimonas_B	Alloscardovia_B	1.00E+00	2.22E-16	2.21E-14	1.90E-06
Porphyromonas_B	Alloscardovia_B	1.00E+00	2.22E-16	2.21E-14	1.90E-06
Propionibacterium_B	Alloscardovia_B	1.00E+00	2.22E-16	2.21E-14	1.90E-06
Pseudomonas_B	Alloscardovia_B	1.00E+00	2.22E-16	2.21E-14	1.90E-06
Sanguibacter_B	Alloscardovia_B	1.00E+00	2.22E-16	2.21E-14	1.90E-06
Shuttleworthia_B	Alloscardovia_B	-8.88E-01	8.23E-05	5.41E-03	6.01E-02
Spirochaeta_B	Alloscardovia_B	1.00E+00	2.22E-16	2.21E-14	1.90E-06
unclassified_F	Alloscardovia_B	-8.44E-01	4.25E-04	2.37E-02	2.17E-01
Anaerostipes_B	Alternaria_F	8.13E-01	1.01E-03	4.96E-02	2.68E-01
Anaerosphaera_B	Ammonifex_B	1.00E+00	2.22E-16	2.21E-14	1.90E-06
Atopococcus_B	Ammonifex_B	1.00E+00	2.22E-16	2.21E-14	1.90E-06

Bacillus_B	Ammonifex_B	1.00E+00	2.22E-16	2.21E-14	1.90E-06
Caldilinea_B	Ammonifex_B	1.00E+00	2.22E-16	2.21E-14	1.90E-06
Denitrobacterium_B	Ammonifex_B	1.00E+00	2.22E-16	2.21E-14	1.90E-06
Desulfocurvus_B	Ammonifex_B	1.00E+00	2.22E-16	2.21E-14	1.90E-06
Desulfoluna_B	Ammonifex_B	1.00E+00	2.22E-16	2.21E-14	1.90E-06
Erysipelothrix_B	Ammonifex_B	-9.29E-01	8.34E-06	6.77E-04	7.84E-03
Filimonas_B	Ammonifex_B	1.00E+00	2.22E-16	2.21E-14	1.90E-06
Kineococcus_B	Ammonifex_B	1.00E+00	2.22E-16	2.21E-14	1.90E-06
Nesiotobacter_B	Ammonifex_B	1.00E+00	2.22E-16	2.21E-14	1.90E-06
Nubsella_B	Ammonifex_B	8.16E-01	9.31E-04	4.65E-02	2.68E-01
Oxalicibacterium_B	Ammonifex_B	1.00E+00	2.22E-16	2.21E-14	1.90E-06
Paracoccus_B	Ammonifex_B	1.00E+00	2.22E-16	2.21E-14	1.90E-06
Paralactobacillus_B	Ammonifex_B	1.00E+00	2.22E-16	2.21E-14	1.90E-06
Proteiniphilum_B	Ammonifex_B	1.00E+00	2.22E-16	2.21E-14	1.90E-06
Psychrobacter_B	Ammonifex_B	1.00E+00	2.22E-16	2.21E-14	1.90E-06
Sediminibacterium_B	Ammonifex_B	1.00E+00	2.22E-16	2.21E-14	1.90E-06
Selenomonas_B	Ammonifex_B	1.00E+00	2.22E-16	2.21E-14	1.90E-06
Stenoxybacter_B	Ammonifex_B	1.00E+00	2.22E-16	2.21E-14	1.90E-06
Talaromyces_F	Ammonifex_B	1.00E+00	2.22E-16	2.21E-14	1.90E-06
unclassified_Pezizomycotina_F	Ammonifex_B	-1.00E+00	2.22E-16	2.21E-14	1.90E-06
Anaeroplasma_B	Anaerobacter_B	-8.27E-01	6.91E-04	3.61E-02	2.17E-01
Catellibacterium_B	Anaerobacter_B	1.00E+00	2.22E-16	2.21E-14	1.90E-06
Cystofilobasidium_F	Anaerobacter_B	-9.50E-01	1.34E-06	1.19E-04	2.81E-03
Dechloromonas_B	Anaerobacter_B	1.00E+00	2.22E-16	2.21E-14	1.90E-06
Filobasidium_F	Anaerobacter_B	-9.68E-01	1.35E-07	1.26E-05	5.68E-04
Gemmatimonas_B	Anaerobacter_B	1.00E+00	2.22E-16	2.21E-14	1.90E-06
Geosporobacter_B	Anaerobacter_B	-9.12E-01	2.43E-05	1.74E-03	1.77E-02
Nocardioides_B	Anaerobacter_B	1.00E+00	2.22E-16	2.21E-14	1.90E-06
Olivibacter_B	Anaerobacter_B	-9.17E-01	1.75E-05	1.30E-03	7.84E-03
Pelomonas_B	Anaerobacter_B	1.00E+00	2.22E-16	2.21E-14	1.90E-06

Unc_Kirschsteiniothelia_F	Anaerobacter_B	-1.00E+00	2.22E-16	2.21E-14	1.90E-06
Verrucomicrobium_B	Anaerobacter_B	-8.93E-01	6.47E-05	4.33E-03	6.01E-02
Anaerotruncus_B	Anaerofilum_B	8.30E-01	6.36E-04	3.37E-02	2.17E-01
Atopobacter_B	Anaerofilum_B	-9.21E-01	1.40E-05	1.07E-03	7.84E-03
Blautia_B	Anaerofilum_B	9.08E-01	3.00E-05	2.12E-03	3.13E-02
Catonella_B	Anaerofilum_B	9.17E-01	1.82E-05	1.34E-03	7.84E-03
Clostridiisalibacter_B	Anaerofilum_B	-9.21E-01	1.40E-05	1.07E-03	7.84E-03
Desulforegula_B	Anaerofilum_B	-9.21E-01	1.40E-05	1.07E-03	7.84E-03
Enterorhabdus_B	Anaerofilum_B	9.06E-01	3.42E-05	2.39E-03	4.17E-02
Haloglycomyces_B	Anaerofilum_B	-9.21E-01	1.40E-05	1.07E-03	7.84E-03
Hespellia_B	Anaerofilum_B	9.06E-01	3.36E-05	2.35E-03	4.17E-02
Lactonifactor_B	Anaerofilum_B	8.61E-01	2.40E-04	1.41E-02	2.17E-01
Microscilla_B	Anaerofilum_B	-9.21E-01	1.40E-05	1.07E-03	7.84E-03
Petrimonas_B	Anaerofilum_B	-9.21E-01	1.40E-05	1.07E-03	7.84E-03
Porphyromonas_B	Anaerofilum_B	-9.21E-01	1.40E-05	1.07E-03	7.84E-03
Propionibacterium_B	Anaerofilum_B	-9.21E-01	1.40E-05	1.07E-03	7.84E-03
Pseudomonas_B	Anaerofilum_B	-9.21E-01	1.40E-05	1.07E-03	7.84E-03
Sanguibacter_B	Anaerofilum_B	-9.21E-01	1.40E-05	1.07E-03	7.84E-03
Sharpea_B	Anaerofilum_B	8.83E-01	1.02E-04	6.57E-03	6.06E-02
Spirochaeta_B	Anaerofilum_B	-9.21E-01	1.40E-05	1.07E-03	7.84E-03
Anaerovorax_B	Anaeroglobus_B	8.66E-01	2.00E-04	1.19E-02	1.04E-01
Kiloniella_B	Anaeroglobus_B	-9.72E-01	6.40E-08	6.11E-06	2.87E-04
Oribacterium_B	Anaeroglobus_B	8.52E-01	3.26E-04	1.87E-02	2.17E-01
Pelospora_B	Anaeroglobus_B	9.37E-01	4.57E-06	3.86E-04	7.84E-03
Sporobacterium_B	Anaeroglobus_B	9.70E-01	9.66E-08	9.12E-06	2.87E-04
Stenotrophomonas_B	Anaeroglobus_B	-9.72E-01	6.40E-08	6.11E-06	2.87E-04
Natantispora_F	Anaerophaga_B	8.17E-01	9.29E-04	4.64E-02	2.68E-01
Tepidimicrobium_B	Anaerophaga_B	8.17E-01	9.29E-04	4.64E-02	2.68E-01
Thermotalea_B	Anaerophaga_B	8.68E-01	1.82E-04	1.09E-02	1.04E-01
Aquiflexum_B	Anaeroplasma_B	8.30E-01	6.35E-04	3.36E-02	2.17E-01

Catellibacterium_B	Anaeroplasma_B	-8.27E-01	6.91E-04	3.61E-02	2.17E-01
Cystofilobasidium_F	Anaeroplasma_B	8.17E-01	9.14E-04	4.58E-02	2.68E-01
Dechloromonas_B	Anaeroplasma_B	-8.27E-01	6.91E-04	3.61E-02	2.17E-01
Gemmatimonas_B	Anaeroplasma_B	-8.27E-01	6.91E-04	3.61E-02	2.17E-01
Nocardioides_B	Anaeroplasma_B	-8.27E-01	6.91E-04	3.61E-02	2.17E-01
Olivibacter_B	Anaeroplasma_B	8.68E-01	1.86E-04	1.12E-02	1.04E-01
Pelomonas_B	Anaeroplasma_B	-8.27E-01	6.91E-04	3.61E-02	2.17E-01
Unc_Kirschsteiniothelia_F	Anaeroplasma_B	8.27E-01	6.91E-04	3.61E-02	2.17E-01
Atopococcus_B	Anaerosphaera_B	1.00E+00	2.22E-16	2.21E-14	1.90E-06
Bacillus_B	Anaerosphaera_B	1.00E+00	2.22E-16	2.21E-14	1.90E-06
Caldilinea_B	Anaerosphaera_B	1.00E+00	2.22E-16	2.21E-14	1.90E-06
Denitrobacterium_B	Anaerosphaera_B	1.00E+00	2.22E-16	2.21E-14	1.90E-06
Desulfocurvus_B	Anaerosphaera_B	1.00E+00	2.22E-16	2.21E-14	1.90E-06
Desulfoluna_B	Anaerosphaera_B	1.00E+00	2.22E-16	2.21E-14	1.90E-06
Erysipelothrix_B	Anaerosphaera_B	-9.29E-01	8.34E-06	6.77E-04	7.84E-03
Filimonas_B	Anaerosphaera_B	1.00E+00	2.22E-16	2.21E-14	1.90E-06
Kineococcus_B	Anaerosphaera_B	1.00E+00	2.22E-16	2.21E-14	1.90E-06
Nesiotobacter_B	Anaerosphaera_B	1.00E+00	2.22E-16	2.21E-14	1.90E-06
Nubsella_B	Anaerosphaera_B	8.16E-01	9.31E-04	4.65E-02	2.68E-01
Oxalicibacterium_B	Anaerosphaera_B	1.00E+00	2.22E-16	2.21E-14	1.90E-06
Paracoccus_B	Anaerosphaera_B	1.00E+00	2.22E-16	2.21E-14	1.90E-06
Paralactobacillus_B	Anaerosphaera_B	1.00E+00	2.22E-16	2.21E-14	1.90E-06
Proteiniphilum_B	Anaerosphaera_B	1.00E+00	2.22E-16	2.21E-14	1.90E-06
Psychrobacter_B	Anaerosphaera_B	1.00E+00	2.22E-16	2.21E-14	1.90E-06
Sediminibacterium_B	Anaerosphaera_B	1.00E+00	2.22E-16	2.21E-14	1.90E-06
Selenomonas_B	Anaerosphaera_B	1.00E+00	2.22E-16	2.21E-14	1.90E-06
Stenoxybacter_B	Anaerosphaera_B	1.00E+00	2.22E-16	2.21E-14	1.90E-06
Talaromyces_F	Anaerosphaera_B	1.00E+00	2.22E-16	2.21E-14	1.90E-06
unclassified_Pezizomycotina_F	Anaerosphaera_B	-1.00E+00	2.22E-16	2.21E-14	1.90E-06
Arthrobacter_B	Anaerosporobacter_B	-9.13E-01	2.26E-05	1.63E-03	1.77E-02

Clavibacter_B	Anaerosporobacter_B	-9.13E-01	2.26E-05	1.63E-03	1.77E-02
Caminicella_B	Anaerostipes_B	8.24E-01	7.63E-04	3.93E-02	2.68E-01
Coprococcus_B	Anaerostipes_B	8.50E-01	3.50E-04	1.99E-02	2.17E-01
Roseburia_B	Anaerostipes_B	9.16E-01	1.86E-05	1.36E-03	7.84E-03
Catonella_B	Anaerotruncus_B	8.79E-01	1.17E-04	7.42E-03	6.06E-02
Enterorhabdus_B	Anaerotruncus_B	9.01E-01	4.37E-05	3.01E-03	4.17E-02
Fastidiosipila_B	Anaerotruncus_B	9.65E-01	2.15E-07	2.00E-05	2.21E-03
Oscillibacter_B	Anaerotruncus_B	8.25E-01	7.42E-04	3.84E-02	2.68E-01
Solobacterium_B	Anaerotruncus_B	8.77E-01	1.32E-04	8.22E-03	6.06E-02
Xylanibacter_B	Anaerotruncus_B	8.49E-01	3.58E-04	2.03E-02	2.17E-01
Sandaracinobacter_B	Anaerovibrio_B	8.31E-01	6.29E-04	3.34E-02	2.17E-01
Kiloniella_B	Anaerovorax_B	-8.85E-01	9.21E-05	5.99E-03	6.01E-02
Parasporobacterium_B	Anaerovorax_B	8.34E-01	5.75E-04	3.09E-02	2.17E-01
Pelospora_B	Anaerovorax_B	8.85E-01	9.16E-05	5.96E-03	6.01E-02
Sporobacterium_B	Anaerovorax_B	9.12E-01	2.42E-05	1.73E-03	1.77E-02
Stenotrophomonas_B	Anaerovorax_B	-8.85E-01	9.21E-05	5.99E-03	6.01E-02
Limibacter_B	Anoxynatronum_B	8.26E-01	7.19E-04	3.74E-02	2.68E-01
Clavibacter_B	Arthrobacter_B	1.00E+00	2.22E-16	2.21E-14	1.90E-06
Centipeda_B	Asaccharobacter_B	8.36E-01	5.31E-04	2.89E-02	2.17E-01
Dorea_B	Asaccharobacter_B	9.90E-01	3.42E-10	3.36E-08	7.72E-06
Treponema_B	Ascosalsum_F	-9.43E-01	2.61E-06	2.25E-04	4.74E-03
Echinicola_B	Asteroleplasma_B	1.00E+00	2.22E-16	2.21E-14	1.90E-06
Fluviicola_B	Asteroleplasma_B	8.56E-01	2.86E-04	1.65E-02	2.17E-01
Jonquetella_B	Asteroleplasma_B	-8.40E-01	4.73E-04	2.61E-02	2.17E-01
Lutaonella_B	Asteroleplasma_B	8.91E-01	7.15E-05	4.76E-03	6.01E-02
Bellilinea_B	Atopobacter_B	-8.66E-01	1.97E-04	1.17E-02	1.04E-01
Blautia_B	Atopobacter_B	-8.83E-01	9.96E-05	6.43E-03	6.06E-02
Catonella_B	Atopobacter_B	-8.73E-01	1.53E-04	9.35E-03	1.04E-01
Clostridiisalibacter_B	Atopobacter_B	1.00E+00	2.22E-16	2.21E-14	1.90E-06
Desulforegula_B	Atopobacter_B	1.00E+00	2.22E-16	2.21E-14	1.90E-06

Enterorhabdus_B	Atopobacter_B	-9.17E-01	1.75E-05	1.30E-03	7.84E-03
Haloglycomyces_B	Atopobacter_B	1.00E+00	2.22E-16	2.21E-14	1.90E-06
Hespellia_B	Atopobacter_B	-9.70E-01	1.04E-07	9.77E-06	3.19E-04
Microscilla_B	Atopobacter_B	1.00E+00	2.22E-16	2.21E-14	1.90E-06
Petrimonas_B	Atopobacter_B	1.00E+00	2.22E-16	2.21E-14	1.90E-06
Porphyromonas_B	Atopobacter_B	1.00E+00	2.22E-16	2.21E-14	1.90E-06
Propionibacterium_B	Atopobacter_B	1.00E+00	2.22E-16	2.21E-14	1.90E-06
Pseudomonas_B	Atopobacter_B	1.00E+00	2.22E-16	2.21E-14	1.90E-06
Sanguibacter_B	Atopobacter_B	1.00E+00	2.22E-16	2.21E-14	1.90E-06
Shuttleworthia_B	Atopobacter_B	-8.88E-01	8.23E-05	5.41E-03	6.01E-02
Spirochaeta_B	Atopobacter_B	1.00E+00	2.22E-16	2.21E-14	1.90E-06
unclassified_F	Atopobacter_B	-8.44E-01	4.25E-04	2.37E-02	2.17E-01
Brevibacterium_B	Atopobium_B	-8.85E-01	9.16E-05	5.96E-03	6.01E-02
Desulfonatronospira_B	Atopobium_B	8.38E-01	5.10E-04	2.79E-02	2.17E-01
Haliscomenobacter_B	Atopobium_B	-8.28E-01	6.78E-04	3.55E-02	2.17E-01
Sporobacter_B	Atopobium_B	9.40E-01	3.49E-06	2.98E-04	7.84E-03
Bacillus_B	Atopococcus_B	1.00E+00	2.22E-16	2.21E-14	1.90E-06
Caldilinea_B	Atopococcus_B	1.00E+00	2.22E-16	2.21E-14	1.90E-06
Denitrobacterium_B	Atopococcus_B	1.00E+00	2.22E-16	2.21E-14	1.90E-06
Desulfocurvus_B	Atopococcus_B	1.00E+00	2.22E-16	2.21E-14	1.90E-06
Desulfoluna_B	Atopococcus_B	1.00E+00	2.22E-16	2.21E-14	1.90E-06
Erysipelothrix_B	Atopococcus_B	-9.29E-01	8.34E-06	6.77E-04	7.84E-03
Filimonas_B	Atopococcus_B	1.00E+00	2.22E-16	2.21E-14	1.90E-06
Kineococcus_B	Atopococcus_B	1.00E+00	2.22E-16	2.21E-14	1.90E-06
Nesiotobacter_B	Atopococcus_B	1.00E+00	2.22E-16	2.21E-14	1.90E-06
Nubsella_B	Atopococcus_B	8.16E-01	9.31E-04	4.65E-02	2.68E-01
Oxalicibacterium_B	Atopococcus_B	1.00E+00	2.22E-16	2.21E-14	1.90E-06
Paracoccus_B	Atopococcus_B	1.00E+00	2.22E-16	2.21E-14	1.90E-06
Paralactobacillus_B	Atopococcus_B	1.00E+00	2.22E-16	2.21E-14	1.90E-06
Proteiniphilum_B	Atopococcus_B	1.00E+00	2.22E-16	2.21E-14	1.90E-06

Psychrobacter_B	Atopococcus_B	1.00E+00	2.22E-16	2.21E-14	1.90E-06
Sediminibacterium_B	Atopococcus_B	1.00E+00	2.22E-16	2.21E-14	1.90E-06
Selenomonas_B	Atopococcus_B	1.00E+00	2.22E-16	2.21E-14	1.90E-06
Stenoxybacter_B	Atopococcus_B	1.00E+00	2.22E-16	2.21E-14	1.90E-06
Talaromyces_F	Atopococcus_B	1.00E+00	2.22E-16	2.21E-14	1.90E-06
unclassified_Pezizomycotina_F	Atopococcus_B	-1.00E+00	2.22E-16	2.21E-14	1.90E-06
Ornithobacterium_B	Atopostipes_B	-8.18E-01	8.99E-04	4.52E-02	2.68E-01
Caldilinea_B	Bacillus_B	1.00E+00	2.22E-16	2.21E-14	1.90E-06
Denitrobacterium_B	Bacillus_B	1.00E+00	2.22E-16	2.21E-14	1.90E-06
Desulfocurvus_B	Bacillus_B	1.00E+00	2.22E-16	2.21E-14	1.90E-06
Desulfoluna_B	Bacillus_B	1.00E+00	2.22E-16	2.21E-14	1.90E-06
Erysipelothrix_B	Bacillus_B	-9.29E-01	8.34E-06	6.77E-04	7.84E-03
Filimonas_B	Bacillus_B	1.00E+00	2.22E-16	2.21E-14	1.90E-06
Kineococcus_B	Bacillus_B	1.00E+00	2.22E-16	2.21E-14	1.90E-06
Nesiotobacter_B	Bacillus_B	1.00E+00	2.22E-16	2.21E-14	1.90E-06
Nubsella_B	Bacillus_B	8.16E-01	9.31E-04	4.65E-02	2.68E-01
Oxalicibacterium_B	Bacillus_B	1.00E+00	2.22E-16	2.21E-14	1.90E-06
Paracoccus_B	Bacillus_B	1.00E+00	2.22E-16	2.21E-14	1.90E-06
Paralactobacillus_B	Bacillus_B	1.00E+00	2.22E-16	2.21E-14	1.90E-06
Proteiniphilum_B	Bacillus_B	1.00E+00	2.22E-16	2.21E-14	1.90E-06
Psychrobacter_B	Bacillus_B	1.00E+00	2.22E-16	2.21E-14	1.90E-06
Sediminibacterium_B	Bacillus_B	1.00E+00	2.22E-16	2.21E-14	1.90E-06
Selenomonas_B	Bacillus_B	1.00E+00	2.22E-16	2.21E-14	1.90E-06
Stenoxybacter_B	Bacillus_B	1.00E+00	2.22E-16	2.21E-14	1.90E-06
Talaromyces_F	Bacillus_B	1.00E+00	2.22E-16	2.21E-14	1.90E-06
unclassified_Pezizomycotina_F	Bacillus_B	-1.00E+00	2.22E-16	2.21E-14	1.90E-06
Phocaeicola_B	Bacteroides_B	-8.25E-01	7.39E-04	3.82E-02	2.68E-01
Sporacetigenium_B	Bacteroides_B	-8.87E-01	8.37E-05	5.49E-03	6.01E-02
Bimuria_F	Bavariicoccus_B	-8.99E-01	4.85E-05	3.31E-03	4.24E-02
Brachymonas_B	Bavariicoccus_B	-8.99E-01	4.85E-05	3.31E-03	4.24E-02

Desemzia_B	Bavariicoccus_B	-8.99E-01	4.85E-05	3.31E-03	4.24E-02
Desulfatiferula_B	Bavariicoccus_B	-8.99E-01	4.85E-05	3.31E-03	4.24E-02
Holtermannia_F	Bavariicoccus_B	-8.99E-01	4.85E-05	3.31E-03	4.24E-02
Leuconostoc_B	Bavariicoccus_B	-8.99E-01	4.85E-05	3.31E-03	4.24E-02
Phascolarctobacterium_B	Bavariicoccus_B	-8.99E-01	4.85E-05	3.31E-03	4.24E-02
Pirellula_B	Bavariicoccus_B	-8.99E-01	4.85E-05	3.31E-03	4.24E-02
Rhodopirellula_B	Bavariicoccus_B	8.50E-01	3.45E-04	1.97E-02	2.17E-01
Rudanella_B	Bavariicoccus_B	-8.99E-01	4.85E-05	3.31E-03	4.24E-02
Thermonema_B	Bavariicoccus_B	-8.99E-01	4.85E-05	3.31E-03	4.24E-02
Tropheryma_B	Bavariicoccus_B	-8.99E-01	4.85E-05	3.31E-03	4.24E-02
Unc_Cryptococcus_F	Bavariicoccus_B	-8.99E-01	4.85E-05	3.31E-03	4.24E-02
Verminephrobacter_B	Bavariicoccus_B	-8.99E-01	4.85E-05	3.31E-03	4.24E-02
Kosmotoga_B	Bdellovibrio_B	1.00E+00	2.22E-16	2.21E-14	1.90E-06
Shuttleworthia_B	Bdellovibrio_B	-8.48E-01	3.74E-04	2.12E-02	2.17E-01
Clostridiisalibacter_B	Bellilinea_B	-8.66E-01	1.97E-04	1.17E-02	1.04E-01
Desulforegula_B	Bellilinea_B	-8.66E-01	1.97E-04	1.17E-02	1.04E-01
Eubacterium_B	Bellilinea_B	8.38E-01	5.03E-04	2.75E-02	2.17E-01
Filobacillus_B	Bellilinea_B	-8.18E-01	9.00E-04	4.52E-02	2.68E-01
Haloglycomyces_B	Bellilinea_B	-8.66E-01	1.97E-04	1.17E-02	1.04E-01
Hespellia_B	Bellilinea_B	8.64E-01	2.15E-04	1.27E-02	1.04E-01
Microscilla_B	Bellilinea_B	-8.66E-01	1.97E-04	1.17E-02	1.04E-01
Petrimonas_B	Bellilinea_B	-8.66E-01	1.97E-04	1.17E-02	1.04E-01
Porphyromonas_B	Bellilinea_B	-8.66E-01	1.97E-04	1.17E-02	1.04E-01
Propionibacterium_B	Bellilinea_B	-8.66E-01	1.97E-04	1.17E-02	1.04E-01
Pseudomonas_B	Bellilinea_B	-8.66E-01	1.97E-04	1.17E-02	1.04E-01
Sanguibacter_B	Bellilinea_B	-8.66E-01	1.97E-04	1.17E-02	1.04E-01
Shuttleworthia_B	Bellilinea_B	9.19E-01	1.61E-05	1.21E-03	7.84E-03
Spirochaeta_B	Bellilinea_B	-8.66E-01	1.97E-04	1.17E-02	1.04E-01
unclassified_F	Bellilinea_B	8.84E-01	9.70E-05	6.27E-03	6.01E-02
Gordonibacter_B	Bibersteinia_B	-8.23E-01	7.89E-04	4.04E-02	2.68E-01

Pedobacter_B	Bibersteinia_B	9.91E-01	1.88E-10	1.85E-08	5.47E-06
Brachymonas_B	Bimuria_F	1.00E+00	2.22E-16	2.21E-14	1.90E-06
Bulleidia_B	Bimuria_F	8.79E-01	1.18E-04	7.48E-03	6.06E-02
Desemzia_B	Bimuria_F	1.00E+00	2.22E-16	2.21E-14	1.90E-06
Desulfatiferula_B	Bimuria_F	1.00E+00	2.22E-16	2.21E-14	1.90E-06
Holtermannia_F	Bimuria_F	1.00E+00	2.22E-16	2.21E-14	1.90E-06
Leuconostoc_B	Bimuria_F	1.00E+00	2.22E-16	2.21E-14	1.90E-06
Levilinea_B	Bimuria_F	-9.17E-01	1.75E-05	1.30E-03	7.84E-03
Parasporobacterium_B	Bimuria_F	8.63E-01	2.19E-04	1.29E-02	2.17E-01
Phascolarctobacterium_B	Bimuria_F	1.00E+00	2.22E-16	2.21E-14	1.90E-06
Pirellula_B	Bimuria_F	1.00E+00	2.22E-16	2.21E-14	1.90E-06
Rhodopirellula_B	Bimuria_F	-8.76E-01	1.33E-04	8.28E-03	7.38E-02
Rudanella_B	Bimuria_F	1.00E+00	2.22E-16	2.21E-14	1.90E-06
Thermonema_B	Bimuria_F	1.00E+00	2.22E-16	2.21E-14	1.90E-06
Tropheryma_B	Bimuria_F	1.00E+00	2.22E-16	2.21E-14	1.90E-06
Unc_Cryptococcus_F	Bimuria_F	1.00E+00	2.22E-16	2.21E-14	1.90E-06
Verminephrobacter_B	Bimuria_F	1.00E+00	2.22E-16	2.21E-14	1.90E-06
Catenibacterium_B	Blautia_B	-8.40E-01	4.79E-04	2.64E-02	2.17E-01
Catonella_B	Blautia_B	9.00E-01	4.70E-05	3.22E-03	4.17E-02
Clostridiisalibacter_B	Blautia_B	-8.83E-01	9.96E-05	6.43E-03	6.06E-02
Desulforegula_B	Blautia_B	-8.83E-01	9.96E-05	6.43E-03	6.06E-02
Enterorhabdus_B	Blautia_B	9.52E-01	1.12E-06	1.00E-04	2.81E-03
Haloglycomyces_B	Blautia_B	-8.83E-01	9.96E-05	6.43E-03	6.06E-02
Hespellia_B	Blautia_B	9.04E-01	3.83E-05	2.66E-03	4.17E-02
Microscilla_B	Blautia_B	-8.83E-01	9.96E-05	6.43E-03	6.06E-02
Petrimonas_B	Blautia_B	-8.83E-01	9.96E-05	6.43E-03	6.06E-02
Porphyromonas_B	Blautia_B	-8.83E-01	9.96E-05	6.43E-03	6.06E-02
Propionibacterium_B	Blautia_B	-8.83E-01	9.96E-05	6.43E-03	6.06E-02
Pseudomonas_B	Blautia_B	-8.83E-01	9.96E-05	6.43E-03	6.06E-02
Robinsoniella_B	Blautia_B	8.71E-01	1.64E-04	9.98E-03	1.04E-01

Sanguibacter_B	Blautia_B	-8.83E-01	9.96E-05	6.43E-03	6.06E-02
Shuttleworthia_B	Blautia_B	8.97E-01	5.33E-05	3.62E-03	6.01E-02
Spirochaeta_B	Blautia_B	-8.83E-01	9.96E-05	6.43E-03	6.06E-02
Syntrophococcus_B	Blautia_B	9.00E-01	4.56E-05	3.13E-03	4.17E-02
Turicibacter_B	Blautia_B	-8.82E-01	1.04E-04	6.65E-03	6.06E-02
Melissococcus_B	Brachybacterium_B	-9.72E-01	6.40E-08	6.11E-06	2.87E-04
Bulleidia_B	Brachymonas_B	8.79E-01	1.18E-04	7.48E-03	6.06E-02
Desemzia_B	Brachymonas_B	1.00E+00	2.22E-16	2.21E-14	1.90E-06
Desulfatiferula_B	Brachymonas_B	1.00E+00	2.22E-16	2.21E-14	1.90E-06
Holtermannia_F	Brachymonas_B	1.00E+00	2.22E-16	2.21E-14	1.90E-06
Leuconostoc_B	Brachymonas_B	1.00E+00	2.22E-16	2.21E-14	1.90E-06
Levilinea_B	Brachymonas_B	-9.17E-01	1.75E-05	1.30E-03	7.84E-03
Parasporobacterium_B	Brachymonas_B	8.63E-01	2.19E-04	1.29E-02	2.17E-01
Phascolarctobacterium_B	Brachymonas_B	1.00E+00	2.22E-16	2.21E-14	1.90E-06
Pirellula_B	Brachymonas_B	1.00E+00	2.22E-16	2.21E-14	1.90E-06
Rhodopirellula_B	Brachymonas_B	-8.76E-01	1.33E-04	8.28E-03	7.38E-02
Rudanella_B	Brachymonas_B	1.00E+00	2.22E-16	2.21E-14	1.90E-06
Thermonema_B	Brachymonas_B	1.00E+00	2.22E-16	2.21E-14	1.90E-06
Tropheryma_B	Brachymonas_B	1.00E+00	2.22E-16	2.21E-14	1.90E-06
Unc_Cryptococcus_F	Brachymonas_B	1.00E+00	2.22E-16	2.21E-14	1.90E-06
Verminephrobacter_B	Brachymonas_B	1.00E+00	2.22E-16	2.21E-14	1.90E-06
Desulfonatronospira_B	Brevibacterium_B	-9.89E-01	4.51E-10	4.42E-08	2.81E-05
Cloacibacillus_B	Bulgaria_F	1.00E+00	2.22E-16	2.21E-14	1.90E-06
Conexibacter_B	Bulgaria_F	1.00E+00	2.22E-16	2.21E-14	1.90E-06
Dolosigranulum_B	Bulgaria_F	1.00E+00	2.22E-16	2.21E-14	1.90E-06
Effluviibacter_B	Bulgaria_F	-8.27E-01	6.99E-04	3.65E-02	2.68E-01
Kordia_B	Bulgaria_F	1.00E+00	2.22E-16	2.21E-14	1.90E-06
Labedella_B	Bulgaria_F	1.00E+00	2.22E-16	2.21E-14	1.90E-06
Nicoletella_B	Bulgaria_F	1.00E+00	2.22E-16	2.21E-14	1.90E-06
Okibacterium_B	Bulgaria_F	1.00E+00	2.22E-16	2.21E-14	1.90E-06

Olsenella_B	Bulgaria_F	-9.47E-01	1.78E-06	1.57E-04	2.81E-03
Parascardovia_B	Bulgaria_F	1.00E+00	2.22E-16	2.21E-14	1.90E-06
Rhodovarius_B	Bulgaria_F	1.00E+00	2.22E-16	2.21E-14	1.90E-06
Slackia_B	Bulgaria_F	-9.07E-01	3.20E-05	2.25E-03	4.17E-02
Thermovenabulum_B	Bulgaria_F	1.00E+00	2.22E-16	2.21E-14	1.90E-06
Desemzia_B	Bulleidia_B	8.79E-01	1.18E-04	7.48E-03	6.06E-02
Desulfatiferula_B	Bulleidia_B	8.79E-01	1.18E-04	7.48E-03	6.06E-02
Holtermannia_F	Bulleidia_B	8.79E-01	1.18E-04	7.48E-03	6.06E-02
Leuconostoc_B	Bulleidia_B	8.79E-01	1.18E-04	7.48E-03	6.06E-02
Levilinea_B	Bulleidia_B	-8.65E-01	2.08E-04	1.23E-02	1.04E-01
Natronobacillus_B	Bulleidia_B	8.32E-01	6.03E-04	3.22E-02	2.17E-01
Phascolarctobacterium_B	Bulleidia_B	8.79E-01	1.18E-04	7.48E-03	6.06E-02
Pirellula_B	Bulleidia_B	8.79E-01	1.18E-04	7.48E-03	6.06E-02
Rudanella_B	Bulleidia_B	8.79E-01	1.18E-04	7.48E-03	6.06E-02
Thermonema_B	Bulleidia_B	8.79E-01	1.18E-04	7.48E-03	6.06E-02
Tropheryma_B	Bulleidia_B	8.79E-01	1.18E-04	7.48E-03	6.06E-02
Unc_Cryptococcus_F	Bulleidia_B	8.79E-01	1.18E-04	7.48E-03	6.06E-02
Verminephrobacter_B	Bulleidia_B	8.79E-01	1.18E-04	7.48E-03	6.06E-02
Fabibacter_B	Butyricicoccus_B	-8.43E-01	4.32E-04	2.41E-02	2.17E-01
Proteiniborus_B	Butyricicoccus_B	-9.06E-01	3.38E-05	2.36E-03	4.17E-02
Thermicanus_B	Butyricimonas_B	-8.28E-01	6.70E-04	3.52E-02	2.17E-01
Vulcanibacillus_B	Butyrivibrio_B	-8.55E-01	2.91E-04	1.68E-02	2.17E-01
Denitrobacterium_B	Caldilinea_B	1.00E+00	2.22E-16	2.21E-14	1.90E-06
Desulfocurvus_B	Caldilinea_B	1.00E+00	2.22E-16	2.21E-14	1.90E-06
Desulfoluna_B	Caldilinea_B	1.00E+00	2.22E-16	2.21E-14	1.90E-06
Erysipelothrix_B	Caldilinea_B	-9.29E-01	8.34E-06	6.77E-04	7.84E-03
Filimonas_B	Caldilinea_B	1.00E+00	2.22E-16	2.21E-14	1.90E-06
Kineococcus_B	Caldilinea_B	1.00E+00	2.22E-16	2.21E-14	1.90E-06
Nesiotobacter_B	Caldilinea_B	1.00E+00	2.22E-16	2.21E-14	1.90E-06
Nubsella_B	Caldilinea_B	8.16E-01	9.31E-04	4.65E-02	2.68E-01

Oxalicibacterium_B	Caldilinea_B	1.00E+00	2.22E-16	2.21E-14	1.90E-06
Paracoccus_B	Caldilinea_B	1.00E+00	2.22E-16	2.21E-14	1.90E-06
Paralactobacillus_B	Caldilinea_B	1.00E+00	2.22E-16	2.21E-14	1.90E-06
Proteiniphilum_B	Caldilinea_B	1.00E+00	2.22E-16	2.21E-14	1.90E-06
Psychrobacter_B	Caldilinea_B	1.00E+00	2.22E-16	2.21E-14	1.90E-06
Sediminibacterium_B	Caldilinea_B	1.00E+00	2.22E-16	2.21E-14	1.90E-06
Selenomonas_B	Caldilinea_B	1.00E+00	2.22E-16	2.21E-14	1.90E-06
Stenoxybacter_B	Caldilinea_B	1.00E+00	2.22E-16	2.21E-14	1.90E-06
Talaromyces_F	Caldilinea_B	1.00E+00	2.22E-16	2.21E-14	1.90E-06
unclassified_Pezizomycotina_F	Caldilinea_B	-1.00E+00	2.22E-16	2.21E-14	1.90E-06
Cryptanaerobacter_B	Caldivirga_A	1.00E+00	2.22E-16	2.21E-14	1.90E-06
Eremococcus_B	Caldivirga_A	1.00E+00	2.22E-16	2.21E-14	1.90E-06
Fibrobacter_B	Caldivirga_A	-8.30E-01	6.44E-04	3.40E-02	2.17E-01
Flammeovirga_B	Caldivirga_A	1.00E+00	2.22E-16	2.21E-14	1.90E-06
Gracilimonas_B	Caldivirga_A	1.00E+00	2.22E-16	2.21E-14	1.90E-06
Halogeometricum_A	Caldivirga_A	1.00E+00	2.22E-16	2.21E-14	1.90E-06
Hydrogenobaculum_A	Caldivirga_A	1.00E+00	2.22E-16	2.21E-14	1.90E-06
Myceligenerans_B	Caldivirga_A	1.00E+00	2.22E-16	2.21E-14	1.90E-06
Otidea_F	Caldivirga_A	1.00E+00	2.22E-16	2.21E-14	1.90E-06
Quatrionicoccus_B	Caldivirga_A	1.00E+00	2.22E-16	2.21E-14	1.90E-06
Roseospirillum_B	Caldivirga_A	1.00E+00	2.22E-16	2.21E-14	1.90E-06
Thermodesulfobium_B	Caldivirga_A	1.00E+00	2.22E-16	2.21E-14	1.90E-06
Yaniella_B	Caldivirga_A	1.00E+00	2.22E-16	2.21E-14	1.90E-06
Roseburia_B	Caminicella_B	8.76E-01	1.38E-04	8.53E-03	7.38E-02
Cystofilobasidium_F	Catellibacterium_B	-9.50E-01	1.34E-06	1.19E-04	2.81E-03
Dechloromonas_B	Catellibacterium_B	1.00E+00	2.22E-16	2.21E-14	1.90E-06
Filobasidium_F	Catellibacterium_B	-9.68E-01	1.35E-07	1.26E-05	5.68E-04
Gemmatimonas_B	Catellibacterium_B	1.00E+00	2.22E-16	2.21E-14	1.90E-06
Geosporobacter_B	Catellibacterium_B	-9.12E-01	2.43E-05	1.74E-03	1.77E-02
Nocardioides_B	Catellibacterium_B	1.00E+00	2.22E-16	2.21E-14	1.90E-06

Olivibacter_B	Catellibacterium_B	-9.17E-01	1.75E-05	1.30E-03	7.84E-03
Pelomonas_B	Catellibacterium_B	1.00E+00	2.22E-16	2.21E-14	1.90E-06
Unc_Kirschsteiniothelia_F	Catellibacterium_B	-1.00E+00	2.22E-16	2.21E-14	1.90E-06
Verrucomicrobium_B	Catellibacterium_B	-8.93E-01	6.47E-05	4.33E-03	6.01E-02
Desulfovibrio_B	Catenibacterium_B	9.25E-01	1.07E-05	8.45E-04	7.84E-03
Hippea_B	Catenibacterium_B	-8.55E-01	2.94E-04	1.70E-02	2.17E-01
Prolixibacter_B	Catenibacterium_B	-9.03E-01	3.87E-05	2.68E-03	4.17E-02
Rhodothermus_B	Catenibacterium_B	9.25E-01	1.07E-05	8.45E-04	7.84E-03
Shuttleworthia_B	Catenibacterium_B	-8.24E-01	7.62E-04	3.93E-02	2.68E-01
Clostridiisalibacter_B	Catonella_B	-8.73E-01	1.53E-04	9.35E-03	1.04E-01
Desulforegula_B	Catonella_B	-8.73E-01	1.53E-04	9.35E-03	1.04E-01
Enterorhabdus_B	Catonella_B	9.32E-01	6.71E-06	5.53E-04	7.84E-03
Fastidiosipila_B	Catonella_B	8.76E-01	1.37E-04	8.50E-03	7.38E-02
Haloglycomyces_B	Catonella_B	-8.73E-01	1.53E-04	9.35E-03	1.04E-01
Hespellia_B	Catonella_B	9.02E-01	4.15E-05	2.86E-03	4.17E-02
Lactonifactor_B	Catonella_B	9.50E-01	1.30E-06	1.16E-04	2.81E-03
Microscilla_B	Catonella_B	-8.73E-01	1.53E-04	9.35E-03	1.04E-01
Oscillibacter_B	Catonella_B	8.31E-01	6.23E-04	3.31E-02	2.17E-01
Petrimonas_B	Catonella_B	-8.73E-01	1.53E-04	9.35E-03	1.04E-01
Porphyromonas_B	Catonella_B	-8.73E-01	1.53E-04	9.35E-03	1.04E-01
Propionibacterium_B	Catonella_B	-8.73E-01	1.53E-04	9.35E-03	1.04E-01
Pseudomonas_B	Catonella_B	-8.73E-01	1.53E-04	9.35E-03	1.04E-01
Sanguibacter_B	Catonella_B	-8.73E-01	1.53E-04	9.35E-03	1.04E-01
Sharpea_B	Catonella_B	8.55E-01	2.93E-04	1.70E-02	2.17E-01
Spirochaeta_B	Catonella_B	-8.73E-01	1.53E-04	9.35E-03	1.04E-01
Syntrophococcus_B	Catonella_B	8.78E-01	1.24E-04	7.78E-03	6.06E-02
Turicibacter_B	Catonella_B	-8.28E-01	6.79E-04	3.56E-02	2.17E-01
Duganella_B	Centipeda_B	8.28E-01	6.84E-04	3.58E-02	2.17E-01
Jeotgalicoccus_B	Centipeda_B	8.36E-01	5.31E-04	2.89E-02	2.17E-01
Coprococcus_B	Cerasibacillus_B	-8.41E-01	4.62E-04	2.56E-02	2.17E-01

Pseudosphingobacterium_B	Cerasibacillus_B	-8.50E-01	3.46E-04	1.97E-02	2.17E-01
Subdoligranulum_B	Chattonella_F	-8.40E-01	4.73E-04	2.61E-02	2.17E-01
Succinivibrio_B	Chattonella_F	8.35E-01	5.52E-04	2.99E-02	2.17E-01
Taphrina_F	Chattonella_F	8.35E-01	5.52E-04	2.99E-02	2.17E-01
Conexibacter_B	Cloacibacillus_B	1.00E+00	2.22E-16	2.21E-14	1.90E-06
Dolosigranulum_B	Cloacibacillus_B	1.00E+00	2.22E-16	2.21E-14	1.90E-06
Effluviibacter_B	Cloacibacillus_B	-8.27E-01	6.99E-04	3.65E-02	2.68E-01
Kordia_B	Cloacibacillus_B	1.00E+00	2.22E-16	2.21E-14	1.90E-06
Labedella_B	Cloacibacillus_B	1.00E+00	2.22E-16	2.21E-14	1.90E-06
Nicoletella_B	Cloacibacillus_B	1.00E+00	2.22E-16	2.21E-14	1.90E-06
Okibacterium_B	Cloacibacillus_B	1.00E+00	2.22E-16	2.21E-14	1.90E-06
Olsenella_B	Cloacibacillus_B	-9.47E-01	1.78E-06	1.57E-04	2.81E-03
Parascardovia_B	Cloacibacillus_B	1.00E+00	2.22E-16	2.21E-14	1.90E-06
Rhodovarius_B	Cloacibacillus_B	1.00E+00	2.22E-16	2.21E-14	1.90E-06
Slackia_B	Cloacibacillus_B	-9.07E-01	3.20E-05	2.25E-03	4.17E-02
Thermovenabulum_B	Cloacibacillus_B	1.00E+00	2.22E-16	2.21E-14	1.90E-06
Desulforegula_B	Clostridiisalibacter_B	1.00E+00	2.22E-16	2.21E-14	1.90E-06
Enterorhabdus_B	Clostridiisalibacter_B	-9.17E-01	1.75E-05	1.30E-03	7.84E-03
Haloglycomyces_B	Clostridiisalibacter_B	1.00E+00	2.22E-16	2.21E-14	1.90E-06
Hespellia_B	Clostridiisalibacter_B	-9.70E-01	1.04E-07	9.77E-06	3.19E-04
Microscilla_B	Clostridiisalibacter_B	1.00E+00	2.22E-16	2.21E-14	1.90E-06
Petrimonas_B	Clostridiisalibacter_B	1.00E+00	2.22E-16	2.21E-14	1.90E-06
Porphyromonas_B	Clostridiisalibacter_B	1.00E+00	2.22E-16	2.21E-14	1.90E-06
Propionibacterium_B	Clostridiisalibacter_B	1.00E+00	2.22E-16	2.21E-14	1.90E-06
Pseudomonas_B	Clostridiisalibacter_B	1.00E+00	2.22E-16	2.21E-14	1.90E-06
Sanguibacter_B	Clostridiisalibacter_B	1.00E+00	2.22E-16	2.21E-14	1.90E-06
Shuttleworthia_B	Clostridiisalibacter_B	-8.88E-01	8.23E-05	5.41E-03	6.01E-02
Spirochaeta_B	Clostridiisalibacter_B	1.00E+00	2.22E-16	2.21E-14	1.90E-06
unclassified_F	Clostridiisalibacter_B	-8.44E-01	4.25E-04	2.37E-02	2.17E-01
Fulvimonas_B	Coenonia_B	1.00E+00	2.22E-16	2.21E-14	1.90E-06

Fulvivirga_B	Coenonia_B	1.00E+00	2.22E-16	2.21E-14	1.90E-06
Jonesia_B	Coenonia_B	1.00E+00	2.22E-16	2.21E-14	1.90E-06
Lishizhenia_B	Coenonia_B	1.00E+00	2.22E-16	2.21E-14	1.90E-06
Ornithobacterium_B	Coenonia_B	-9.11E-01	2.50E-05	1.78E-03	3.13E-02
Parachlamydia_B	Coenonia_B	1.00E+00	2.22E-16	2.21E-14	1.90E-06
Sphaerobacter_B	Coenonia_B	1.00E+00	2.22E-16	2.21E-14	1.90E-06
Sporanaerobacter_B	Coenonia_B	1.00E+00	2.22E-16	2.21E-14	1.90E-06
Unc_Kockovaella_F	Coenonia_B	1.00E+00	2.22E-16	2.21E-14	1.90E-06
Venenivibrio_B	Coenonia_B	1.00E+00	2.22E-16	2.21E-14	1.90E-06
Zunongwangia_B	Coenonia_B	1.00E+00	2.22E-16	2.21E-14	1.90E-06
Desmospora_B	Collinsella_B	-8.22E-01	7.97E-04	4.08E-02	2.68E-01
Haliscomenobacter_B	Collinsella_B	-8.43E-01	4.28E-04	2.39E-02	2.17E-01
Unc_Sarcinomyces_F	Collinsella_B	-8.25E-01	7.29E-04	3.78E-02	2.68E-01
Dolosigranulum_B	Conexibacter_B	1.00E+00	2.22E-16	2.21E-14	1.90E-06
Effluviibacter_B	Conexibacter_B	-8.27E-01	6.99E-04	3.65E-02	2.68E-01
Kordia_B	Conexibacter_B	1.00E+00	2.22E-16	2.21E-14	1.90E-06
Labedella_B	Conexibacter_B	1.00E+00	2.22E-16	2.21E-14	1.90E-06
Nicoletella_B	Conexibacter_B	1.00E+00	2.22E-16	2.21E-14	1.90E-06
Okibacterium_B	Conexibacter_B	1.00E+00	2.22E-16	2.21E-14	1.90E-06
Olsenella_B	Conexibacter_B	-9.47E-01	1.78E-06	1.57E-04	2.81E-03
Parascardovia_B	Conexibacter_B	1.00E+00	2.22E-16	2.21E-14	1.90E-06
Rhodovarius_B	Conexibacter_B	1.00E+00	2.22E-16	2.21E-14	1.90E-06
Slackia_B	Conexibacter_B	-9.07E-01	3.20E-05	2.25E-03	4.17E-02
Thermovenabulum_B	Conexibacter_B	1.00E+00	2.22E-16	2.21E-14	1.90E-06
Dendrosporobacter_B	Coprobacillus_B	-8.38E-01	5.09E-04	2.78E-02	2.17E-01
Facklamia_B	Coprobacillus_B	-8.38E-01	5.09E-04	2.78E-02	2.17E-01
Galbibacter_B	Coprobacillus_B	-8.38E-01	5.09E-04	2.78E-02	2.17E-01
Lacticigenium_B	Coprobacillus_B	-8.38E-01	5.09E-04	2.78E-02	2.17E-01
Methylobacterium_B	Coprobacillus_B	-8.38E-01	5.09E-04	2.78E-02	2.17E-01
Ornithinimicrobium_B	Coprobacillus_B	-8.38E-01	5.09E-04	2.78E-02	2.17E-01

Oxalophagus_B	Coprobacillus_B	8.98E-01	4.96E-05	3.38E-03	6.01E-02
Rhodopila_B	Coprobacillus_B	-8.38E-01	5.09E-04	2.78E-02	2.17E-01
Trichococcus_B	Coprobacillus_B	-8.38E-01	5.09E-04	2.78E-02	2.17E-01
Pseudosphingobacterium_B	Coprococcus_B	8.64E-01	2.12E-04	1.26E-02	1.04E-01
Roseburia_B	Coprococcus_B	9.09E-01	2.82E-05	1.99E-03	3.13E-02
Staphylococcus_B	Coprococcus_B	-9.45E-01	2.20E-06	1.91E-04	4.74E-03
Syntrophococcus_B	Coprococcus_B	8.75E-01	1.39E-04	8.62E-03	7.38E-02
Desulfotomaculum_B	Coriobacterium_B	1.00E+00	2.22E-16	2.21E-14	1.90E-06
Ganoderma_F	Coriobacterium_B	1.00E+00	2.22E-16	2.21E-14	1.90E-06
Geosmithia_putterillii_sensu_Pitt_F	Coriobacterium_B	-9.16E-01	1.90E-05	1.39E-03	1.77E-02
Johnsonella_B	Coriobacterium_B	1.00E+00	2.22E-16	2.21E-14	1.90E-06
Leptonema_B	Coriobacterium_B	1.00E+00	2.22E-16	2.21E-14	1.90E-06
Lutimonas_B	Coriobacterium_B	1.00E+00	2.22E-16	2.21E-14	1.90E-06
Massilia_B	Coriobacterium_B	1.00E+00	2.22E-16	2.21E-14	1.90E-06
Parasegetibacter_B	Coriobacterium_B	-9.42E-01	2.86E-06	2.46E-04	7.84E-03
Schwartzia_B	Coriobacterium_B	-9.26E-01	9.83E-06	7.85E-04	7.84E-03
Tepidanaerobacter_B	Coriobacterium_B	1.00E+00	2.22E-16	2.21E-14	1.90E-06
Thermoflavimicrobium_B	Coriobacterium_B	1.00E+00	2.22E-16	2.21E-14	1.90E-06
Unc_Verticillium_F	Coriobacterium_B	1.00E+00	2.22E-16	2.21E-14	1.90E-06
Sporotomaculum_B	Corynebacterium_B	-8.51E-01	3.41E-04	1.95E-02	2.17E-01
Eremococcus_B	Cryptanaerobacter_B	1.00E+00	2.22E-16	2.21E-14	1.90E-06
Fibrobacter_B	Cryptanaerobacter_B	-8.30E-01	6.44E-04	3.40E-02	2.17E-01
Flammeovirga_B	Cryptanaerobacter_B	1.00E+00	2.22E-16	2.21E-14	1.90E-06
Gracilimonas_B	Cryptanaerobacter_B	1.00E+00	2.22E-16	2.21E-14	1.90E-06
Halogeometricum_A	Cryptanaerobacter_B	1.00E+00	2.22E-16	2.21E-14	1.90E-06
Hydrogenobaculum_A	Cryptanaerobacter_B	1.00E+00	2.22E-16	2.21E-14	1.90E-06
Myceligenerans_B	Cryptanaerobacter_B	1.00E+00	2.22E-16	2.21E-14	1.90E-06
Otidea_F	Cryptanaerobacter_B	1.00E+00	2.22E-16	2.21E-14	1.90E-06
Quatrionicoccus_B	Cryptanaerobacter_B	1.00E+00	2.22E-16	2.21E-14	1.90E-06
Roseospirillum_B	Cryptanaerobacter_B	1.00E+00	2.22E-16	2.21E-14	1.90E-06

Thermodesulfobium_B	Cryptanaerobacter_B	1.00E+00	2.22E-16	2.21E-14	1.90E-06
Yaniella_B	Cryptanaerobacter_B	1.00E+00	2.22E-16	2.21E-14	1.90E-06
Desulfonispora_B	Curtobacterium_B	9.57E-01	6.48E-07	5.91E-05	2.21E-03
Holophaga_B	Curtobacterium_B	1.00E+00	2.22E-16	2.21E-14	1.90E-06
Dechloromonas_B	Cystofilobasidium_F	-9.50E-01	1.34E-06	1.19E-04	2.81E-03
Filobasidium_F	Cystofilobasidium_F	9.03E-01	3.99E-05	2.76E-03	4.17E-02
Gemmatimonas_B	Cystofilobasidium_F	-9.50E-01	1.34E-06	1.19E-04	2.81E-03
Geosporobacter_B	Cystofilobasidium_F	8.94E-01	6.31E-05	4.23E-03	6.01E-02
Nocardioides_B	Cystofilobasidium_F	-9.50E-01	1.34E-06	1.19E-04	2.81E-03
Olivibacter_B	Cystofilobasidium_F	8.32E-01	6.10E-04	3.25E-02	2.17E-01
Pelomonas_B	Cystofilobasidium_F	-9.50E-01	1.34E-06	1.19E-04	2.81E-03
Unc_Kirschsteiniothelia_F	Cystofilobasidium_F	9.50E-01	1.34E-06	1.19E-04	2.81E-03
Verrucomicrobium_B	Cystofilobasidium_F	9.29E-01	8.06E-06	6.55E-04	7.84E-03
Filobasidium_F	Dechloromonas_B	-9.68E-01	1.35E-07	1.26E-05	5.68E-04
Gemmatimonas_B	Dechloromonas_B	1.00E+00	2.22E-16	2.21E-14	1.90E-06
Geosporobacter_B	Dechloromonas_B	-9.12E-01	2.43E-05	1.74E-03	1.77E-02
Nocardioides_B	Dechloromonas_B	1.00E+00	2.22E-16	2.21E-14	1.90E-06
Olivibacter_B	Dechloromonas_B	-9.17E-01	1.75E-05	1.30E-03	7.84E-03
Pelomonas_B	Dechloromonas_B	1.00E+00	2.22E-16	2.21E-14	1.90E-06
Unc_Kirschsteiniothelia_F	Dechloromonas_B	-1.00E+00	2.22E-16	2.21E-14	1.90E-06
Verrucomicrobium_B	Dechloromonas_B	-8.93E-01	6.47E-05	4.33E-03	6.01E-02
Facklamia_B	Dendrosporobacter_B	1.00E+00	2.22E-16	2.21E-14	1.90E-06
Galbibacter_B	Dendrosporobacter_B	1.00E+00	2.22E-16	2.21E-14	1.90E-06
Lacticigenium_B	Dendrosporobacter_B	1.00E+00	2.22E-16	2.21E-14	1.90E-06
Methylobacterium_B	Dendrosporobacter_B	1.00E+00	2.22E-16	2.21E-14	1.90E-06
Ornithinimicrobium_B	Dendrosporobacter_B	1.00E+00	2.22E-16	2.21E-14	1.90E-06
Oxalophagus_B	Dendrosporobacter_B	-9.54E-01	8.65E-07	7.82E-05	2.81E-03
Pseudozobellia_B	Dendrosporobacter_B	-9.16E-01	1.90E-05	1.39E-03	1.77E-02
Rhodopila_B	Dendrosporobacter_B	1.00E+00	2.22E-16	2.21E-14	1.90E-06
Streptophyta_B	Dendrosporobacter_B	-9.16E-01	1.90E-05	1.39E-03	1.77E-02

Trichococcus_B	Dendrosporobacter_B	1.00E+00	2.22E-16	2.21E-14	1.90E-06
Desulfocurvus_B	Denitrobacterium_B	1.00E+00	2.22E-16	2.21E-14	1.90E-06
Desulfoluna_B	Denitrobacterium_B	1.00E+00	2.22E-16	2.21E-14	1.90E-06
Erysipelothrix_B	Denitrobacterium_B	-9.29E-01	8.34E-06	6.77E-04	7.84E-03
Filimonas_B	Denitrobacterium_B	1.00E+00	2.22E-16	2.21E-14	1.90E-06
Kineococcus_B	Denitrobacterium_B	1.00E+00	2.22E-16	2.21E-14	1.90E-06
Nesiotobacter_B	Denitrobacterium_B	1.00E+00	2.22E-16	2.21E-14	1.90E-06
Nubsella_B	Denitrobacterium_B	8.16E-01	9.31E-04	4.65E-02	2.68E-01
Oxalicibacterium_B	Denitrobacterium_B	1.00E+00	2.22E-16	2.21E-14	1.90E-06
Paracoccus_B	Denitrobacterium_B	1.00E+00	2.22E-16	2.21E-14	1.90E-06
Paralactobacillus_B	Denitrobacterium_B	1.00E+00	2.22E-16	2.21E-14	1.90E-06
Proteiniphilum_B	Denitrobacterium_B	1.00E+00	2.22E-16	2.21E-14	1.90E-06
Psychrobacter_B	Denitrobacterium_B	1.00E+00	2.22E-16	2.21E-14	1.90E-06
Sediminibacterium_B	Denitrobacterium_B	1.00E+00	2.22E-16	2.21E-14	1.90E-06
Selenomonas_B	Denitrobacterium_B	1.00E+00	2.22E-16	2.21E-14	1.90E-06
Stenoxybacter_B	Denitrobacterium_B	1.00E+00	2.22E-16	2.21E-14	1.90E-06
Talaromyces_F	Denitrobacterium_B	1.00E+00	2.22E-16	2.21E-14	1.90E-06
unclassified_Pezizomycotina_F	Denitrobacterium_B	-1.00E+00	2.22E-16	2.21E-14	1.90E-06
Desulfatiferula_B	Desemzia_B	1.00E+00	2.22E-16	2.21E-14	1.90E-06
Holtermannia_F	Desemzia_B	1.00E+00	2.22E-16	2.21E-14	1.90E-06
Leuconostoc_B	Desemzia_B	1.00E+00	2.22E-16	2.21E-14	1.90E-06
Levilinea_B	Desemzia_B	-9.17E-01	1.75E-05	1.30E-03	7.84E-03
Parasporobacterium_B	Desemzia_B	8.63E-01	2.19E-04	1.29E-02	2.17E-01
Phascolarctobacterium_B	Desemzia_B	1.00E+00	2.22E-16	2.21E-14	1.90E-06
Pirellula_B	Desemzia_B	1.00E+00	2.22E-16	2.21E-14	1.90E-06
Rhodopirellula_B	Desemzia_B	-8.76E-01	1.33E-04	8.28E-03	7.38E-02
Rudanella_B	Desemzia_B	1.00E+00	2.22E-16	2.21E-14	1.90E-06
Thermonema_B	Desemzia_B	1.00E+00	2.22E-16	2.21E-14	1.90E-06
Tropheryma_B	Desemzia_B	1.00E+00	2.22E-16	2.21E-14	1.90E-06
Unc_Cryptococcus_F	Desemzia_B	1.00E+00	2.22E-16	2.21E-14	1.90E-06

Verminephrobacter_B	Desemzia_B	1.00E+00	2.22E-16	2.21E-14	1.90E-06
Haliscomenobacter_B	Desmospora_B	8.47E-01	3.83E-04	2.16E-02	2.17E-01
Sporobacter_B	Desmospora_B	-8.28E-01	6.86E-04	3.59E-02	2.17E-01
Holtermannia_F	Desulfatiferula_B	1.00E+00	2.22E-16	2.21E-14	1.90E-06
Leuconostoc_B	Desulfatiferula_B	1.00E+00	2.22E-16	2.21E-14	1.90E-06
Levilinea_B	Desulfatiferula_B	-9.17E-01	1.75E-05	1.30E-03	7.84E-03
Parasporobacterium_B	Desulfatiferula_B	8.63E-01	2.19E-04	1.29E-02	2.17E-01
Phascolarctobacterium_B	Desulfatiferula_B	1.00E+00	2.22E-16	2.21E-14	1.90E-06
Pirellula_B	Desulfatiferula_B	1.00E+00	2.22E-16	2.21E-14	1.90E-06
Rhodopirellula_B	Desulfatiferula_B	-8.76E-01	1.33E-04	8.28E-03	7.38E-02
Rudanella_B	Desulfatiferula_B	1.00E+00	2.22E-16	2.21E-14	1.90E-06
Thermonema_B	Desulfatiferula_B	1.00E+00	2.22E-16	2.21E-14	1.90E-06
Tropheryma_B	Desulfatiferula_B	1.00E+00	2.22E-16	2.21E-14	1.90E-06
Unc_Cryptococcus_F	Desulfatiferula_B	1.00E+00	2.22E-16	2.21E-14	1.90E-06
Verminephrobacter_B	Desulfatiferula_B	1.00E+00	2.22E-16	2.21E-14	1.90E-06
Desulfoluna_B	Desulfocurvus_B	1.00E+00	2.22E-16	2.21E-14	1.90E-06
Erysipelothrix_B	Desulfocurvus_B	-9.29E-01	8.34E-06	6.77E-04	7.84E-03
Filimonas_B	Desulfocurvus_B	1.00E+00	2.22E-16	2.21E-14	1.90E-06
Kineococcus_B	Desulfocurvus_B	1.00E+00	2.22E-16	2.21E-14	1.90E-06
Nesiotobacter_B	Desulfocurvus_B	1.00E+00	2.22E-16	2.21E-14	1.90E-06
Nubsella_B	Desulfocurvus_B	8.16E-01	9.31E-04	4.65E-02	2.68E-01
Oxalicibacterium_B	Desulfocurvus_B	1.00E+00	2.22E-16	2.21E-14	1.90E-06
Paracoccus_B	Desulfocurvus_B	1.00E+00	2.22E-16	2.21E-14	1.90E-06
Paralactobacillus_B	Desulfocurvus_B	1.00E+00	2.22E-16	2.21E-14	1.90E-06
Proteiniphilum_B	Desulfocurvus_B	1.00E+00	2.22E-16	2.21E-14	1.90E-06
Psychrobacter_B	Desulfocurvus_B	1.00E+00	2.22E-16	2.21E-14	1.90E-06
Sediminibacterium_B	Desulfocurvus_B	1.00E+00	2.22E-16	2.21E-14	1.90E-06
Selenomonas_B	Desulfocurvus_B	1.00E+00	2.22E-16	2.21E-14	1.90E-06
Stenoxybacter_B	Desulfocurvus_B	1.00E+00	2.22E-16	2.21E-14	1.90E-06
Talaromyces_F	Desulfocurvus_B	1.00E+00	2.22E-16	2.21E-14	1.90E-06

unclassified_Pezizomycotina_F	Desulfocurvus_B	-1.00E+00	2.22E-16	2.21E-14	1.90E-06
Erysipelothrix_B	Desulfoluna_B	-9.29E-01	8.34E-06	6.77E-04	7.84E-03
Filimonas_B	Desulfoluna_B	1.00E+00	2.22E-16	2.21E-14	1.90E-06
Kineococcus_B	Desulfoluna_B	1.00E+00	2.22E-16	2.21E-14	1.90E-06
Nesiotobacter_B	Desulfoluna_B	1.00E+00	2.22E-16	2.21E-14	1.90E-06
Nubsella_B	Desulfoluna_B	8.16E-01	9.31E-04	4.65E-02	2.68E-01
Oxalicibacterium_B	Desulfoluna_B	1.00E+00	2.22E-16	2.21E-14	1.90E-06
Paracoccus_B	Desulfoluna_B	1.00E+00	2.22E-16	2.21E-14	1.90E-06
Paralactobacillus_B	Desulfoluna_B	1.00E+00	2.22E-16	2.21E-14	1.90E-06
Proteiniphilum_B	Desulfoluna_B	1.00E+00	2.22E-16	2.21E-14	1.90E-06
Psychrobacter_B	Desulfoluna_B	1.00E+00	2.22E-16	2.21E-14	1.90E-06
Sediminibacterium_B	Desulfoluna_B	1.00E+00	2.22E-16	2.21E-14	1.90E-06
Selenomonas_B	Desulfoluna_B	1.00E+00	2.22E-16	2.21E-14	1.90E-06
Stenoxybacter_B	Desulfoluna_B	1.00E+00	2.22E-16	2.21E-14	1.90E-06
Talaromyces_F	Desulfoluna_B	1.00E+00	2.22E-16	2.21E-14	1.90E-06
unclassified_Pezizomycotina_F	Desulfoluna_B	-1.00E+00	2.22E-16	2.21E-14	1.90E-06
Fusibacter_B	Desulfonatronovibrio_B	9.91E-01	1.88E-10	1.85E-08	5.47E-06
Hallella_B	Desulfonatronovibrio_B	8.94E-01	6.07E-05	4.09E-03	6.01E-02
Howardella_B	Desulfonatronovibrio_B	8.26E-01	7.24E-04	3.76E-02	2.68E-01
Parvimonas_B	Desulfonatronovibrio_B	-9.38E-01	3.95E-06	3.36E-04	7.84E-03
Ruminococcus_B	Desulfonatronovibrio_B	8.39E-01	4.85E-04	2.67E-02	2.17E-01
Scytinostroma_F	Desulfonatronovibrio_B	9.91E-01	1.88E-10	1.85E-08	5.47E-06
Holophaga_B	Desulfonispora_B	9.57E-01	6.48E-07	5.91E-05	2.21E-03
Howardella_B	Desulfonispora_B	8.55E-01	2.88E-04	1.67E-02	2.17E-01
Thermogymnomonas_A	Desulfonispora_B	8.16E-01	9.48E-04	4.72E-02	2.68E-01
Enterorhabdus_B	Desulforegula_B	-9.17E-01	1.75E-05	1.30E-03	7.84E-03
Haloglycomyces_B	Desulforegula_B	1.00E+00	2.22E-16	2.21E-14	1.90E-06
Hespellia_B	Desulforegula_B	-9.70E-01	1.04E-07	9.77E-06	3.19E-04
Microscilla_B	Desulforegula_B	1.00E+00	2.22E-16	2.21E-14	1.90E-06
Petrimonas_B	Desulforegula_B	1.00E+00	2.22E-16	2.21E-14	1.90E-06

Porphyromonas_B	Desulforegula_B	1.00E+00	2.22E-16	2.21E-14	1.90E-06
Propionibacterium_B	Desulforegula_B	1.00E+00	2.22E-16	2.21E-14	1.90E-06
Pseudomonas_B	Desulforegula_B	1.00E+00	2.22E-16	2.21E-14	1.90E-06
Sanguibacter_B	Desulforegula_B	1.00E+00	2.22E-16	2.21E-14	1.90E-06
Shuttleworthia_B	Desulforegula_B	-8.88E-01	8.23E-05	5.41E-03	6.01E-02
Spirochaeta_B	Desulforegula_B	1.00E+00	2.22E-16	2.21E-14	1.90E-06
unclassified_F	Desulforegula_B	-8.44E-01	4.25E-04	2.37E-02	2.17E-01
Ganoderma_F	Desulfotomaculum_B	1.00E+00	2.22E-16	2.21E-14	1.90E-06
Geosmithia_putterillii_sensu_Pitt_F	Desulfotomaculum_B	-9.16E-01	1.90E-05	1.39E-03	1.77E-02
Johnsonella_B	Desulfotomaculum_B	1.00E+00	2.22E-16	2.21E-14	1.90E-06
Leptonema_B	Desulfotomaculum_B	1.00E+00	2.22E-16	2.21E-14	1.90E-06
Lutimonas_B	Desulfotomaculum_B	1.00E+00	2.22E-16	2.21E-14	1.90E-06
Massilia_B	Desulfotomaculum_B	1.00E+00	2.22E-16	2.21E-14	1.90E-06
Parasegetibacter_B	Desulfotomaculum_B	-9.42E-01	2.86E-06	2.46E-04	7.84E-03
Schwartzia_B	Desulfotomaculum_B	-9.26E-01	9.83E-06	7.85E-04	7.84E-03
Tepidanaerobacter_B	Desulfotomaculum_B	1.00E+00	2.22E-16	2.21E-14	1.90E-06
Thermoflavimicrobium_B	Desulfotomaculum_B	1.00E+00	2.22E-16	2.21E-14	1.90E-06
Unc_Verticillium_F	Desulfotomaculum_B	1.00E+00	2.22E-16	2.21E-14	1.90E-06
Prolixibacter_B	Desulfovibrio_B	-9.86E-01	1.64E-09	1.60E-07	2.89E-05
Rhodothermus_B	Desulfovibrio_B	1.00E+00	2.22E-16	2.21E-14	1.90E-06
Penicilliopsis_F	Desulfurispora_B	1.00E+00	2.22E-16	2.21E-14	1.90E-06
Planococcus_B	Desulfurispora_B	1.00E+00	2.22E-16	2.21E-14	1.90E-06
Protomyces_F	Dethiosulfatibacter_B	8.36E-01	5.31E-04	2.89E-02	2.17E-01
Mogibacterium_B	Dethiosulfovibrio_B	-8.39E-01	4.91E-04	2.70E-02	2.17E-01
Effluviibacter_B	Dolosigranulum_B	-8.27E-01	6.99E-04	3.65E-02	2.68E-01
Kordia_B	Dolosigranulum_B	1.00E+00	2.22E-16	2.21E-14	1.90E-06
Labedella_B	Dolosigranulum_B	1.00E+00	2.22E-16	2.21E-14	1.90E-06
Nicoletella_B	Dolosigranulum_B	1.00E+00	2.22E-16	2.21E-14	1.90E-06
Okibacterium_B	Dolosigranulum_B	1.00E+00	2.22E-16	2.21E-14	1.90E-06
Olsenella_B	Dolosigranulum_B	-9.47E-01	1.78E-06	1.57E-04	2.81E-03

Parascardovia_B	Dolosigranulum_B	1.00E+00	2.22E-16	2.21E-14	1.90E-06
Rhodovarius_B	Dolosigranulum_B	1.00E+00	2.22E-16	2.21E-14	1.90E-06
Slackia_B	Dolosigranulum_B	-9.07E-01	3.20E-05	2.25E-03	4.17E-02
Thermovenabulum_B	Dolosigranulum_B	1.00E+00	2.22E-16	2.21E-14	1.90E-06
Proteiniborus_B	Dorea_B	8.23E-01	7.79E-04	4.00E-02	2.68E-01
Treponema_B	Dothideomycetes_F [3]	-8.60E-01	2.42E-04	1.42E-02	2.17E-01
Frigoribacterium_B	Duganella_B	-9.86E-01	2.08E-09	2.03E-07	2.89E-05
Fluviicola_B	Echinicola_B	8.56E-01	2.86E-04	1.65E-02	2.17E-01
Jonquetella_B	Echinicola_B	-8.40E-01	4.73E-04	2.61E-02	2.17E-01
Lutaonella_B	Echinicola_B	8.91E-01	7.15E-05	4.76E-03	6.01E-02
Kordia_B	Effluviibacter_B	-8.27E-01	6.99E-04	3.65E-02	2.68E-01
Labedella_B	Effluviibacter_B	-8.27E-01	6.99E-04	3.65E-02	2.68E-01
Nicoletella_B	Effluviibacter_B	-8.27E-01	6.99E-04	3.65E-02	2.68E-01
Okibacterium_B	Effluviibacter_B	-8.27E-01	6.99E-04	3.65E-02	2.68E-01
Parascardovia_B	Effluviibacter_B	-8.27E-01	6.99E-04	3.65E-02	2.68E-01
Rhodovarius_B	Effluviibacter_B	-8.27E-01	6.99E-04	3.65E-02	2.68E-01
Thermovenabulum_B	Effluviibacter_B	-8.27E-01	6.99E-04	3.65E-02	2.68E-01
Xylanibacter_B	Eggerthella_B	-8.78E-01	1.25E-04	7.86E-03	6.06E-02
Fastidiosipila_B	Enterorhabdus_B	8.34E-01	5.73E-04	3.08E-02	2.17E-01
Haloglycomyces_B	Enterorhabdus_B	-9.17E-01	1.75E-05	1.30E-03	7.84E-03
Hespellia_B	Enterorhabdus_B	9.14E-01	2.21E-05	1.59E-03	1.77E-02
Microscilla_B	Enterorhabdus_B	-9.17E-01	1.75E-05	1.30E-03	7.84E-03
Petrimonas_B	Enterorhabdus_B	-9.17E-01	1.75E-05	1.30E-03	7.84E-03
Porphyromonas_B	Enterorhabdus_B	-9.17E-01	1.75E-05	1.30E-03	7.84E-03
Propionibacterium_B	Enterorhabdus_B	-9.17E-01	1.75E-05	1.30E-03	7.84E-03
Pseudomonas_B	Enterorhabdus_B	-9.17E-01	1.75E-05	1.30E-03	7.84E-03
Robinsoniella_B	Enterorhabdus_B	8.32E-01	6.07E-04	3.24E-02	2.17E-01
Sanguibacter_B	Enterorhabdus_B	-9.17E-01	1.75E-05	1.30E-03	7.84E-03
Shuttleworthia_B	Enterorhabdus_B	8.91E-01	7.24E-05	4.81E-03	6.01E-02
Spirochaeta_B	Enterorhabdus_B	-9.17E-01	1.75E-05	1.30E-03	7.84E-03

Syntrophococcus_B	Enterorhabdus_B	8.21E-01	8.16E-04	4.16E-02	2.68E-01
Turicibacter_B	Enterorhabdus_B	-8.66E-01	2.00E-04	1.19E-02	1.04E-01
Fibrobacter_B	Eremococcus_B	-8.30E-01	6.44E-04	3.40E-02	2.17E-01
Flammeovirga_B	Eremococcus_B	1.00E+00	2.22E-16	2.21E-14	1.90E-06
Gracilimonas_B	Eremococcus_B	1.00E+00	2.22E-16	2.21E-14	1.90E-06
Halogeometricum_A	Eremococcus_B	1.00E+00	2.22E-16	2.21E-14	1.90E-06
Hydrogenobaculum_A	Eremococcus_B	1.00E+00	2.22E-16	2.21E-14	1.90E-06
Myceligenerans_B	Eremococcus_B	1.00E+00	2.22E-16	2.21E-14	1.90E-06
Otidea_F	Eremococcus_B	1.00E+00	2.22E-16	2.21E-14	1.90E-06
Quatrionicoccus_B	Eremococcus_B	1.00E+00	2.22E-16	2.21E-14	1.90E-06
Roseospirillum_B	Eremococcus_B	1.00E+00	2.22E-16	2.21E-14	1.90E-06
Thermodesulfobium_B	Eremococcus_B	1.00E+00	2.22E-16	2.21E-14	1.90E-06
Yaniella_B	Eremococcus_B	1.00E+00	2.22E-16	2.21E-14	1.90E-06
Filimonas_B	Erysipelothrix_B	-9.29E-01	8.34E-06	6.77E-04	7.84E-03
Kineococcus_B	Erysipelothrix_B	-9.29E-01	8.34E-06	6.77E-04	7.84E-03
Nesiotobacter_B	Erysipelothrix_B	-9.29E-01	8.34E-06	6.77E-04	7.84E-03
Oxalicibacterium_B	Erysipelothrix_B	-9.29E-01	8.34E-06	6.77E-04	7.84E-03
Paracoccus_B	Erysipelothrix_B	-9.29E-01	8.34E-06	6.77E-04	7.84E-03
Paralactobacillus_B	Erysipelothrix_B	-9.29E-01	8.34E-06	6.77E-04	7.84E-03
Proteiniphilum_B	Erysipelothrix_B	-9.29E-01	8.34E-06	6.77E-04	7.84E-03
Psychrobacter_B	Erysipelothrix_B	-9.29E-01	8.34E-06	6.77E-04	7.84E-03
Sediminibacterium_B	Erysipelothrix_B	-9.29E-01	8.34E-06	6.77E-04	7.84E-03
Sediminicola_B	Erysipelothrix_B	-8.18E-01	8.98E-04	4.51E-02	2.68E-01
Selenomonas_B	Erysipelothrix_B	-9.29E-01	8.34E-06	6.77E-04	7.84E-03
Stenoxybacter_B	Erysipelothrix_B	-9.29E-01	8.34E-06	6.77E-04	7.84E-03
Talaromyces_F	Erysipelothrix_B	-9.29E-01	8.34E-06	6.77E-04	7.84E-03
unclassified_Pezizomycotina_F	Erysipelothrix_B	9.29E-01	8.34E-06	6.77E-04	7.84E-03
Howardella_B	Ethanoligenens_B	8.57E-01	2.74E-04	1.59E-02	2.17E-01
Hypocrea_F	Ethanoligenens_B	8.46E-01	3.92E-04	2.21E-02	2.17E-01
Thermogymnomonas_A	Ethanoligenens_B	8.93E-01	6.56E-05	4.39E-03	6.01E-02

unclassified_B	Ethanoligenens_B	8.64E-01	2.15E-04	1.27E-02	1.04E-01
Hespellia_B	Eubacterium_B	8.71E-01	1.63E-04	9.92E-03	1.04E-01
Oribacterium_B	Eubacterium_B	8.25E-01	7.37E-04	3.82E-02	2.68E-01
Faecalibacterium_B	Exilispira_B	8.15E-01	9.64E-04	4.78E-02	2.68E-01
Lactovum_B	Exilispira_B	9.80E-01	1.12E-08	1.09E-06	2.87E-04
Halalkalibacillus_B	Fabibacter_B	-9.60E-01	4.49E-07	4.13E-05	2.21E-03
Zhangella_B	Fabibacter_B	-9.05E-01	3.57E-05	2.49E-03	4.17E-02
Galbibacter_B	Facklamia_B	1.00E+00	2.22E-16	2.21E-14	1.90E-06
Lacticigenium_B	Facklamia_B	1.00E+00	2.22E-16	2.21E-14	1.90E-06
Methylobacterium_B	Facklamia_B	1.00E+00	2.22E-16	2.21E-14	1.90E-06
Ornithinimicrobium_B	Facklamia_B	1.00E+00	2.22E-16	2.21E-14	1.90E-06
Oxalophagus_B	Facklamia_B	-9.54E-01	8.65E-07	7.82E-05	2.81E-03
Pseudozobellia_B	Facklamia_B	-9.16E-01	1.90E-05	1.39E-03	1.77E-02
Rhodopila_B	Facklamia_B	1.00E+00	2.22E-16	2.21E-14	1.90E-06
Streptophyta_B	Facklamia_B	-9.16E-01	1.90E-05	1.39E-03	1.77E-02
Trichococcus_B	Facklamia_B	1.00E+00	2.22E-16	2.21E-14	1.90E-06
Hydrogenoanaerobacterium_B	Faecalibacterium_B	8.43E-01	4.31E-04	2.40E-02	2.17E-01
Oscillibacter_B	Fastidiosipila_B	9.12E-01	2.39E-05	1.71E-03	1.77E-02
Persicitalea_B	Fastidiosipila_B	-8.80E-01	1.15E-04	7.30E-03	6.06E-02
Solobacterium_B	Fastidiosipila_B	8.37E-01	5.28E-04	2.87E-02	2.17E-01
Xylanibacter_B	Fastidiosipila_B	8.53E-01	3.15E-04	1.81E-02	2.17E-01
Flammeovirga_B	Fibrobacter_B	-8.30E-01	6.44E-04	3.40E-02	2.17E-01
Gracilimonas_B	Fibrobacter_B	-8.30E-01	6.44E-04	3.40E-02	2.17E-01
Halogeometricum_A	Fibrobacter_B	-8.30E-01	6.44E-04	3.40E-02	2.17E-01
Hydrogenobaculum_A	Fibrobacter_B	-8.30E-01	6.44E-04	3.40E-02	2.17E-01
Myceligenerans_B	Fibrobacter_B	-8.30E-01	6.44E-04	3.40E-02	2.17E-01
Otidea_F	Fibrobacter_B	-8.30E-01	6.44E-04	3.40E-02	2.17E-01
Quatrionicoccus_B	Fibrobacter_B	-8.30E-01	6.44E-04	3.40E-02	2.17E-01
Roseospirillum_B	Fibrobacter_B	-8.30E-01	6.44E-04	3.40E-02	2.17E-01
Thermodesulfobium_B	Fibrobacter_B	-8.30E-01	6.44E-04	3.40E-02	2.17E-01

Yaniella_B	Fibrobacter_B	-8.30E-01	6.44E-04	3.40E-02	2.17E-01
Kineococcus_B	Filimonas_B	1.00E+00	2.22E-16	2.21E-14	1.90E-06
Nesiotobacter_B	Filimonas_B	1.00E+00	2.22E-16	2.21E-14	1.90E-06
Nubsella_B	Filimonas_B	8.16E-01	9.31E-04	4.65E-02	2.68E-01
Oxalicibacterium_B	Filimonas_B	1.00E+00	2.22E-16	2.21E-14	1.90E-06
Paracoccus_B	Filimonas_B	1.00E+00	2.22E-16	2.21E-14	1.90E-06
Paralactobacillus_B	Filimonas_B	1.00E+00	2.22E-16	2.21E-14	1.90E-06
Proteiniphilum_B	Filimonas_B	1.00E+00	2.22E-16	2.21E-14	1.90E-06
Psychrobacter_B	Filimonas_B	1.00E+00	2.22E-16	2.21E-14	1.90E-06
Sediminibacterium_B	Filimonas_B	1.00E+00	2.22E-16	2.21E-14	1.90E-06
Selenomonas_B	Filimonas_B	1.00E+00	2.22E-16	2.21E-14	1.90E-06
Stenoxybacter_B	Filimonas_B	1.00E+00	2.22E-16	2.21E-14	1.90E-06
Talaromyces_F	Filimonas_B	1.00E+00	2.22E-16	2.21E-14	1.90E-06
unclassified_Pezizomycotina_F	Filimonas_B	-1.00E+00	2.22E-16	2.21E-14	1.90E-06
Phocaeicola_B	Filobacillus_B	8.43E-01	4.36E-04	2.43E-02	2.17E-01
Gemmatimonas_B	Filobasidium_F	-9.68E-01	1.35E-07	1.26E-05	5.68E-04
Geosporobacter_B	Filobasidium_F	9.57E-01	6.20E-07	5.66E-05	2.21E-03
Nocardioides_B	Filobasidium_F	-9.68E-01	1.35E-07	1.26E-05	5.68E-04
Olivibacter_B	Filobasidium_F	8.67E-01	1.90E-04	1.14E-02	1.04E-01
Pelomonas_B	Filobasidium_F	-9.68E-01	1.35E-07	1.26E-05	5.68E-04
Unc_Kirschsteiniothelia_F	Filobasidium_F	9.68E-01	1.35E-07	1.26E-05	5.68E-04
Verrucomicrobium_B	Filobasidium_F	8.63E-01	2.23E-04	1.31E-02	2.17E-01
Garciella_B	Finegoldia_B	8.20E-01	8.41E-04	4.27E-02	2.68E-01
Persicitalea_B	Finegoldia_B	-8.36E-01	5.41E-04	2.93E-02	2.17E-01
Gracilimonas_B	Flammeovirga_B	1.00E+00	2.22E-16	2.21E-14	1.90E-06
Halogeometricum_A	Flammeovirga_B	1.00E+00	2.22E-16	2.21E-14	1.90E-06
Hydrogenobaculum_A	Flammeovirga_B	1.00E+00	2.22E-16	2.21E-14	1.90E-06
Myceligenerans_B	Flammeovirga_B	1.00E+00	2.22E-16	2.21E-14	1.90E-06
Otidea_F	Flammeovirga_B	1.00E+00	2.22E-16	2.21E-14	1.90E-06
Quatrionicoccus_B	Flammeovirga_B	1.00E+00	2.22E-16	2.21E-14	1.90E-06

Roseospirillum_B	Flammeovirga_B	1.00E+00	2.22E-16	2.21E-14	1.90E-06
Thermodesulfobium_B	Flammeovirga_B	1.00E+00	2.22E-16	2.21E-14	1.90E-06
Yaniella_B	Flammeovirga_B	1.00E+00	2.22E-16	2.21E-14	1.90E-06
Lutaonella_B	Fluviicola_B	8.58E-01	2.62E-04	1.53E-02	2.17E-01
Fulvivirga_B	Fulvimonas_B	1.00E+00	2.22E-16	2.21E-14	1.90E-06
Jonesia_B	Fulvimonas_B	1.00E+00	2.22E-16	2.21E-14	1.90E-06
Lishizhenia_B	Fulvimonas_B	1.00E+00	2.22E-16	2.21E-14	1.90E-06
Ornithobacterium_B	Fulvimonas_B	-9.11E-01	2.50E-05	1.78E-03	3.13E-02
Parachlamydia_B	Fulvimonas_B	1.00E+00	2.22E-16	2.21E-14	1.90E-06
Sphaerobacter_B	Fulvimonas_B	1.00E+00	2.22E-16	2.21E-14	1.90E-06
Sporanaerobacter_B	Fulvimonas_B	1.00E+00	2.22E-16	2.21E-14	1.90E-06
Unc_Kockovaella_F	Fulvimonas_B	1.00E+00	2.22E-16	2.21E-14	1.90E-06
Venenivibrio_B	Fulvimonas_B	1.00E+00	2.22E-16	2.21E-14	1.90E-06
Zunongwangia_B	Fulvimonas_B	1.00E+00	2.22E-16	2.21E-14	1.90E-06
Jonesia_B	Fulvivirga_B	1.00E+00	2.22E-16	2.21E-14	1.90E-06
Lishizhenia_B	Fulvivirga_B	1.00E+00	2.22E-16	2.21E-14	1.90E-06
Ornithobacterium_B	Fulvivirga_B	-9.11E-01	2.50E-05	1.78E-03	3.13E-02
Parachlamydia_B	Fulvivirga_B	1.00E+00	2.22E-16	2.21E-14	1.90E-06
Sphaerobacter_B	Fulvivirga_B	1.00E+00	2.22E-16	2.21E-14	1.90E-06
Sporanaerobacter_B	Fulvivirga_B	1.00E+00	2.22E-16	2.21E-14	1.90E-06
Unc_Kockovaella_F	Fulvivirga_B	1.00E+00	2.22E-16	2.21E-14	1.90E-06
Venenivibrio_B	Fulvivirga_B	1.00E+00	2.22E-16	2.21E-14	1.90E-06
Zunongwangia_B	Fulvivirga_B	1.00E+00	2.22E-16	2.21E-14	1.90E-06
Hallella_B	Fusibacter_B	8.92E-01	6.75E-05	4.51E-03	6.01E-02
Parvimonas_B	Fusibacter_B	-9.47E-01	1.86E-06	1.63E-04	4.74E-03
Ruminococcus_B	Fusibacter_B	8.26E-01	7.17E-04	3.73E-02	2.68E-01
Scytinostroma_F	Fusibacter_B	1.00E+00	2.22E-16	2.21E-14	1.90E-06
Lacticigenium_B	Galbibacter_B	1.00E+00	2.22E-16	2.21E-14	1.90E-06
Methylobacterium_B	Galbibacter_B	1.00E+00	2.22E-16	2.21E-14	1.90E-06
Ornithinimicrobium_B	Galbibacter_B	1.00E+00	2.22E-16	2.21E-14	1.90E-06

Oxalophagus_B	Galbibacter_B	-9.54E-01	8.65E-07	7.82E-05	2.81E-03
Pseudozobellia_B	Galbibacter_B	-9.16E-01	1.90E-05	1.39E-03	1.77E-02
Rhodopila_B	Galbibacter_B	1.00E+00	2.22E-16	2.21E-14	1.90E-06
Streptophyta_B	Galbibacter_B	-9.16E-01	1.90E-05	1.39E-03	1.77E-02
Trichococcus_B	Galbibacter_B	1.00E+00	2.22E-16	2.21E-14	1.90E-06
Geosmithia_putterillii_sensu_Pitt_F	Ganoderma_F	-9.16E-01	1.90E-05	1.39E-03	1.77E-02
Johnsonella_B	Ganoderma_F	1.00E+00	2.22E-16	2.21E-14	1.90E-06
Leptonema_B	Ganoderma_F	1.00E+00	2.22E-16	2.21E-14	1.90E-06
Lutimonas_B	Ganoderma_F	1.00E+00	2.22E-16	2.21E-14	1.90E-06
Massilia_B	Ganoderma_F	1.00E+00	2.22E-16	2.21E-14	1.90E-06
Parasegetibacter_B	Ganoderma_F	-9.42E-01	2.86E-06	2.46E-04	7.84E-03
Schwartzia_B	Ganoderma_F	-9.26E-01	9.83E-06	7.85E-04	7.84E-03
Tepidanaerobacter_B	Ganoderma_F	1.00E+00	2.22E-16	2.21E-14	1.90E-06
Thermoflavimicrobium_B	Ganoderma_F	1.00E+00	2.22E-16	2.21E-14	1.90E-06
Unc_Verticillium_F	Ganoderma_F	1.00E+00	2.22E-16	2.21E-14	1.90E-06
Kiloniella_B	Garciella_B	-8.42E-01	4.44E-04	2.47E-02	2.17E-01
Oribacterium_B	Garciella_B	8.34E-01	5.64E-04	3.04E-02	2.17E-01
Pelospora_B	Garciella_B	8.23E-01	7.84E-04	4.03E-02	2.68E-01
Proteiniborus_B	Garciella_B	8.24E-01	7.66E-04	3.95E-02	2.68E-01
Stenotrophomonas_B	Garciella_B	-8.42E-01	4.44E-04	2.47E-02	2.17E-01
Geosporobacter_B	Gemmatimonas_B	-9.12E-01	2.43E-05	1.74E-03	1.77E-02
Nocardioides_B	Gemmatimonas_B	1.00E+00	2.22E-16	2.21E-14	1.90E-06
Olivibacter_B	Gemmatimonas_B	-9.17E-01	1.75E-05	1.30E-03	7.84E-03
Pelomonas_B	Gemmatimonas_B	1.00E+00	2.22E-16	2.21E-14	1.90E-06
Unc_Kirschsteiniothelia_F	Gemmatimonas_B	-1.00E+00	2.22E-16	2.21E-14	1.90E-06
Verrucomicrobium_B	Gemmatimonas_B	-8.93E-01	6.47E-05	4.33E-03	6.01E-02
Leucobacter_B	Geopsychrobacter_B	-9.68E-01	1.43E-07	1.33E-05	1.35E-03
Wolinella_B	Geopsychrobacter_B	8.45E-01	4.04E-04	2.27E-02	2.17E-01
Johnsonella_B	Geosmithia_putterillii_sensu_Pitt_F	-9.16E-01	1.90E-05	1.39E-03	1.77E-02
Kaistella_B	Geosmithia_putterillii_sensu_Pitt_F	8.35E-01	5.53E-04	2.99E-02	2.17E-01

Leptonema_B	Geosmithia_putterillii_sensu_Pitt_F	-9.16E-01	1.90E-05	1.39E-03	1.77E-02
Lutimonas_B	Geosmithia_putterillii_sensu_Pitt_F	-9.16E-01	1.90E-05	1.39E-03	1.77E-02
Massilia_B	Geosmithia_putterillii_sensu_Pitt_F	-9.16E-01	1.90E-05	1.39E-03	1.77E-02
Parasegetibacter_B	Geosmithia_putterillii_sensu_Pitt_F	8.77E-01	1.27E-04	7.96E-03	6.06E-02
Schwartzia_B	Geosmithia_putterillii_sensu_Pitt_F	9.57E-01	6.28E-07	5.73E-05	2.21E-03
Tepidanaerobacter_B	Geosmithia_putterillii_sensu_Pitt_F	-9.16E-01	1.90E-05	1.39E-03	1.77E-02
Thermoflavimicrobium_B	Geosmithia_putterillii_sensu_Pitt_F	-9.16E-01	1.90E-05	1.39E-03	1.77E-02
Unc_Verticillium_F	Geosmithia_putterillii_sensu_Pitt_F	-9.16E-01	1.90E-05	1.39E-03	1.77E-02
Nocardioides_B	Geosporobacter_B	-9.12E-01	2.43E-05	1.74E-03	1.77E-02
Pelomonas_B	Geosporobacter_B	-9.12E-01	2.43E-05	1.74E-03	1.77E-02
Unc_Kirschsteiniothelia_F	Geosporobacter_B	9.12E-01	2.43E-05	1.74E-03	1.77E-02
Verrucomicrobium_B	Geosporobacter_B	8.34E-01	5.68E-04	3.06E-02	2.17E-01
Subdoligranulum_B	Globicatella_B	-8.37E-01	5.25E-04	2.86E-02	2.17E-01
Halogeometricum_A	Gracilimonas_B	1.00E+00	2.22E-16	2.21E-14	1.90E-06
Hydrogenobaculum_A	Gracilimonas_B	1.00E+00	2.22E-16	2.21E-14	1.90E-06
Myceligenerans_B	Gracilimonas_B	1.00E+00	2.22E-16	2.21E-14	1.90E-06
Otidea_F	Gracilimonas_B	1.00E+00	2.22E-16	2.21E-14	1.90E-06
Quatrionicoccus_B	Gracilimonas_B	1.00E+00	2.22E-16	2.21E-14	1.90E-06
Roseospirillum_B	Gracilimonas_B	1.00E+00	2.22E-16	2.21E-14	1.90E-06
Thermodesulfobium_B	Gracilimonas_B	1.00E+00	2.22E-16	2.21E-14	1.90E-06
Yaniella_B	Gracilimonas_B	1.00E+00	2.22E-16	2.21E-14	1.90E-06
Lentisphaera_B	Guggenheimella_B	-9.72E-01	6.40E-08	6.11E-06	2.87E-04
Sedimentibacter_B	Guggenheimella_B	9.27E-01	9.35E-06	7.50E-04	7.84E-03
unclassified_B	Guggenheimella_B	8.94E-01	6.17E-05	4.15E-03	6.01E-02
Xylanibacter_B	Guggenheimella_B	8.70E-01	1.68E-04	1.02E-02	1.04E-01
Larkinella_B	Haliscomenobacter_B	-8.36E-01	5.41E-04	2.93E-02	2.17E-01
Parabacteroides_B	Haliscomenobacter_B	8.36E-01	5.41E-04	2.93E-02	2.17E-01
Sporobacter_B	Haliscomenobacter_B	-8.94E-01	6.29E-05	4.22E-03	6.01E-02
Howardella_B	Hallella_B	8.79E-01	1.20E-04	7.57E-03	6.06E-02
Pseudobutyrivibrio_B	Hallella_B	8.28E-01	6.76E-04	3.55E-02	2.17E-01

Ruminococcus_B	Hallella_B	9.21E-01	1.36E-05	1.05E-03	7.84E-03
Scytinostroma_F	Hallella_B	8.92E-01	6.75E-05	4.51E-03	6.01E-02
Thermogymnomonas_A	Hallella_B	8.43E-01	4.40E-04	2.45E-02	2.17E-01
Hydrogenobaculum_A	Halogeometricum_A	1.00E+00	2.22E-16	2.21E-14	1.90E-06
Myceligenerans_B	Halogeometricum_A	1.00E+00	2.22E-16	2.21E-14	1.90E-06
Otidea_F	Halogeometricum_A	1.00E+00	2.22E-16	2.21E-14	1.90E-06
Quatrionicoccus_B	Halogeometricum_A	1.00E+00	2.22E-16	2.21E-14	1.90E-06
Roseospirillum_B	Halogeometricum_A	1.00E+00	2.22E-16	2.21E-14	1.90E-06
Thermodesulfobium_B	Halogeometricum_A	1.00E+00	2.22E-16	2.21E-14	1.90E-06
Yaniella_B	Halogeometricum_A	1.00E+00	2.22E-16	2.21E-14	1.90E-06
Hespellia_B	Haloglycomyces_B	-9.70E-01	1.04E-07	9.77E-06	3.19E-04
Microscilla_B	Haloglycomyces_B	1.00E+00	2.22E-16	2.21E-14	1.90E-06
Petrimonas_B	Haloglycomyces_B	1.00E+00	2.22E-16	2.21E-14	1.90E-06
Porphyromonas_B	Haloglycomyces_B	1.00E+00	2.22E-16	2.21E-14	1.90E-06
Propionibacterium_B	Haloglycomyces_B	1.00E+00	2.22E-16	2.21E-14	1.90E-06
Pseudomonas_B	Haloglycomyces_B	1.00E+00	2.22E-16	2.21E-14	1.90E-06
Sanguibacter_B	Haloglycomyces_B	1.00E+00	2.22E-16	2.21E-14	1.90E-06
Shuttleworthia_B	Haloglycomyces_B	-8.88E-01	8.23E-05	5.41E-03	6.01E-02
Spirochaeta_B	Haloglycomyces_B	1.00E+00	2.22E-16	2.21E-14	1.90E-06
unclassified_F	Haloglycomyces_B	-8.44E-01	4.25E-04	2.37E-02	2.17E-01
Lactonifactor_B	Hespellia_B	8.43E-01	4.32E-04	2.41E-02	2.17E-01
Microscilla_B	Hespellia_B	-9.70E-01	1.04E-07	9.77E-06	3.19E-04
Petrimonas_B	Hespellia_B	-9.70E-01	1.04E-07	9.77E-06	3.19E-04
Porphyromonas_B	Hespellia_B	-9.70E-01	1.04E-07	9.77E-06	3.19E-04
Propionibacterium_B	Hespellia_B	-9.70E-01	1.04E-07	9.77E-06	3.19E-04
Pseudomonas_B	Hespellia_B	-9.70E-01	1.04E-07	9.77E-06	3.19E-04
Sanguibacter_B	Hespellia_B	-9.70E-01	1.04E-07	9.77E-06	3.19E-04
Shuttleworthia_B	Hespellia_B	8.76E-01	1.36E-04	8.47E-03	7.38E-02
Spirochaeta_B	Hespellia_B	-9.70E-01	1.04E-07	9.77E-06	3.19E-04
Syntrophococcus_B	Hespellia_B	8.25E-01	7.42E-04	3.84E-02	2.68E-01

Turicibacter_B	Hippea_B	-8.20E-01	8.54E-04	4.33E-02	2.68E-01
Leuconostoc_B	Holtermannia_F	1.00E+00	2.22E-16	2.21E-14	1.90E-06
Levilinea_B	Holtermannia_F	-9.17E-01	1.75E-05	1.30E-03	7.84E-03
Parasporobacterium_B	Holtermannia_F	8.63E-01	2.19E-04	1.29E-02	2.17E-01
Phascolarctobacterium_B	Holtermannia_F	1.00E+00	2.22E-16	2.21E-14	1.90E-06
Pirellula_B	Holtermannia_F	1.00E+00	2.22E-16	2.21E-14	1.90E-06
Rhodopirellula_B	Holtermannia_F	-8.76E-01	1.33E-04	8.28E-03	7.38E-02
Rudanella_B	Holtermannia_F	1.00E+00	2.22E-16	2.21E-14	1.90E-06
Thermonema_B	Holtermannia_F	1.00E+00	2.22E-16	2.21E-14	1.90E-06
Tropheryma_B	Holtermannia_F	1.00E+00	2.22E-16	2.21E-14	1.90E-06
Unc_Cryptococcus_F	Holtermannia_F	1.00E+00	2.22E-16	2.21E-14	1.90E-06
Verminephrobacter_B	Holtermannia_F	1.00E+00	2.22E-16	2.21E-14	1.90E-06
Ruminococcus_B	Howardella_B	8.45E-01	4.02E-04	2.26E-02	2.17E-01
Thermogymnomonas_A	Howardella_B	9.77E-01	2.47E-08	2.39E-06	2.87E-04
Succiniclasticum_B	Hydrogenoanaerobacterium_B	8.56E-01	2.79E-04	1.62E-02	2.17E-01
Myceligenerans_B	Hydrogenobaculum_A	1.00E+00	2.22E-16	2.21E-14	1.90E-06
Otidea_F	Hydrogenobaculum_A	1.00E+00	2.22E-16	2.21E-14	1.90E-06
Quatrionicoccus_B	Hydrogenobaculum_A	1.00E+00	2.22E-16	2.21E-14	1.90E-06
Roseospirillum_B	Hydrogenobaculum_A	1.00E+00	2.22E-16	2.21E-14	1.90E-06
Thermodesulfobium_B	Hydrogenobaculum_A	1.00E+00	2.22E-16	2.21E-14	1.90E-06
Yaniella_B	Hydrogenobaculum_A	1.00E+00	2.22E-16	2.21E-14	1.90E-06
Lycoperdon_F	Hypocrea_F	8.13E-01	1.01E-03	4.95E-02	2.68E-01
Schlesneria_B	Isobaculum_B	-9.54E-01	8.47E-07	7.66E-05	2.21E-03
Leptonema_B	Johnsonella_B	1.00E+00	2.22E-16	2.21E-14	1.90E-06
Lutimonas_B	Johnsonella_B	1.00E+00	2.22E-16	2.21E-14	1.90E-06
Massilia_B	Johnsonella_B	1.00E+00	2.22E-16	2.21E-14	1.90E-06
Parasegetibacter_B	Johnsonella_B	-9.42E-01	2.86E-06	2.46E-04	7.84E-03
Schwartzia_B	Johnsonella_B	-9.26E-01	9.83E-06	7.85E-04	7.84E-03
Tepidanaerobacter_B	Johnsonella_B	1.00E+00	2.22E-16	2.21E-14	1.90E-06
Thermoflavimicrobium_B	Johnsonella_B	1.00E+00	2.22E-16	2.21E-14	1.90E-06

Unc_Verticillium_F	Johnsonella_B	1.00E+00	2.22E-16	2.21E-14	1.90E-06
Lishizhenia_B	Jonesia_B	1.00E+00	2.22E-16	2.21E-14	1.90E-06
Ornithobacterium_B	Jonesia_B	-9.11E-01	2.50E-05	1.78E-03	3.13E-02
Parachlamydia_B	Jonesia_B	1.00E+00	2.22E-16	2.21E-14	1.90E-06
Sphaerobacter_B	Jonesia_B	1.00E+00	2.22E-16	2.21E-14	1.90E-06
Sporanaerobacter_B	Jonesia_B	1.00E+00	2.22E-16	2.21E-14	1.90E-06
Unc_Kockovaella_F	Jonesia_B	1.00E+00	2.22E-16	2.21E-14	1.90E-06
Venenivibrio_B	Jonesia_B	1.00E+00	2.22E-16	2.21E-14	1.90E-06
Zunongwangia_B	Jonesia_B	1.00E+00	2.22E-16	2.21E-14	1.90E-06
Pedobacter_B	Jonquetella_B	-8.30E-01	6.37E-04	3.37E-02	2.17E-01
unclassified_F	Jonquetella_B	8.93E-01	6.38E-05	4.28E-03	6.01E-02
Peptococcus_B	Kaistella_B	8.41E-01	4.61E-04	2.55E-02	2.17E-01
Oribacterium_B	Kiloniella_B	-9.07E-01	3.17E-05	2.22E-03	3.13E-02
Pelospora_B	Kiloniella_B	-9.38E-01	3.98E-06	3.38E-04	7.84E-03
Sporobacterium_B	Kiloniella_B	-9.77E-01	2.32E-08	2.25E-06	2.87E-04
Stenotrophomonas_B	Kiloniella_B	1.00E+00	2.22E-16	2.21E-14	1.90E-06
Nesiotobacter_B	Kineococcus_B	1.00E+00	2.22E-16	2.21E-14	1.90E-06
Nubsella_B	Kineococcus_B	8.16E-01	9.31E-04	4.65E-02	2.68E-01
Oxalicibacterium_B	Kineococcus_B	1.00E+00	2.22E-16	2.21E-14	1.90E-06
Paracoccus_B	Kineococcus_B	1.00E+00	2.22E-16	2.21E-14	1.90E-06
Paralactobacillus_B	Kineococcus_B	1.00E+00	2.22E-16	2.21E-14	1.90E-06
Proteiniphilum_B	Kineococcus_B	1.00E+00	2.22E-16	2.21E-14	1.90E-06
Psychrobacter_B	Kineococcus_B	1.00E+00	2.22E-16	2.21E-14	1.90E-06
Sediminibacterium_B	Kineococcus_B	1.00E+00	2.22E-16	2.21E-14	1.90E-06
Selenomonas_B	Kineococcus_B	1.00E+00	2.22E-16	2.21E-14	1.90E-06
Stenoxybacter_B	Kineococcus_B	1.00E+00	2.22E-16	2.21E-14	1.90E-06
Talaromyces_F	Kineococcus_B	1.00E+00	2.22E-16	2.21E-14	1.90E-06
unclassified_Pezizomycotina_F	Kineococcus_B	-1.00E+00	2.22E-16	2.21E-14	1.90E-06
Labedella_B	Kordia_B	1.00E+00	2.22E-16	2.21E-14	1.90E-06
Nicoletella_B	Kordia_B	1.00E+00	2.22E-16	2.21E-14	1.90E-06

Okibacterium_B	Kordia_B	1.00E+00	2.22E-16	2.21E-14	1.90E-06
Olsenella_B	Kordia_B	-9.47E-01	1.78E-06	1.57E-04	2.81E-03
Parascardovia_B	Kordia_B	1.00E+00	2.22E-16	2.21E-14	1.90E-06
Rhodovarius_B	Kordia_B	1.00E+00	2.22E-16	2.21E-14	1.90E-06
Slackia_B	Kordia_B	-9.07E-01	3.20E-05	2.25E-03	4.17E-02
Thermovenabulum_B	Kordia_B	1.00E+00	2.22E-16	2.21E-14	1.90E-06
Shuttleworthia_B	Kosmotoga_B	-8.48E-01	3.74E-04	2.12E-02	2.17E-01
Nicoletella_B	Labedella_B	1.00E+00	2.22E-16	2.21E-14	1.90E-06
Okibacterium_B	Labedella_B	1.00E+00	2.22E-16	2.21E-14	1.90E-06
Olsenella_B	Labedella_B	-9.47E-01	1.78E-06	1.57E-04	2.81E-03
Parascardovia_B	Labedella_B	1.00E+00	2.22E-16	2.21E-14	1.90E-06
Rhodovarius_B	Labedella_B	1.00E+00	2.22E-16	2.21E-14	1.90E-06
Slackia_B	Labedella_B	-9.07E-01	3.20E-05	2.25E-03	4.17E-02
Thermovenabulum_B	Labedella_B	1.00E+00	2.22E-16	2.21E-14	1.90E-06
Methylobacterium_B	Lacticigenium_B	1.00E+00	2.22E-16	2.21E-14	1.90E-06
Ornithinimicrobium_B	Lacticigenium_B	1.00E+00	2.22E-16	2.21E-14	1.90E-06
Oxalophagus_B	Lacticigenium_B	-9.54E-01	8.65E-07	7.82E-05	2.81E-03
Pseudozobellia_B	Lacticigenium_B	-9.16E-01	1.90E-05	1.39E-03	1.77E-02
Rhodopila_B	Lacticigenium_B	1.00E+00	2.22E-16	2.21E-14	1.90E-06
Streptophyta_B	Lacticigenium_B	-9.16E-01	1.90E-05	1.39E-03	1.77E-02
Trichococcus_B	Lacticigenium_B	1.00E+00	2.22E-16	2.21E-14	1.90E-06
Papillibacter_B	Lactonifactor_B	8.77E-01	1.30E-04	8.14E-03	6.06E-02
Pseudosphingobacterium_B	Lactonifactor_B	8.42E-01	4.49E-04	2.49E-02	2.17E-01
Sharpea_B	Lactonifactor_B	9.33E-01	6.21E-06	5.15E-04	7.84E-03
Sporobacter_B	Larkinella_B	8.31E-01	6.19E-04	3.29E-02	2.17E-01
Leucobacter_B	Lawsonia_B	-8.32E-01	6.04E-04	3.22E-02	2.17E-01
Rapidithrix_B	Lawsonia_B	-9.50E-01	1.37E-06	1.22E-04	2.81E-03
Solibacillus_B	Lawsonia_B	-9.50E-01	1.37E-06	1.22E-04	2.81E-03
Microbacterium_B	Leifsonia_B	9.88E-01	1.05E-09	1.02E-07	2.81E-05
Sedimentibacter_B	Lentisphaera_B	-9.54E-01	8.47E-07	7.66E-05	2.21E-03

Xylanibacter_B	Lentisphaera_B	-8.20E-01	8.40E-04	4.26E-02	2.68E-01
Microbacterium_B	Leptobacterium_B	-8.50E-01	3.48E-04	1.98E-02	2.17E-01
Peptostreptococcus_B	Leptobacterium_B	8.95E-01	5.91E-05	3.98E-03	6.01E-02
Selenihalanaerobacter_B	Leptobacterium_B	-9.66E-01	1.81E-07	1.68E-05	1.35E-03
Lutimonas_B	Leptonema_B	1.00E+00	2.22E-16	2.21E-14	1.90E-06
Massilia_B	Leptonema_B	1.00E+00	2.22E-16	2.21E-14	1.90E-06
Parasegetibacter_B	Leptonema_B	-9.42E-01	2.86E-06	2.46E-04	7.84E-03
Schwartzia_B	Leptonema_B	-9.26E-01	9.83E-06	7.85E-04	7.84E-03
Tepidanaerobacter_B	Leptonema_B	1.00E+00	2.22E-16	2.21E-14	1.90E-06
Thermoflavimicrobium_B	Leptonema_B	1.00E+00	2.22E-16	2.21E-14	1.90E-06
Unc_Verticillium_F	Leptonema_B	1.00E+00	2.22E-16	2.21E-14	1.90E-06
Wolinella_B	Leucobacter_B	-8.46E-01	3.93E-04	2.21E-02	2.17E-01
Levilinea_B	Leuconostoc_B	-9.17E-01	1.75E-05	1.30E-03	7.84E-03
Parasporobacterium_B	Leuconostoc_B	8.63E-01	2.19E-04	1.29E-02	2.17E-01
Phascolarctobacterium_B	Leuconostoc_B	1.00E+00	2.22E-16	2.21E-14	1.90E-06
Pirellula_B	Leuconostoc_B	1.00E+00	2.22E-16	2.21E-14	1.90E-06
Rhodopirellula_B	Leuconostoc_B	-8.76E-01	1.33E-04	8.28E-03	7.38E-02
Rudanella_B	Leuconostoc_B	1.00E+00	2.22E-16	2.21E-14	1.90E-06
Thermonema_B	Leuconostoc_B	1.00E+00	2.22E-16	2.21E-14	1.90E-06
Tropheryma_B	Leuconostoc_B	1.00E+00	2.22E-16	2.21E-14	1.90E-06
Unc_Cryptococcus_F	Leuconostoc_B	1.00E+00	2.22E-16	2.21E-14	1.90E-06
Verminephrobacter_B	Leuconostoc_B	1.00E+00	2.22E-16	2.21E-14	1.90E-06
Phascolarctobacterium_B	Levilinea_B	-9.17E-01	1.75E-05	1.30E-03	7.84E-03
Pirellula_B	Levilinea_B	-9.17E-01	1.75E-05	1.30E-03	7.84E-03
Rudanella_B	Levilinea_B	-9.17E-01	1.75E-05	1.30E-03	7.84E-03
Thermonema_B	Levilinea_B	-9.17E-01	1.75E-05	1.30E-03	7.84E-03
Tropheryma_B	Levilinea_B	-9.17E-01	1.75E-05	1.30E-03	7.84E-03
Unc_Cryptococcus_F	Levilinea_B	-9.17E-01	1.75E-05	1.30E-03	7.84E-03
Verminephrobacter_B	Levilinea_B	-9.17E-01	1.75E-05	1.30E-03	7.84E-03
Persicitalea_B	Limibacter_B	-8.39E-01	4.90E-04	2.69E-02	2.17E-01

Ornithobacterium_B	Lishizhenia_B	-9.11E-01	2.50E-05	1.78E-03	3.13E-02
Parachlamydia_B	Lishizhenia_B	1.00E+00	2.22E-16	2.21E-14	1.90E-06
Sphaerobacter_B	Lishizhenia_B	1.00E+00	2.22E-16	2.21E-14	1.90E-06
Sporanaerobacter_B	Lishizhenia_B	1.00E+00	2.22E-16	2.21E-14	1.90E-06
Unc_Kockovaella_F	Lishizhenia_B	1.00E+00	2.22E-16	2.21E-14	1.90E-06
Venenivibrio_B	Lishizhenia_B	1.00E+00	2.22E-16	2.21E-14	1.90E-06
Zunongwangia_B	Lishizhenia_B	1.00E+00	2.22E-16	2.21E-14	1.90E-06
Massilia_B	Lutimonas_B	1.00E+00	2.22E-16	2.21E-14	1.90E-06
Parasegetibacter_B	Lutimonas_B	-9.42E-01	2.86E-06	2.46E-04	7.84E-03
Schwartzia_B	Lutimonas_B	-9.26E-01	9.83E-06	7.85E-04	7.84E-03
Tepidanaerobacter_B	Lutimonas_B	1.00E+00	2.22E-16	2.21E-14	1.90E-06
Thermoflavimicrobium_B	Lutimonas_B	1.00E+00	2.22E-16	2.21E-14	1.90E-06
Unc_Verticillium_F	Lutimonas_B	1.00E+00	2.22E-16	2.21E-14	1.90E-06
Marvinbryantia_B	Lycoperdon_F	9.24E-01	1.12E-05	8.81E-04	7.84E-03
unclassified_B	Lycoperdon_F	8.33E-01	5.90E-04	3.16E-02	2.17E-01
Thermicanus_B	Malonomonas_B	8.31E-01	6.29E-04	3.34E-02	2.17E-01
Parasegetibacter_B	Massilia_B	-9.42E-01	2.86E-06	2.46E-04	7.84E-03
Schwartzia_B	Massilia_B	-9.26E-01	9.83E-06	7.85E-04	7.84E-03
Tepidanaerobacter_B	Massilia_B	1.00E+00	2.22E-16	2.21E-14	1.90E-06
Thermoflavimicrobium_B	Massilia_B	1.00E+00	2.22E-16	2.21E-14	1.90E-06
Unc_Verticillium_F	Massilia_B	1.00E+00	2.22E-16	2.21E-14	1.90E-06
Terasakiella_B	Methanobrevibacter_A	-8.92E-01	6.90E-05	4.60E-03	6.01E-02
Ornithinimicrobium_B	Methylobacterium_B	1.00E+00	2.22E-16	2.21E-14	1.90E-06
Oxalophagus_B	Methylobacterium_B	-9.54E-01	8.65E-07	7.82E-05	2.81E-03
Pseudozobellia_B	Methylobacterium_B	-9.16E-01	1.90E-05	1.39E-03	1.77E-02
Rhodopila_B	Methylobacterium_B	1.00E+00	2.22E-16	2.21E-14	1.90E-06
Streptophyta_B	Methylobacterium_B	-9.16E-01	1.90E-05	1.39E-03	1.77E-02
Trichococcus_B	Methylobacterium_B	1.00E+00	2.22E-16	2.21E-14	1.90E-06
Peptostreptococcus_B	Microbacterium_B	-8.28E-01	6.70E-04	3.52E-02	2.17E-01
Selenihalanaerobacter_B	Microbacterium_B	8.36E-01	5.31E-04	2.89E-02	2.17E-01

Petrimonas_B	Microscilla_B	1.00E+00	2.22E-16	2.21E-14	1.90E-06
Porphyromonas_B	Microscilla_B	1.00E+00	2.22E-16	2.21E-14	1.90E-06
Propionibacterium_B	Microscilla_B	1.00E+00	2.22E-16	2.21E-14	1.90E-06
Pseudomonas_B	Microscilla_B	1.00E+00	2.22E-16	2.21E-14	1.90E-06
Sanguibacter_B	Microscilla_B	1.00E+00	2.22E-16	2.21E-14	1.90E-06
Shuttleworthia_B	Microscilla_B	-8.88E-01	8.23E-05	5.41E-03	6.01E-02
Spirochaeta_B	Microscilla_B	1.00E+00	2.22E-16	2.21E-14	1.90E-06
unclassified_F	Microscilla_B	-8.44E-01	4.25E-04	2.37E-02	2.17E-01
Robinsoniella_B	Moryella_B	8.50E-01	3.46E-04	1.97E-02	2.17E-01
Otidea_F	Myceligenerans_B	1.00E+00	2.22E-16	2.21E-14	1.90E-06
Quatrionicoccus_B	Myceligenerans_B	1.00E+00	2.22E-16	2.21E-14	1.90E-06
Roseospirillum_B	Myceligenerans_B	1.00E+00	2.22E-16	2.21E-14	1.90E-06
Thermodesulfobium_B	Myceligenerans_B	1.00E+00	2.22E-16	2.21E-14	1.90E-06
Yaniella_B	Myceligenerans_B	1.00E+00	2.22E-16	2.21E-14	1.90E-06
Tepidimicrobium_B	Natantispora_F	1.00E+00	2.22E-16	2.21E-14	1.90E-06
Thermotalea_B	Natantispora_F	8.71E-01	1.66E-04	1.01E-02	1.04E-01
Nubsella_B	Nesiotobacter_B	8.16E-01	9.31E-04	4.65E-02	2.68E-01
Oxalicibacterium_B	Nesiotobacter_B	1.00E+00	2.22E-16	2.21E-14	1.90E-06
Paracoccus_B	Nesiotobacter_B	1.00E+00	2.22E-16	2.21E-14	1.90E-06
Paralactobacillus_B	Nesiotobacter_B	1.00E+00	2.22E-16	2.21E-14	1.90E-06
Proteiniphilum_B	Nesiotobacter_B	1.00E+00	2.22E-16	2.21E-14	1.90E-06
Psychrobacter_B	Nesiotobacter_B	1.00E+00	2.22E-16	2.21E-14	1.90E-06
Sediminibacterium_B	Nesiotobacter_B	1.00E+00	2.22E-16	2.21E-14	1.90E-06
Selenomonas_B	Nesiotobacter_B	1.00E+00	2.22E-16	2.21E-14	1.90E-06
Stenoxybacter_B	Nesiotobacter_B	1.00E+00	2.22E-16	2.21E-14	1.90E-06
Talaromyces_F	Nesiotobacter_B	1.00E+00	2.22E-16	2.21E-14	1.90E-06
unclassified_Pezizomycotina_F	Nesiotobacter_B	-1.00E+00	2.22E-16	2.21E-14	1.90E-06
Okibacterium_B	Nicoletella_B	1.00E+00	2.22E-16	2.21E-14	1.90E-06
Olsenella_B	Nicoletella_B	-9.47E-01	1.78E-06	1.57E-04	2.81E-03
Parascardovia_B	Nicoletella_B	1.00E+00	2.22E-16	2.21E-14	1.90E-06

Rhodovarius_B	Nicoletella_B	1.00E+00	2.22E-16	2.21E-14	1.90E-06
Slackia_B	Nicoletella_B	-9.07E-01	3.20E-05	2.25E-03	4.17E-02
Thermovenabulum_B	Nicoletella_B	1.00E+00	2.22E-16	2.21E-14	1.90E-06
Olivibacter_B	Nocardioides_B	-9.17E-01	1.75E-05	1.30E-03	7.84E-03
Pelomonas_B	Nocardioides_B	1.00E+00	2.22E-16	2.21E-14	1.90E-06
Unc_Kirschsteiniothelia_F	Nocardioides_B	-1.00E+00	2.22E-16	2.21E-14	1.90E-06
Verrucomicrobium_B	Nocardioides_B	-8.93E-01	6.47E-05	4.33E-03	6.01E-02
Rubritepida_B	Novosphingobium_B	1.00E+00	2.22E-16	2.21E-14	1.90E-06
Segetibacter_B	Novosphingobium_B	-9.30E-01	7.53E-06	6.16E-04	7.84E-03
Shimazuella_B	Novosphingobium_B	9.91E-01	1.88E-10	1.85E-08	5.47E-06
Zhouia_B	Novosphingobium_B	-9.73E-01	5.89E-08	5.63E-06	2.87E-04
Oxalicibacterium_B	Nubsella_B	8.16E-01	9.31E-04	4.65E-02	2.68E-01
Paracoccus_B	Nubsella_B	8.16E-01	9.31E-04	4.65E-02	2.68E-01
Paralactobacillus_B	Nubsella_B	8.16E-01	9.31E-04	4.65E-02	2.68E-01
Proteiniphilum_B	Nubsella_B	8.16E-01	9.31E-04	4.65E-02	2.68E-01
Psychrobacter_B	Nubsella_B	8.16E-01	9.31E-04	4.65E-02	2.68E-01
Sediminibacterium_B	Nubsella_B	8.16E-01	9.31E-04	4.65E-02	2.68E-01
Selenomonas_B	Nubsella_B	8.16E-01	9.31E-04	4.65E-02	2.68E-01
Sphingobacterium_B	Nubsella_B	8.48E-01	3.68E-04	2.08E-02	2.17E-01
Stenoxybacter_B	Nubsella_B	8.16E-01	9.31E-04	4.65E-02	2.68E-01
Talaromyces_F	Nubsella_B	8.16E-01	9.31E-04	4.65E-02	2.68E-01
unclassified_Pezizomycotina_F	Nubsella_B	-8.16E-01	9.31E-04	4.65E-02	2.68E-01
Olsenella_B	Okibacterium_B	-9.47E-01	1.78E-06	1.57E-04	2.81E-03
Parascardovia_B	Okibacterium_B	1.00E+00	2.22E-16	2.21E-14	1.90E-06
Rhodovarius_B	Okibacterium_B	1.00E+00	2.22E-16	2.21E-14	1.90E-06
Slackia_B	Okibacterium_B	-9.07E-01	3.20E-05	2.25E-03	4.17E-02
Thermovenabulum_B	Okibacterium_B	1.00E+00	2.22E-16	2.21E-14	1.90E-06
Pelomonas_B	Olivibacter_B	-9.17E-01	1.75E-05	1.30E-03	7.84E-03
Unc_Kirschsteiniothelia_F	Olivibacter_B	9.17E-01	1.75E-05	1.30E-03	7.84E-03
Parascardovia_B	Olsenella_B	-9.47E-01	1.78E-06	1.57E-04	2.81E-03

Rhodovarius_B	Olsenella_B	-9.47E-01	1.78E-06	1.57E-04	2.81E-03
Slackia_B	Olsenella_B	8.48E-01	3.69E-04	2.09E-02	2.17E-01
Thermovenabulum_B	Olsenella_B	-9.47E-01	1.78E-06	1.57E-04	2.81E-03
Sporobacterium_B	Oribacterium_B	8.16E-01	9.51E-04	4.73E-02	2.68E-01
Staphylococcus_B	Oribacterium_B	-8.14E-01	9.84E-04	4.86E-02	2.68E-01
Stenotrophomonas_B	Oribacterium_B	-9.07E-01	3.17E-05	2.22E-03	3.13E-02
Oxalophagus_B	Ornithinimicrobium_B	-9.54E-01	8.65E-07	7.82E-05	2.81E-03
Pseudozobellia_B	Ornithinimicrobium_B	-9.16E-01	1.90E-05	1.39E-03	1.77E-02
Rhodopila_B	Ornithinimicrobium_B	1.00E+00	2.22E-16	2.21E-14	1.90E-06
Streptophyta_B	Ornithinimicrobium_B	-9.16E-01	1.90E-05	1.39E-03	1.77E-02
Trichococcus_B	Ornithinimicrobium_B	1.00E+00	2.22E-16	2.21E-14	1.90E-06
Parachlamydia_B	Ornithobacterium_B	-9.11E-01	2.50E-05	1.78E-03	3.13E-02
Sphaerobacter_B	Ornithobacterium_B	-9.11E-01	2.50E-05	1.78E-03	3.13E-02
Sporanaerobacter_B	Ornithobacterium_B	-9.11E-01	2.50E-05	1.78E-03	3.13E-02
Unc_Kockovaella_F	Ornithobacterium_B	-9.11E-01	2.50E-05	1.78E-03	3.13E-02
Venenivibrio_B	Ornithobacterium_B	-9.11E-01	2.50E-05	1.78E-03	3.13E-02
Zunongwangia_B	Ornithobacterium_B	-9.11E-01	2.50E-05	1.78E-03	3.13E-02
Owenweeksia_B	Oscillibacter_B	8.34E-01	5.63E-04	3.04E-02	2.17E-01
Quatrionicoccus_B	Otidea_F	1.00E+00	2.22E-16	2.21E-14	1.90E-06
Roseospirillum_B	Otidea_F	1.00E+00	2.22E-16	2.21E-14	1.90E-06
Thermodesulfobium_B	Otidea_F	1.00E+00	2.22E-16	2.21E-14	1.90E-06
Yaniella_B	Otidea_F	1.00E+00	2.22E-16	2.21E-14	1.90E-06
Parasporobacterium_B	Owenweeksia_B	8.17E-01	9.07E-04	4.55E-02	2.68E-01
Succiniclasticum_B	Owenweeksia_B	8.45E-01	4.02E-04	2.26E-02	2.17E-01
Paracoccus_B	Oxalicibacterium_B	1.00E+00	2.22E-16	2.21E-14	1.90E-06
Paralactobacillus_B	Oxalicibacterium_B	1.00E+00	2.22E-16	2.21E-14	1.90E-06
Proteiniphilum_B	Oxalicibacterium_B	1.00E+00	2.22E-16	2.21E-14	1.90E-06
Psychrobacter_B	Oxalicibacterium_B	1.00E+00	2.22E-16	2.21E-14	1.90E-06
Sediminibacterium_B	Oxalicibacterium_B	1.00E+00	2.22E-16	2.21E-14	1.90E-06
Selenomonas_B	Oxalicibacterium_B	1.00E+00	2.22E-16	2.21E-14	1.90E-06

Stenoxybacter_B	Oxalicibacterium_B	1.00E+00	2.22E-16	2.21E-14	1.90E-06
Talaromyces_F	Oxalicibacterium_B	1.00E+00	2.22E-16	2.21E-14	1.90E-06
unclassified_Pezizomycotina_F	Oxalicibacterium_B	-1.00E+00	2.22E-16	2.21E-14	1.90E-06
Pseudozobellia_B	Oxalophagus_B	8.94E-01	6.18E-05	4.15E-03	6.01E-02
Rhodopila_B	Oxalophagus_B	-9.54E-01	8.65E-07	7.82E-05	2.81E-03
Streptophyta_B	Oxalophagus_B	8.64E-01	2.13E-04	1.26E-02	1.04E-01
Trichococcus_B	Oxalophagus_B	-9.54E-01	8.65E-07	7.82E-05	2.81E-03
Unc_Sarcinomyces_F	Paludibacter_B	-8.85E-01	9.21E-05	5.99E-03	6.01E-02
Sharpea_B	Papillibacter_B	8.77E-01	1.30E-04	8.13E-03	6.06E-02
Sphaerobacter_B	Parachlamydia_B	1.00E+00	2.22E-16	2.21E-14	1.90E-06
Sporanaerobacter_B	Parachlamydia_B	1.00E+00	2.22E-16	2.21E-14	1.90E-06
Unc_Kockovaella_F	Parachlamydia_B	1.00E+00	2.22E-16	2.21E-14	1.90E-06
Venenivibrio_B	Parachlamydia_B	1.00E+00	2.22E-16	2.21E-14	1.90E-06
Zunongwangia_B	Parachlamydia_B	1.00E+00	2.22E-16	2.21E-14	1.90E-06
Paralactobacillus_B	Paracoccus_B	1.00E+00	2.22E-16	2.21E-14	1.90E-06
Proteiniphilum_B	Paracoccus_B	1.00E+00	2.22E-16	2.21E-14	1.90E-06
Psychrobacter_B	Paracoccus_B	1.00E+00	2.22E-16	2.21E-14	1.90E-06
Sediminibacterium_B	Paracoccus_B	1.00E+00	2.22E-16	2.21E-14	1.90E-06
Selenomonas_B	Paracoccus_B	1.00E+00	2.22E-16	2.21E-14	1.90E-06
Stenoxybacter_B	Paracoccus_B	1.00E+00	2.22E-16	2.21E-14	1.90E-06
Talaromyces_F	Paracoccus_B	1.00E+00	2.22E-16	2.21E-14	1.90E-06
unclassified_Pezizomycotina_F	Paracoccus_B	-1.00E+00	2.22E-16	2.21E-14	1.90E-06
Proteiniphilum_B	Paralactobacillus_B	1.00E+00	2.22E-16	2.21E-14	1.90E-06
Psychrobacter_B	Paralactobacillus_B	1.00E+00	2.22E-16	2.21E-14	1.90E-06
Sediminibacterium_B	Paralactobacillus_B	1.00E+00	2.22E-16	2.21E-14	1.90E-06
Selenomonas_B	Paralactobacillus_B	1.00E+00	2.22E-16	2.21E-14	1.90E-06
Stenoxybacter_B	Paralactobacillus_B	1.00E+00	2.22E-16	2.21E-14	1.90E-06
Talaromyces_F	Paralactobacillus_B	1.00E+00	2.22E-16	2.21E-14	1.90E-06
unclassified_Pezizomycotina_F	Paralactobacillus_B	-1.00E+00	2.22E-16	2.21E-14	1.90E-06
Sporacetigenium_B	Parapedobacter_B	-9.34E-01	5.64E-06	4.71E-04	7.84E-03

Stenothermobacter_B	Parapedobacter_B	1.00E+00	2.22E-16	2.21E-14	1.90E-06
Rhodovarius_B	Parascardovia_B	1.00E+00	2.22E-16	2.21E-14	1.90E-06
Slackia_B	Parascardovia_B	-9.07E-01	3.20E-05	2.25E-03	4.17E-02
Thermovenabulum_B	Parascardovia_B	1.00E+00	2.22E-16	2.21E-14	1.90E-06
Schwartzia_B	Parasegetibacter_B	9.34E-01	5.46E-06	4.57E-04	7.84E-03
Tepidanaerobacter_B	Parasegetibacter_B	-9.42E-01	2.86E-06	2.46E-04	7.84E-03
Thermoflavimicrobium_B	Parasegetibacter_B	-9.42E-01	2.86E-06	2.46E-04	7.84E-03
Unc_Verticillium_F	Parasegetibacter_B	-9.42E-01	2.86E-06	2.46E-04	7.84E-03
Phascolarctobacterium_B	Parasporobacterium_B	8.63E-01	2.19E-04	1.29E-02	2.17E-01
Pirellula_B	Parasporobacterium_B	8.63E-01	2.19E-04	1.29E-02	2.17E-01
Rudanella_B	Parasporobacterium_B	8.63E-01	2.19E-04	1.29E-02	2.17E-01
Succiniclasticum_B	Parasporobacterium_B	8.19E-01	8.76E-04	4.42E-02	2.68E-01
Thermonema_B	Parasporobacterium_B	8.63E-01	2.19E-04	1.29E-02	2.17E-01
Tropheryma_B	Parasporobacterium_B	8.63E-01	2.19E-04	1.29E-02	2.17E-01
Unc_Cryptococcus_F	Parasporobacterium_B	8.63E-01	2.19E-04	1.29E-02	2.17E-01
Verminephrobacter_B	Parasporobacterium_B	8.63E-01	2.19E-04	1.29E-02	2.17E-01
Scytinostroma_F	Parvimonas_B	-9.47E-01	1.86E-06	1.63E-04	4.74E-03
Unc_Kirschsteiniothelia_F	Pelomonas_B	-1.00E+00	2.22E-16	2.21E-14	1.90E-06
Verrucomicrobium_B	Pelomonas_B	-8.93E-01	6.47E-05	4.33E-03	6.01E-02
Sporobacterium_B	Pelospora_B	9.46E-01	1.96E-06	1.71E-04	4.74E-03
Stenotrophomonas_B	Pelospora_B	-9.38E-01	3.98E-06	3.38E-04	7.84E-03
Tannerella_B	Pelospora_B	8.99E-01	4.95E-05	3.38E-03	4.24E-02
Planococcus_B	Penicilliopsis_F	1.00E+00	2.22E-16	2.21E-14	1.90E-06
Selenihalanaerobacter_B	Peptostreptococcus_B	-9.51E-01	1.20E-06	1.08E-04	2.81E-03
Prolixibacter_B	Peredibacter_B	-8.19E-01	8.59E-04	4.35E-02	2.68E-01
Pseudobutyrivibrio_B	Persicitalea_B	-9.35E-01	5.27E-06	4.42E-04	7.84E-03
Porphyromonas_B	Petrimonas_B	1.00E+00	2.22E-16	2.21E-14	1.90E-06
Propionibacterium_B	Petrimonas_B	1.00E+00	2.22E-16	2.21E-14	1.90E-06
Pseudomonas_B	Petrimonas_B	1.00E+00	2.22E-16	2.21E-14	1.90E-06
Sanguibacter_B	Petrimonas_B	1.00E+00	2.22E-16	2.21E-14	1.90E-06

Shuttleworthia_B	Petrimonas_B	-8.88E-01	8.23E-05	5.41E-03	6.01E-02
Spirochaeta_B	Petrimonas_B	1.00E+00	2.22E-16	2.21E-14	1.90E-06
unclassified_F	Petrimonas_B	-8.44E-01	4.25E-04	2.37E-02	2.17E-01
Pirellula_B	Phascolarctobacterium_B	1.00E+00	2.22E-16	2.21E-14	1.90E-06
Rhodopirellula_B	Phascolarctobacterium_B	-8.76E-01	1.33E-04	8.28E-03	7.38E-02
Rudanella_B	Phascolarctobacterium_B	1.00E+00	2.22E-16	2.21E-14	1.90E-06
Thermonema_B	Phascolarctobacterium_B	1.00E+00	2.22E-16	2.21E-14	1.90E-06
Tropheryma_B	Phascolarctobacterium_B	1.00E+00	2.22E-16	2.21E-14	1.90E-06
Unc_Cryptococcus_F	Phascolarctobacterium_B	1.00E+00	2.22E-16	2.21E-14	1.90E-06
Verminephrobacter_B	Phascolarctobacterium_B	1.00E+00	2.22E-16	2.21E-14	1.90E-06
Rhodopirellula_B	Pirellula_B	-8.76E-01	1.33E-04	8.28E-03	7.38E-02
Rudanella_B	Pirellula_B	1.00E+00	2.22E-16	2.21E-14	1.90E-06
Thermonema_B	Pirellula_B	1.00E+00	2.22E-16	2.21E-14	1.90E-06
Tropheryma_B	Pirellula_B	1.00E+00	2.22E-16	2.21E-14	1.90E-06
Unc_Cryptococcus_F	Pirellula_B	1.00E+00	2.22E-16	2.21E-14	1.90E-06
Verminephrobacter_B	Pirellula_B	1.00E+00	2.22E-16	2.21E-14	1.90E-06
Propionibacterium_B	Porphyromonas_B	1.00E+00	2.22E-16	2.21E-14	1.90E-06
Pseudomonas_B	Porphyromonas_B	1.00E+00	2.22E-16	2.21E-14	1.90E-06
Sanguibacter_B	Porphyromonas_B	1.00E+00	2.22E-16	2.21E-14	1.90E-06
Shuttleworthia_B	Porphyromonas_B	-8.88E-01	8.23E-05	5.41E-03	6.01E-02
Spirochaeta_B	Porphyromonas_B	1.00E+00	2.22E-16	2.21E-14	1.90E-06
unclassified_F	Porphyromonas_B	-8.44E-01	4.25E-04	2.37E-02	2.17E-01
Rhodothermus_B	Prolixibacter_B	-9.86E-01	1.64E-09	1.60E-07	2.89E-05
Pseudomonas_B	Propionibacterium_B	1.00E+00	2.22E-16	2.21E-14	1.90E-06
Sanguibacter_B	Propionibacterium_B	1.00E+00	2.22E-16	2.21E-14	1.90E-06
Shuttleworthia_B	Propionibacterium_B	-8.88E-01	8.23E-05	5.41E-03	6.01E-02
Spirochaeta_B	Propionibacterium_B	1.00E+00	2.22E-16	2.21E-14	1.90E-06
unclassified_F	Propionibacterium_B	-8.44E-01	4.25E-04	2.37E-02	2.17E-01
Psychrobacter_B	Proteiniphilum_B	1.00E+00	2.22E-16	2.21E-14	1.90E-06
Sediminibacterium_B	Proteiniphilum_B	1.00E+00	2.22E-16	2.21E-14	1.90E-06

Selenomonas_B	Proteiniphilum_B	1.00E+00	2.22E-16	2.21E-14	1.90E-06
Stenoxybacter_B	Proteiniphilum_B	1.00E+00	2.22E-16	2.21E-14	1.90E-06
Talaromyces_F	Proteiniphilum_B	1.00E+00	2.22E-16	2.21E-14	1.90E-06
unclassified_Pezizomycotina_F	Proteiniphilum_B	-1.00E+00	2.22E-16	2.21E-14	1.90E-06
Robinsoniella_B	Protomyces_F	8.56E-01	2.85E-04	1.65E-02	2.17E-01
Ruminococcus_B	Pseudobutyrivibrio_B	8.30E-01	6.41E-04	3.39E-02	2.17E-01
Sanguibacter_B	Pseudomonas_B	1.00E+00	2.22E-16	2.21E-14	1.90E-06
Shuttleworthia_B	Pseudomonas_B	-8.88E-01	8.23E-05	5.41E-03	6.01E-02
Spirochaeta_B	Pseudomonas_B	1.00E+00	2.22E-16	2.21E-14	1.90E-06
unclassified_F	Pseudomonas_B	-8.44E-01	4.25E-04	2.37E-02	2.17E-01
Roseburia_B	Pseudosphingobacterium_B	9.41E-01	3.19E-06	2.73E-04	7.84E-03
Syntrophococcus_B	Pseudosphingobacterium_B	8.54E-01	3.08E-04	1.77E-02	2.17E-01
Rhodopila_B	Pseudozobellia_B	-9.16E-01	1.90E-05	1.39E-03	1.77E-02
Streptophyta_B	Pseudozobellia_B	8.38E-01	5.04E-04	2.76E-02	2.17E-01
Trichococcus_B	Pseudozobellia_B	-9.16E-01	1.90E-05	1.39E-03	1.77E-02
Sediminibacterium_B	Psychrobacter_B	1.00E+00	2.22E-16	2.21E-14	1.90E-06
Selenomonas_B	Psychrobacter_B	1.00E+00	2.22E-16	2.21E-14	1.90E-06
Stenoxybacter_B	Psychrobacter_B	1.00E+00	2.22E-16	2.21E-14	1.90E-06
Talaromyces_F	Psychrobacter_B	1.00E+00	2.22E-16	2.21E-14	1.90E-06
unclassified_Pezizomycotina_F	Psychrobacter_B	-1.00E+00	2.22E-16	2.21E-14	1.90E-06
Sarcina_B	Pyramidobacter_B	-8.15E-01	9.66E-04	4.79E-02	2.68E-01
Roseospirillum_B	Quatrionicoccus_B	1.00E+00	2.22E-16	2.21E-14	1.90E-06
Thermodesulfobium_B	Quatrionicoccus_B	1.00E+00	2.22E-16	2.21E-14	1.90E-06
Yaniella_B	Quatrionicoccus_B	1.00E+00	2.22E-16	2.21E-14	1.90E-06
Rikenella_B	Rapidithrix_B	8.22E-01	8.07E-04	4.12E-02	2.68E-01
Solibacillus_B	Rapidithrix_B	1.00E+00	2.22E-16	2.21E-14	1.90E-06
Streptophyta_B	Rhodopila_B	-9.16E-01	1.90E-05	1.39E-03	1.77E-02
Trichococcus_B	Rhodopila_B	1.00E+00	2.22E-16	2.21E-14	1.90E-06
Rudanella_B	Rhodopirellula_B	-8.76E-01	1.33E-04	8.28E-03	7.38E-02
Thermonema_B	Rhodopirellula_B	-8.76E-01	1.33E-04	8.28E-03	7.38E-02

Tropheryma_B	Rhodopirellula_B	-8.76E-01	1.33E-04	8.28E-03	7.38E-02
Unc_Cryptococcus_F	Rhodopirellula_B	-8.76E-01	1.33E-04	8.28E-03	7.38E-02
Verminephrobacter_B	Rhodopirellula_B	-8.76E-01	1.33E-04	8.28E-03	7.38E-02
Slackia_B	Rhodovarius_B	-9.07E-01	3.20E-05	2.25E-03	4.17E-02
Thermovenabulum_B	Rhodovarius_B	1.00E+00	2.22E-16	2.21E-14	1.90E-06
Solibacillus_B	Rikenella_B	8.22E-01	8.07E-04	4.12E-02	2.68E-01
Solobacterium_B	Rikenella_B	9.00E-01	4.56E-05	3.13E-03	4.17E-02
Syntrophococcus_B	Roseburia_B	8.97E-01	5.22E-05	3.54E-03	6.01E-02
Thermodesulfobium_B	Roseospirillum_B	1.00E+00	2.22E-16	2.21E-14	1.90E-06
Yaniella_B	Roseospirillum_B	1.00E+00	2.22E-16	2.21E-14	1.90E-06
Segetibacter_B	Rubritepida_B	-9.30E-01	7.53E-06	6.16E-04	7.84E-03
Shimazuella_B	Rubritepida_B	9.91E-01	1.88E-10	1.85E-08	5.47E-06
Zhouia_B	Rubritepida_B	-9.73E-01	5.89E-08	5.63E-06	2.87E-04
Thermonema_B	Rudanella_B	1.00E+00	2.22E-16	2.21E-14	1.90E-06
Tropheryma_B	Rudanella_B	1.00E+00	2.22E-16	2.21E-14	1.90E-06
Unc_Cryptococcus_F	Rudanella_B	1.00E+00	2.22E-16	2.21E-14	1.90E-06
Verminephrobacter_B	Rudanella_B	1.00E+00	2.22E-16	2.21E-14	1.90E-06
Scytinostroma_F	Ruminococcus_B	8.26E-01	7.17E-04	3.73E-02	2.68E-01
Shuttleworthia_B	Sanguibacter_B	-8.88E-01	8.23E-05	5.41E-03	6.01E-02
Spirochaeta_B	Sanguibacter_B	1.00E+00	2.22E-16	2.21E-14	1.90E-06
unclassified_F	Sanguibacter_B	-8.44E-01	4.25E-04	2.37E-02	2.17E-01
Tepidanaerobacter_B	Schwartzia_B	-9.26E-01	9.83E-06	7.85E-04	7.84E-03
Thermoflavimicrobium_B	Schwartzia_B	-9.26E-01	9.83E-06	7.85E-04	7.84E-03
Unc_Verticillium_F	Schwartzia_B	-9.26E-01	9.83E-06	7.85E-04	7.84E-03
Sediminitomix_B	Sedimentibacter_B	-8.18E-01	8.87E-04	4.46E-02	2.68E-01
Xylanibacter_B	Sedimentibacter_B	8.73E-01	1.50E-04	9.20E-03	7.38E-02
Selenomonas_B	Sediminibacterium_B	1.00E+00	2.22E-16	2.21E-14	1.90E-06
Stenoxybacter_B	Sediminibacterium_B	1.00E+00	2.22E-16	2.21E-14	1.90E-06
Talaromyces_F	Sediminibacterium_B	1.00E+00	2.22E-16	2.21E-14	1.90E-06
unclassified_Pezizomycotina_F	Sediminibacterium_B	-1.00E+00	2.22E-16	2.21E-14	1.90E-06

Syntrophothermus_B	Sediminicola_B	9.91E-01	1.88E-10	1.85E-08	5.47E-06
Tumebacillus_B	Sediminitomix_B	-9.90E-01	3.42E-10	3.36E-08	7.72E-06
Shimazuella_B	Segetibacter_B	-9.22E-01	1.33E-05	1.03E-03	7.84E-03
Zhouia_B	Segetibacter_B	8.53E-01	3.14E-04	1.81E-02	2.17E-01
Stenoxybacter_B	Selenomonas_B	1.00E+00	2.22E-16	2.21E-14	1.90E-06
Talaromyces_F	Selenomonas_B	1.00E+00	2.22E-16	2.21E-14	1.90E-06
unclassified_Pezizomycotina_F	Selenomonas_B	-1.00E+00	2.22E-16	2.21E-14	1.90E-06
Zhouia_B	Shimazuella_B	-9.64E-01	2.47E-07	2.29E-05	2.21E-03
Spirochaeta_B	Shuttleworthia_B	-8.88E-01	8.23E-05	5.41E-03	6.01E-02
Syntrophococcus_B	Shuttleworthia_B	8.67E-01	1.92E-04	1.15E-02	1.04E-01
unclassified_F	Shuttleworthia_B	8.38E-01	5.05E-04	2.76E-02	2.17E-01
Thermovenabulum_B	Slackia_B	-9.07E-01	3.20E-05	2.25E-03	4.17E-02
Sporanaerobacter_B	Sphaerobacter_B	1.00E+00	2.22E-16	2.21E-14	1.90E-06
Unc_Kockovaella_F	Sphaerobacter_B	1.00E+00	2.22E-16	2.21E-14	1.90E-06
Venenivibrio_B	Sphaerobacter_B	1.00E+00	2.22E-16	2.21E-14	1.90E-06
Zunongwangia_B	Sphaerobacter_B	1.00E+00	2.22E-16	2.21E-14	1.90E-06
Victivallis_B	Sphingomonas_B	9.85E-01	2.45E-09	2.39E-07	2.87E-04
unclassified_F	Spirochaeta_B	-8.44E-01	4.25E-04	2.37E-02	2.17E-01
Stenothermobacter_B	Sporacetigenium_B	-9.34E-01	5.64E-06	4.71E-04	7.84E-03
Unc_Kockovaella_F	Sporanaerobacter_B	1.00E+00	2.22E-16	2.21E-14	1.90E-06
Venenivibrio_B	Sporanaerobacter_B	1.00E+00	2.22E-16	2.21E-14	1.90E-06
Zunongwangia_B	Sporanaerobacter_B	1.00E+00	2.22E-16	2.21E-14	1.90E-06
Stenotrophomonas_B	Sporobacterium_B	-9.77E-01	2.32E-08	2.25E-06	2.87E-04
Vulcanibacillus_B	Staphylococcus_B	8.36E-01	5.31E-04	2.89E-02	2.17E-01
Talaromyces_F	Stenoxybacter_B	1.00E+00	2.22E-16	2.21E-14	1.90E-06
unclassified_Pezizomycotina_F	Stenoxybacter_B	-1.00E+00	2.22E-16	2.21E-14	1.90E-06
Trichococcus_B	Streptophyta_B	-9.16E-01	1.90E-05	1.39E-03	1.77E-02
Taphrina_F	Succinivibrio_B	1.00E+00	2.22E-16	2.21E-14	1.90E-06
Turicibacter_B	Syntrophococcus_B	-8.24E-01	7.49E-04	3.87E-02	2.68E-01
unclassified_Pezizomycotina_F	Talaromyces_F	-1.00E+00	2.22E-16	2.21E-14	1.90E-06

Thermoflavimicrobium_B	Tepidanaerobacter_B	1.00E+00	2.22E-16	2.21E-14	1.90E-06
Unc_Verticillium_F	Tepidanaerobacter_B	1.00E+00	2.22E-16	2.21E-14	1.90E-06
Thermotalea_B	Tepidimicrobium_B	8.71E-01	1.66E-04	1.01E-02	1.04E-01
Victivallis_B	Thermicanus_B	8.54E-01	3.02E-04	1.74E-02	2.17E-01
Yaniella_B	Thermodesulfobium_B	1.00E+00	2.22E-16	2.21E-14	1.90E-06
Unc_Verticillium_F	Thermoflavimicrobium_B	1.00E+00	2.22E-16	2.21E-14	1.90E-06
Tropheryma_B	Thermonema_B	1.00E+00	2.22E-16	2.21E-14	1.90E-06
Unc_Cryptococcus_F	Thermonema_B	1.00E+00	2.22E-16	2.21E-14	1.90E-06
Verminephrobacter_B	Thermonema_B	1.00E+00	2.22E-16	2.21E-14	1.90E-06
Unc_Cryptococcus_F	Tropheryma_B	1.00E+00	2.22E-16	2.21E-14	1.90E-06
Verminephrobacter_B	Tropheryma_B	1.00E+00	2.22E-16	2.21E-14	1.90E-06
Verminephrobacter_B	Unc_Cryptococcus_F	1.00E+00	2.22E-16	2.21E-14	1.90E-06
Verrucomicrobium_B	Unc_Kirschsteiniothelia_F	8.93E-01	6.47E-05	4.33E-03	6.01E-02
Venenivibrio_B	Unc_Kockovaella_F	1.00E+00	2.22E-16	2.21E-14	1.90E-06
Zunongwangia_B	Unc_Kockovaella_F	1.00E+00	2.22E-16	2.21E-14	1.90E-06
Zunongwangia_B	Venenivibrio_B	1.00E+00	2.22E-16	2.21E-14	1.90E-06
