# Supplementary material for: Next Generation Sequencing to Define Prokaryotic and Fungal Diversity in the Bovine Rumen
Source: PLoS One. 2012 Nov 7;7(11):e48289. doi: 10.1371/journal.pone.0048289 (PMC3492333; doi:10.1371/journal.pone.0048289)
Supplement: Table S4 — Statistically significant cross-domain pairwise correlations between bacterial, archaeal and fungal classes. (PDF) [file pone.0048289.s008.pdf]

Table S4

a	b	r	pval	qval	lfdr
Verrucomicrobia_Subdivision3_B	Anaerolineae_B	-8.93E-01	4.01E-04	4.75E-02	1.41E-01
Thermomicrobia_B	Aquificae_B	1.00E+00	2.22E-16	5.48E-14	3.50E-04
Thermotogae_B	Unclassified_Bacteroidetes_B	-9.82E-01	2.83E-07	5.83E-05	1.36E-02
Unclassified_Pezizomycotina_F	Caldilineae_B	-1.00E+00	2.22E-16	5.48E-14	3.50E-04
Spirochaetia_B	Fibrobacteria_B	8.95E-01	3.67E-04	4.48E-02	1.41E-01
Tremellomycetes_F	Gemmatimonadetes_B	-9.56E-01	1.13E-05	1.99E-03	1.41E-01
Pezizomycetes_F	Halobacteria_A	1.00E+00	2.22E-16	5.48E-14	3.50E-04
Thermoprotei_A	Halobacteria_A	1.00E+00	2.22E-16	5.48E-14	3.50E-04
Thermoprotei_A	Pezizomycetes_F	1.00E+00	2.22E-16	5.48E-14	3.50E-04
Verrucomicrobiae_B	Tremellomycetes_F	9.01E-01	2.92E-04	3.81E-02	1.41E-01
