# Supplementary material for: Next Generation Sequencing to Define Prokaryotic and Fungal Diversity in the Bovine Rumen
Source: PLoS One. 2012 Nov 7;7(11):e48289. doi: 10.1371/journal.pone.0048289 (PMC3492333; doi:10.1371/journal.pone.0048289)
Supplement: Text S1 — Supplemental methods describing animal breed, animal care, sample harvesting and processing, DNA purification, PCR conditions, and sequencing. (DOCX) [file pone.0048289.s009.docx]

**Supplemental Methods**

**Animals:** Cows C1-12 were penned in a drylot at Fort Keogh LARRL with ad-libitum access to water and received a forage (Alfalfa/grass hay) based diet that also included a trace mineral and vitamin mix [91.0% DM (Dry Matter), 13.5% crude protein, 57.3% TDN (Total Digestible Nutrients), 1.23 Mcal/kg of NEm (Net Energy for Maintenance), 0.04% sulfur, 0.06% phosphorus, 0.58% potassium, 0.05% magnesium, 0.17% calcium, 907 ppm manganese, 230 ppm copper, and 747 ppm zinc]. Raw bolus samples were collected at 4 hr following feeding to optimize microbial growth curve dynamics.

**Sample preparation:** Rumen fluid was obtained from the midventral portion of the interface of the fiber mat and fluid layer immediately following hand-mixing procedure. Approximately one liter of rumen fluid was filtered through four layers of 90 grade cheesecloth. The retained solid containing plant, plant adherent microbiota and residual liquid was designated solid and the filtrate labeled liquid. All samples were transported on dry ice to the JCVI in Rockville, MD for DNA extraction and analysis. Protocols for DNA extraction, PCR, and cloning are derived from Yu and Morrison (Yu and Morrison 2004) and described in the subsequent sections.

**DNA Purification:** Rumen samples from above were homogenized with a bead-beater (Fastprep FP120). The samples were then treated with lysozyme and underwent several freeze thaw cycles followed by the addition of Proteinase K, SDS. DNA extraction was completed using Phenol/Chloroform/Isoamyl alcohol (PCI) followed by ethanol precipitation.

**PCR Conditions:** For each PCR, approximately 50 ng of the extracted DNA was used as template in a 50 μl reaction volume. The template DNA was added to a PCR mixture containing 5 μl of 10X PCR buffer (Invitrogen), 1.5 μl of 50 mM MgCl_2_, 1 μl of 10 mM dNTPs, 2 μl each of forward and reverse primers at a concentration of 10 μM, and 5 units of Platinum Taq DNA polymerase (Invitrogen). Reactions for eukaryotic 18S amplification were run at 94 °C for 2 min, followed by 35 cycles of amplification at 94 °C for 30 sec, 55 °C for 30 sec, and 72 °C for 2 min and a 7-min extension at 72 °C. Bacterial 16S products were amplified by running at 94 °C for 5 min, followed by 25 cycles of amplification at 94 °C for 30 sec, 60 °C for 30 sec, and 72 °C for 30 sec and a 7-min extension at 72 °C. Archaeal 16S products were produced by running at 94 °C for 2 min, followed by 35 cycles of amplification at 94 °C for 30 sec, 55 °C for 30 sec, and 72 °C for 45 sec plus a 7-min extension at 72 °C. The PCR products were separated from free PCR primers by using a PCR purification kit or gel purification kit (Qiagen).

**Cloning of archaeal 16S PCR products**: Archaeal 16S products were purified QIAquick PCR Purification Kit using and cloned (TOPO TA Cloning kit, Invitrogen) using manufacturer’s protocols. Cells were transformed using the following electroporation settings: 1.75 kV, 200 ohms, 25 μF. SOC media was added for cell recovery and glycerol was added to a final concentration of 10-20%. All transformations were stored at -80 ºC prior to sequencing.

**Sequencing:** The archaeal 16S amplicons were sequenced using with Sanger technology as estimates of rumen archaeal diversity are known to be less diverse than other fractions. The 16S and 18S amplicons were sequenced by 454 sequencing. Equimolar amounts of PCR amplicon were were pooled for 454 sequencing using DNA quantifications from the Tecan Spectrafluor Plus. A and B adaptors, which provided priming sequences for amplification and sequencing of amplicons, were ligated to the DNA fragments before the pooled and ligated sample underwent size-selection gel purification (Qiagen) prior to sequencing. The 16S and 18S pooled libraries then were sequenced separately on four half plates.
